# Toward an atlas of Salish Sea biodiversity: the flora and fauna of Galiano Island, British Columbia, Canada. Part I. Marine zoology

**DOI:** 10.3897/BDJ.10.e76050

**Published:** 2022-03-10

**Authors:** Andrew D. F. Simon, Emily M. Adamczyk, Antranig Basman, Jackson W. F. Chu, Heidi N. Gartner, Karin Fletcher, Charles J. Gibbs, Donna M. Gibbs, Scott R. Gilmore, Rick M. Harbo, Leslie H. Harris, Elaine Humphrey, Andy Lamb, Philip Lambert, Neil McDaniel, Jessica Scott, Brian M. Starzomski

**Affiliations:** 1 Institute for Multidisciplinary Ecological Research in the Salish Sea, Galiano Island, Canada Institute for Multidisciplinary Ecological Research in the Salish Sea Galiano Island Canada; 2 University of British Columbia, Vancouver, Canada University of British Columbia Vancouver Canada; 3 University of Victoria, Victoria, Canada University of Victoria Victoria Canada; 4 Royal British Columbia Museum, Victoria, Canada Royal British Columbia Museum Victoria Canada; 5 Port Orchard 98366, Port Orchard, United States of America Port Orchard 98366 Port Orchard United States of America; 6 Pacific Marine Life Surveys, Port Coquitlam, Canada Pacific Marine Life Surveys Port Coquitlam Canada; 7 7494 Andrea Cres, Lantzville, Canada 7494 Andrea Cres Lantzville Canada; 8 Natural History Museum of Los Angeles County, Los Angeles, United States of America Natural History Museum of Los Angeles County Los Angeles United States of America; 9 McDaniel Photography, Vancouver, Canada McDaniel Photography Vancouver Canada; 10 Ocean Wise, Vancouver, Canada Ocean Wise Vancouver Canada

**Keywords:** Salish Sea, Galiano Island, marine zoology, biodiversity, biodiversity informatics, inclusive design, open data, community science, community-integrated research

## Abstract

**Background:**

Based on records dating from 1859 to 2021, we provide an overview of the marine animal diversity reported for Galiano Island, British Columbia, Canada. More than 650 taxa are represented by 20,000 species occurrence records in this curated dataset, which includes dive records documented through the Pacific Marine Life Surveys, museum voucher specimens, ecological data and crowd-sourced observations from the BC Cetacean Sightings Network and iNaturalist.

**New information:**

We describe Galiano Island's marine animal diversity in relation to the Salish Sea's overall biodiversity and quantify the proportional contributions of different types of sampling effort to our current local knowledge. Overviews are provided for each taxonomic group in a format intended to be accessible to amateur naturalists interested in furthering research into the region's marine biodiversity. In summary, we find that the Pacific Marine Life Surveys, a regional community science diving initiative, account for 60% of novel records reported for Galiano Island. Voucher specimens account for 19% and crowd-sourced biodiversity data 18% of novel records, respectively, with the remaining 3% of reports coming from other sources. These findings shed light on the complementarity of different types of sampling effort and demonstrate the potential for community science to contribute to the global biodiversity research community. We present a biodiversity informatics framework that is designed to enable these practices by supporting collaboration among researchers and communities in the collection, curation and dissemination of biodiversity data.

## Introduction

**Figure 1.**
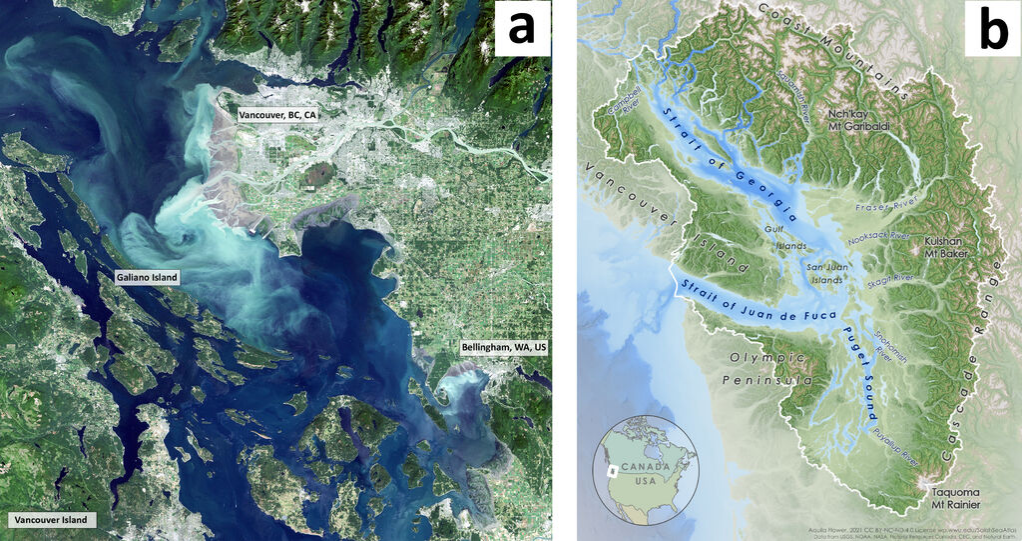
Study area: Galiano Island, British Columbia, Canada (Salish Sea bioregion) **a**. Satellite image showing Galiano Island in relation to major population centres. The Salish Sea, as a dynamic estuarine ecosystem, is illustrated in this enhanced image highlighting sediments from the Fraser and Nooksack Rivers. Imagery from Landsat-7 EMT+, 30 July 2000. Projection: UTM Zone 10 NAD83. Scale 1:200,000. – Image by Galiano Conservancy Association. **b**. Physical geography reference map for the Salish Sea bioregion. The Salish Sea bioregion includes both marine waters and their upland watersheds. These boundaries technically distinguish the Salish Sea from the Puget Sound/Georgia Basin marine ecoregion (sensu Spalding et al. 2007), which is a strictly marine classification. Data from USGS, NOAA, NASA, Natural Resources Canada, CEC and Natural Earth. – Map by Aquila Flower.

Galiano Island is located on the northwest coast of North America (48.9236°N, 123.4415°W) in a bioregion known as the Salish Sea, within the traditional territories of Penelakut, Hwlitsum and Tsawwassen First Nations, as well as other Hul’qumi’num-speaking peoples (Fig. 1). Otherwise known as the Puget Sound/Georgia Basin marine ecoregion, the Salish Sea is one of six biogeographically distinct ecoregions in the Cold Temperate Northeast Pacific marine province (Spalding et al. 2007). Home to nearly nine million people, the region is densely populated, its ecosystems and the traditional lifeways of Indigenous peoples threatened by expanding industry and development (Sobocinski 2021).

Galiano Island is part of the San Juan Archipelago, which spans the Canada-USA border in the rain shadow of the Olympic Mountains and Vancouver Island Ranges. This sheltered subregion of the Salish Sea has a Mediterranean-type climate that supports some of Canada’s most imperilled ecosystems, including the highest density of threatened species in British Columbia (Kraus and Hebb 2020, BC Conservation Data Centre 2021). The terrestrial ecosystems of this archipelago are subject to periodic climatic stress, with increasingly severe seasonal drought and more extreme winter precipitation forecasted under future climate scenarios (Moore et al. 2010, Klassen et al. 2015, Seager et al. 2019). Average marine conditions are predicted to become warmer, more acidic and more hypoxic by the end of the 21^st^ century, resulting in earlier onset of spring freshet and phytoplankton blooms, increased algal biomass and shifts in the planktonic community (Riche et al. 2014, Khangaonkar et al. 2019).

Given the increasing threats that anthropogenic climate change and development present for the Salish Sea (Sobocinski 2021), there is an urgent need to mobilise localised efforts to monitor its biodiversity. In this context, Galiano Island represents an ideal model system, demonstrating the potential for community science to help achieve this goal. Galiano Island’s biota have been intensively sampled historically, with more than 4,000 taxa recorded to date, including avian, freshwater, marine and terrestrial species. In this article, we provide an overview of the marine animal diversity reported from 1859 to 2021, including recent surveys coordinated through a community-based biodiversity project: Biodiversity Galiano. This dataset accounts for 666 taxa represented by 20,022 occurrence records, including dive records, voucher specimens, ecological data and crowd-sourced observations shared via iNaturalist and the BC Cetacean Sightings Network. We explore this dataset as an outcome of community-integrated biodiversity research practices, quantifying the contributions of various types of sampling effort to our cumulative knowledge of the island's biodiversity.

This article is the first instalment in a five-part series documenting Galiano Island’s flora and fauna. Each part in this series is intended to: a) establish a formal biodiversity baseline for Galiano Island; and b) elaborate an open source framework for organising, evaluating, analysing and sharing diverse sources of biodiversity data, which may be adapted as the basis for an atlas of Salish Sea biodiversity. Thus, while this project is being advanced with a focus on Galiano Island, it is intended to serve a broader purpose in the field of biodiversity informatics: to create a more inclusive and systematic framework for biodiversity research and monitoring. Community science has become an increasingly important source of data for international biodiversity monitoring efforts (Chandler et al. 2017), though its full potential has yet to be realised (Theobald et al. 2015). We endeavour to create a framework that harnesses this potential, to help increase capacity for community-integrated biodiversity research in the Salish Sea.

### Study Area Description

The Salish Sea is a complex and dynamic estuarine ecosystem, within which Galiano Island is centrally situated (Fig. 1). Bounded to the north and south by two powerful tidal channels, the island sits at a confluence between tidal influx from the Pacific Ocean and freshwater runoff from the Fraser River, which combine to create a highly productive marine environment. A diverse array of marine habitats surround Galiano Island, many of which are dependent on foundation species (*sensu* Ellison 2019), defined as species which dominate an assemblage in number and size, determine the diversity of associated taxa through non-trophic interactions and modulate fluxes of nutrients and energy at multiple control points in the ecosystems they define. These habitats include bull kelp forests, eelgrass meadows, tidal salt marshes and deep-water glass sponge reefs (Fig. 2). Intertidal zones around Galiano Island are primarily rocky, comprising sandstones, conglomerates and shales of the Upper Cretaceous Nanaimo Group (Johnstone et al. 2006), with some shell, sand and mud beaches and brackish intertidal areas occurring at the mouth of seasonal and perennial creeks.

Galiano Island's coastal environment has a long history of habitation and cultural modification by Coast Salish Indigenous peoples, who intentionally enhanced the productivity of these rich marine ecosystems (Grier 2014, Grier et al. 2017). Notable cultural sites include Dionisio Point (Quelus), Retreat Cove (Xetthecum) and Montague (Sum'nuw' in the Hul’qumi’num language), the last of which includes a saltwater marsh and lagoon bordered by tombolo sand spits crested with shell midden and a possible intertidal fish trap (Grier et al. 2017, Fig. 2b). These salient aspects of this coastal landscape attest to its significance as a critical source of food for Coast Salish peoples who have lived in the region for more than 10,000 years (Carlson 2001). Evidence of Indigenous presence on Galiano Island, in particular, dates back more than 5,000 years (Grier et al. 2017). Marine animals of central importance to the Coast Salish diet include Pacific salmon (*Oncorhynchus* spp.), Pacific herring (*Clupeapallasii*), rockfish (*Sebastes* spp.) and halibut (*Hippoglossusstenolepis*), as well as a diversity of shellfish and marine mammals (Lepofsky and Caldwell 2013).

## Materials and methods

### Data curation

This dataset was curated through a systematic evaluation of taxonomic summaries and numerous catalogues of species occurrence data. We began by compiling unique taxa from source catalogues and resolving their taxonomy with reference to the World Register of Marine Species (WoRMS) (WoRMS Editorial Board 2021). This process resulted in a collection of classified summaries listing unique taxa by phylum. Each summary was subsequently reviewed by domain experts and revised as necessary. Where disagreements arose between taxonomic summaries and source catalogues, we revised reports and added critical annotations. Source catalogues were also thoroughly reviewed and records corrected as necessary. This iterative curation process was continued until a one-to-one correspondence was established between taxa represented in taxonomic summaries and source catalogues.

The data sources contributing to this baseline dataset have been normalised, aligned, corrected, synthesised and rendered into visualisations by a set of open source data processing scripts written in JavaScript. The algorithms developed to facilitate these workflows conserve memory of all modifications to the dataset, including changes to taxonomy, georeferencing and typographic errors. Georeferencing was reviewed and corrected with reference to the best available metadata using QGIS version 3.1.0 (QGIS Development Team 2021). Analysis of the relative contributions of each data source to our cumulative knowledge of species richness was implemented in R version 3.6.0 (R Core Team 2019). Taxon authorities, as supplied by experts, were validated through a semi-automated process referencing the WoRMS database (WoRMS Editorial Board 2021). All source data, processing scripts, documentation and outputs are tagged in GitHub. A protocol for reproducing these outputs can be found here.

### Data processing algorithms

The curation of this dataset was facilitated by algorithms that operate in the following steps:

1. The columns of each source catalogue, imported as CSV, are mapped with a common core of fields drawn from a subset of the Darwin Core standard (Wieczorek et al. 2012).
2. The taxon name is mapped with a core taxonomic backbone by means of a taxon resolution file which resolves preferred taxon names and accounts for typographical errors.
3. A dataset id is assigned to every source catalogue, which are then combined into a single master catalogue.
4. This catalogue is filtered to include only the taxa of interest—marine fauna.
5. Private or obscured coordinates held in project-specific fields are copied into the principal georeferencing fields.
6. A patch of georeferencing corrections is applied to the resulting coordinates, together with curatorial notes justifying the corrections.
7. The resulting observations are filtered by the polygon representing the project area.
8. The resulting output produces two consolidated CSV files: a catalogue of all observations and a master summary file.
9. The master summary file is then divided into phyla for curation by subject matter experts—these are exported into a Google Sheets representation where experts may edit them live.
10. Subject matter experts add curation notes and resolve taxonomic discrepancies, the results of which are checked against authorities listed in WoRMS (WoRMS Editorial Board 2021).
11. After curation, the Google Sheets are converted back into CSV, mirrored into the GitHub repository, re-integrated and compared with the original summary produced at Step 8.
12. Where appropriate, discrepancies between the summaries produced at Step 8 and Step 11 are circulated among the managers of the source catalogues to incorporate corrections. Residual discrepancies are then fed into the taxon resolution file at Step 2.
13. The process is re-run from Step 2 until repeated passes give rise to no further discrepancies at Step 12.

The files output at Step 8 of the pipeline form the basis of the map-based data visualisations referenced from this paper, as well as our Darwin Core data submission to the Global Biodiversity Information Facility (Simon and Basman 2021).

### Rationale

Our approach enhances common data cleaning practices by making minimal modifications to the source catalogues as they are incorporated into the dataset. Instead, source catalogues are retained in their original form and any transformations required by the data cleaning and normalisation process are encoded as a set of reversible patches or lenses applied to the data. In addition to supplying thorough documentation of our data-cleaning workflow, in line with best practices (Chapman 2005), we have also developed algorithms that can enact the same adjustments on a fresh incoming dataset. These workflows greatly aid reproducibility, reducing the time required to review the data after each processing step, as well as reducing the risk of errors being introduced through cut and paste and editing accidents.

For example:

- Taxonomic adjustments and spelling corrections pertaining to records in catalogues which we are unable to amend (e.g. Canadian Museum of Nature collections) are held in a taxon swaps file which is re-applied to the catalogues whenever they are updated. This process results in an institutional memory of the relationships between preferred taxonomies and those of contributors, while at once creating a log of commonly encountered typographical errors. Memory of such changes can be applied in the resolution of future datasets, helping to reduce redundancy in curatorial processes.
- Georeferencing corrections to historical records of poor spatial resolution are held in a georeferencing patch file, which may be re-applied to source catalogues when they are updated, without requiring the original source to accept our corrections.
- The iterative process described in Step 13 accommodates updates from ongoing observations and corrected upstream sources, as well as taxon-directed updates from subject matter experts, while ensuring that these updates do not introduce new errors into the system.

## Data resources

Scientific records of Galiano Island’s marine fauna date back over a century to specimens collected by Alexander Agassiz in 1859 (Agassiz 1862, McMurrich 1921) and Charles Newcombe in 1893 (RBCM 991-00066-001, 991-00066-002). Since then, the most extensive sampling efforts have been made through the Pacific Marine Life Surveys: systematic dives conducted by Charlie Gibbs, Donna Gibbs and Andy Lamb from 1967 to 2021. Additional sources incorporated into this dataset include: ecological data collected by remotely operated vehicle (ROV) (Chu and Leys 2010, Chu and Leys 2012); museum voucher specimens (Canadian Museum of Nature, Royal British Columbia Museum); reports from grey literature (Erickson 2000); and crowd-sourced observations from the Biodiversity Galiano Island project (iNaturalist) and the British Columbia Cetacean Sightings Network (https://imerss.github.io/imerss-bioinfo/Data%20Paper%20Part%20I%20Visualisation.html). A comprehensive catalogue of occurrence records is available on the Global Biodiversity Information Facility (Simon and Basman 2021). See supplementary materials for a formatted checklist of the marine animal diversity reported in this dataset (Suppl. material 1).

**Figure 3.**
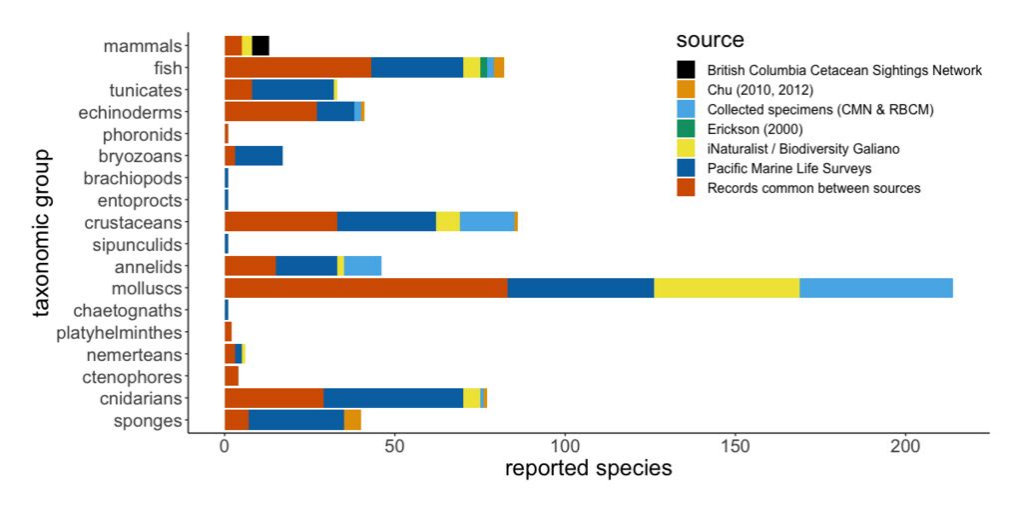
Bar plot summarising unique species reports by data source. Taxonomic overlap between catalogues includes 263 species reports that are common between sources. Note: voucher specimens representing two cnidarian species, cited by Agassiz (1862) and McMurrich (1921), could not be traced to a research collection, yet are here enumerated among 'collected specimens'.

The Pacific Marine Life Surveys represent the greatest proportion of data included in this study, with 16,150 species occurrence records accounting for 504 taxa (species, subspecies and species complexes), 241 of which are unique reports (Fig. 3). Collections at the Canadian Museum of Nature and the Royal British Columbia Museum include 272 voucher specimens representing 180 taxa and 77 unique reports. Records from Chu and Leys (2010) and Chu and Leys (2012) include 840 observations documenting 29 taxa, 11 of which are unique reports. A small dataset, including 12 salmonid observations by Erickson (2000), accounts for three taxa and two unique reports. Finally, observations on iNaturalist and the British Columbia Cetacean Sightings Network account for 2,748 occurrence records representing 316 taxa, 72 of which constitute novel reports. Taxonomic overlap between datasets includes 263 species reports that are common between sources. Museum specimens thus represent 19% and crowd-sourced citizen science observations 18%, of novel species reported for Galiano Island to date, with 3% of novel reports attributable to scientific and grey literature. The Pacific Marine Life Surveys account for the remaining 60% of novel species reports.

Table 1 provides an overview of the marine animal diversity reported for Galiano Island vis-à-vis estimates of global and regional biodiversity. The synopses which follow provide an overview of each taxonomic group in a format intended to be accessible to amateur naturalists interested in furthering research into the region's marine biodiversity. Note: certain phyla (incl. Acanthocephala, Echiura, Gastrotricha, Gnathostomulida, Hemichordata, Kinorhyncha, Loricifera, Mesozoa, Nematoda, Nematomorpha, Placozoa, Priapulida, Rotifera, Tardigrada and Vestimentifera), most of which are microscopic, are not represented in this dataset due to a lack of local data.

## Checklists

### Taxonomic summaries

#### Porifera

Grant, 1836

8E441C10-6C55-515E-8D60-8053667622AC

##### Notes

[**3 classes: 15 orders: 27 genera: 40 species**]

The name ‘Porifera’ derives from Modern Latin and means, literally, “bearing pores”, referring to the pores that perforate the body wall of these organisms.

Porifera are commonly known as sponges and have the simplest body plans of all multicellular animals, with no tissues or organs. Currently there are 9,452 valid species recognised worldwide, including representatives from both fresh and saltwater habitats (Hooper and van Soest 2002, Van Soest et al. 2012, de Voogd et al. 2021). Most sponges have a unique internal canal system used for circulating water, which is made of a series of progressively finer filters connected by chambers lined with flagellated cells called choanocytes. The phylum is divided into four classes: Calcarea (calcareous sponges), Demospongiae (demosponges), Hexactinellida (glass sponges) and the Homoscleromorpha (homoscleromorphs) (Gazave et al. 2011). Currently, there are 70 valid species recognised in the Puget Trough/Georgia Basin ecoregion (de Voogd et al. 2021), though many species remain undescribed in this cryptic group. Indeed, we estimate as many as 300 to 400 taxa may be present off the coast of British Columbia (Austin, unpubl. 2017). To date, 40 taxa have been documented locally, with all classes but the homoscleromophs confirmed for Galiano Island.

Most sponges are filter feeders that tend to thrive in strong current or wave-swept habitats. In tidal passages along the coast of British Columbia, sponges are often the most conspicuous and abundant sessile invertebrates present (Neil McDaniel, pers. obs.). Given that it is difficult to census the biodiversity found in areas with strong currents, additional species have likely gone undocumented from channels around Galiano Island. The homosceleromoph sponge *Oscarella* sp., for example, is known to occur in inlets of Vancouver Island and would likely be found in deeper waters around Galiano Island with further search effort. This dataset also includes three undescribed sponge species, which is likely a small percentage of the undescribed species in this region. Indeed, new sponge species are frequently described based on specimens sampled in deeper waters (Reiswig 2018).

**Figure 4.**
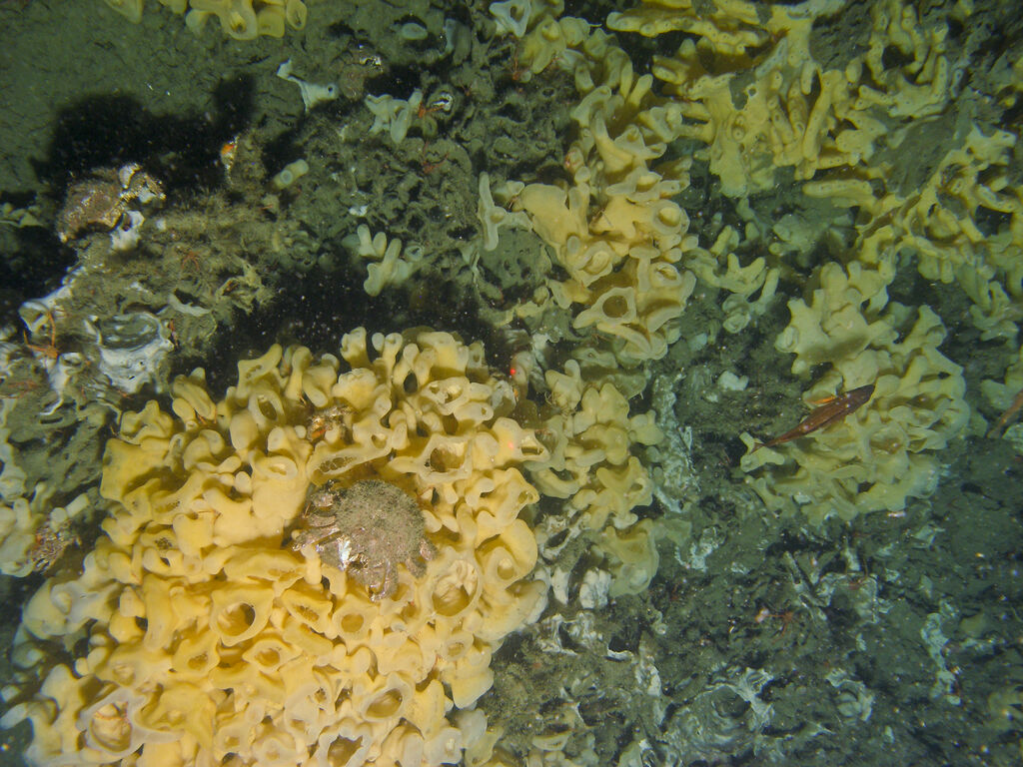
*Aphrocallistesvastus* (cloud sponge) – Photograph by Jackson Chu / Fisheries and Oceans Canada.

The glass sponges *Aphrocallistesvastus* Schulze, 1886 (cloud sponge) (Fig. 4) and *Heterochonecalyx* (Schulze, 1886) (fingered goblet sponge) form extensive deep-water reefs or bioherms, globally unique to the coasts of western Canada and the USA (Dunham et al. 2018). Glass sponge reefs contribute to the productivity of benthic ecosystems by forming habitat for diverse communities of invertebrates and fish and also play an important role in water filtration and carbon sequestration (Dunham et al. 2018). A total of 19 reef complexes have been mapped throughout the region, covering an area of ~ 12 km^2^ (Dunham et al. 2018). The reef off Galiano Island is one of the largest reefs mapped in the region, supporting dense populations of *A.vastus* and *H.calyx* and a high diversity of marine animal species (Chu and Leys 2010, Chu and Leys 2012).

Uncertainty regarding sponge diversity primarily results from their cryptic nature, which demands specialised knowledge of their skeletal structures and advanced microscopy techniques to confirm species. Most sponges are characterised by their calcareous or siliceous spicules. The abundance, morphology and arrangement of these spicules, along with the form, texture and colour of the sponge, are used to identify specimens with reference to previously-described species. Austin and Ott (Kozloff 1996) provide keys to 130 intertidal and shallow subtidal species, including ~ 20 calcareous sponges, eight glass sponges and 100 demosponges. Additional regional accounts of Porifera include Lamb and Hanby (2005), Carlton (2007), Harbo (2011), Austin et al. (2013), Austin et al. (2014), Jensen et al. (2018) and Ott et al. (2019). For annotated records of sponges reported for Galiano Island, see supplementary materials (Suppl. material 2).

#### Cnidaria

Hatschek, 1888

540D897E-1A07-5E27-84E6-5A1EC7150CAF

##### Notes

[**3 classes: 13 orders: 42 families: 64 genera: 77 species**]

‘Cnidaria’ is the Latinised plural form of the Greek κνίδη (knidē), which means “nettle.”

Cnidaria comprise 12,000 extant invertebrate species which primarily occur in marine environments (WoRMS Editorial Board 2021). The phylum is characterised by the possession of a cell called the cnidocyte, which is used for protection or prey capture. Cnidarians may be polypoid, medusoid or alternate between life stages and are grouped into seven classes: Anthozoa (anemones, soft corals, stony corals and sea fans), Cubozoa (box jellies), Hydrozoa (hydrozoans), Myxozoa (obligate parasites), Polypodiozoa (endocellular parasite), Scyphozoa (true jellyfish) and Staurozoa (stalked jellyfishes) (WoRMS Editorial Board 2021). Nearly 600 cnidarian species are known to occur along the Pacific coast of North America (Cairns et al. 2002), over 200 of which are reported for the coast of British Columbia (Brinckmann-Voss 1996, Brinckmann-Voss and Arai 1997, Sendall 2010, Boutillier and Gillespie 2011). Of these taxa, 77 species are reported in the Galiano Island record.

Cnidarians occur widely throughout the marine environment, with medusae present in pelagic and polyps in benthic habitats. While field identification is reliable in some cases, many taxa, especially hydrozoans, require microscopy for reliable determination. Owing to these limitations, certain groups, including anemones and corals, are well represented in the Galiano Island record, whereas others, such as hydroids and hydrocorals and the smaller scyphozoans, are not. The deeper-dwelling soft corals (gorgonians) are also under-represented due to sampling biases toward shallower waters.

**Figure 5.**
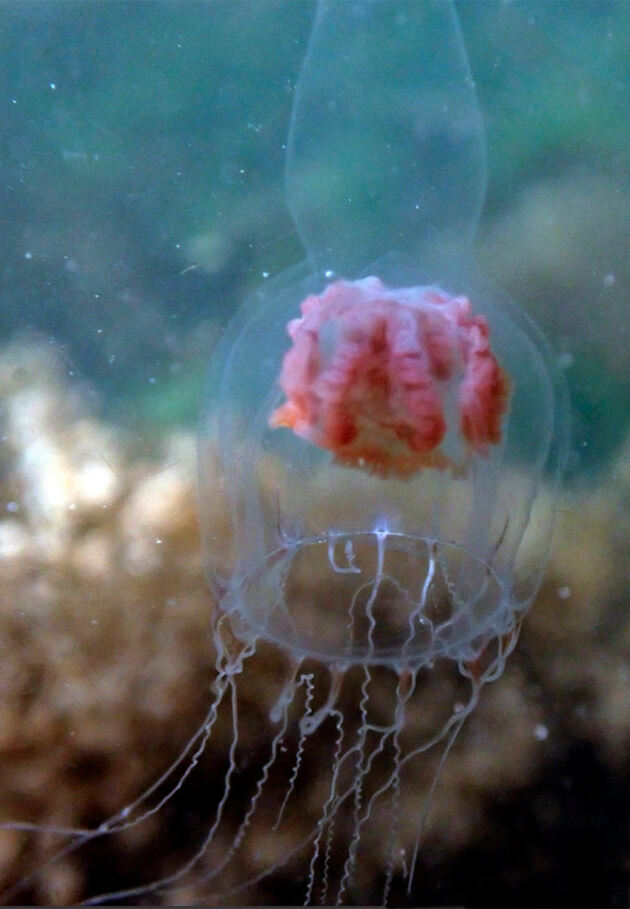
*Leuckartiaralongicalcar* – Photograph by Karolle Wall.

In this dataset, we report the recently-described hydroids *Leuckartiaralongicalcar* Schuchert, 2018 (Fig. 5) and *Similiclavanivea* Calder, Choong & McDaniel, 2015, the latter of which represents a novel family of athecate hydrozoan (Calder et al. 2015). Notable historical reports include the hydrozoan *Aglanthadigitale* (O. F. Müller, 1776) (pink helmit) and the non-native anemone *Diadumenelineata* (Verrill, 1869) (striped green sea anemone), both collected from Galiano Island in 1859 by Alexander Agassiz (Agassiz 1862, McMurrich 1921, Konecny and Harley 2019). Finally, reports of the anemone *Urticinagrebelnyi* Sanamyan & Sanamyan, 2006 (e.g. RBCM 986-00197-009) were found to be misidentified as *Urticinacrassicornis* (Müller, 1776) in our review of this dataset. *Urticinacrassicornis* is a European species concept commonly misapplied to *U.grebelnyi*, which is now recognised in the Northeast Pacific (Sanamyan and Sanamyan 2006).

Other reported taxa require further study to resolve their taxonomy. For instance, recent molecular analysis of *Aequorea* in the Northeast Pacific show they belong to a complex (here reported as *Aequoreavictoria* s. lat.), with many other taxa potentially included within this clade (Henry Choong and Wyatt Patry, pers. comm. 2020). Within *Alcyonium*, two taxa are recognised in the region, though they have yet to be circumscribed. *Euphysa* also likely represents a complex, the diversity of which has yet to be determined in our region (Claudia Mills, pers. comm. 2020).

The alternation between polypoid and medusoid generations has historically resulted in a great deal of confusion in the classification of cnidarians. Hydroids, in particular, have received little recent taxonomic attention, with the most substantive revisions dating to Fraser (1937). Modern revisions of hydroid taxonomy are currently underway, led by Dale Calder and Henry Choong. References for Northeast Pacific cnidarians are provided by Fraser (1937), Brinckmann-Voss (1996), Kozloff (1996), Brinckmann-Voss and Arai (1997), Cairns et al. (2002), Wrobel and Mills 2003, Lamb and Hanby (2005), Carlton (2007), Sendall (2010), Boutillier and Gillespie (2011), Harbo (2011), Choong 2015, Jensen et al. 2018 and Calder and Choong (2018). For annotated records of cnidarians reported for Galiano Island, see supplementary materials (Suppl. material 3).

#### Ctenophora

Eschscholtz, 1829

728E2FA5-0BF6-54D0-B0C3-B8B1355EB334

##### Notes

[**2 classes: 3 orders: 4 families: 4 genera: 4 species**]

‘Ctenophora’ means “comb-bearing”—deriving from the Greek κτείς (kteis), meaning "comb", and -φορος (-fóros), a suffix meaning "carrying”.

**Figure 6.**
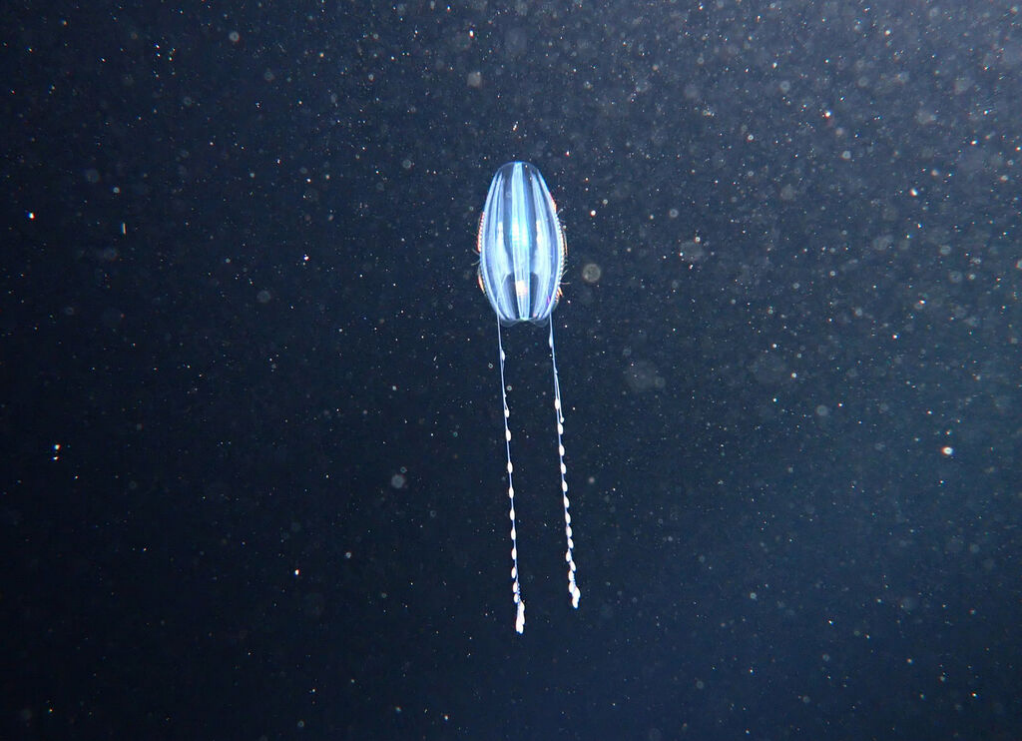
*Euplokamisdunlapae* (oval sea gooseberry) – Photograph by Kathleen Reed.

Ctenophora (comb jellies) are a phylum of predatory marine invertebrates that are characterised by having eight rows of ciliated plates for locomotion. The phylum is divided into two classes containing 150–200 extant species worldwide (Mills 2017). Around 32 ctenophore species are known to the Pacific coast of North America (Cairns et al. 2002), 13 of which are known within the Salish Sea—though only five or six of these species are relatively common (Claudia Mills, pers. comm. 2021). Four species are reported for Galiano Island, the least common of which is *Euplokamisdunlapae* Mills, 1987 (Fig. 6). This deep water species is abundant at 250 m depth, but only occasionally occurs above 100 m with mixing of the water column, especially during spring tides (Mackie et al. 1992).

Most ctenophores are pelagic species, with those most well known occurring near the ocean surface and those less understood found at greater depths. Ctenophores possess sticky cells called colloblasts to capture prey and are are highly diverse in morphology (e.g. egg-shaped cydippids, flat and generally combless platyctenids and large-mouthed beroids). The phylogenetic position of Ctenophora in relation to other phyla is still debated and their taxonomy is in dire need of revision (Dunn et al. 2015, Mills 2017). References for Northeast Pacific ctenophores include Kozloff (1996), Wrobel and Mills (2003), Lamb and Hanby (2005), Carlton (2007), Baldwin (2009d), Harbo (2011) and Jensen et al. (2018). For annotated records of ctenophores reported for Galiano Island, see supplementary materials (Suppl. material 4).

#### Nemertea

Schultze, 1851

5B4284B4-AEF5-5F8D-B1B7-B395BC374B3B

##### Notes

[**3 classes: 3 orders: 4 families: 4 genera: 6 species**]

‘Nemertea’ is the Modern Latin (plural) form of the Greek νημερτής (Nēmertēs)—the name of a sea nymph.

Nemertea, also known as ribbon worms, are a phylum of soft-bodied and unsegmented invertebrate animals. Over 1,300 extant species, mostly found in marine environments, have been described globally (WoRMS Editorial Board 2021). Traditionally separated into two classes, four classes are now recognised: the Arhynchocoela, Hoplonemertea, Palaeonemertea and Pilidiophora (Strand et al. 2018). The Salish Sea is home to 30 of the 41 nemertean species known to the coast of British Columbia (Baldwin 2010a, GBIF.org 2021). Six of these taxa have been documented for Galiano Island to date.

Most nemerteans live in benthic environments, lurking in crevices beneath stones, shells and at the bases of algae or sessile animals. They are largely predatory or scavenging animals, feeding on annelids, clams and crustaceans, though some are commensalists that live within the mantle cavity of molluscs where they feed on micro-organisms filtered out by their hosts (Ruppert et al. 2004). Sampling nemerteans intertidally is most easily accomplished by flipping stones on the beach or by careful observation at very low tides when they are most likely to be exposed.

**Figure 7.**
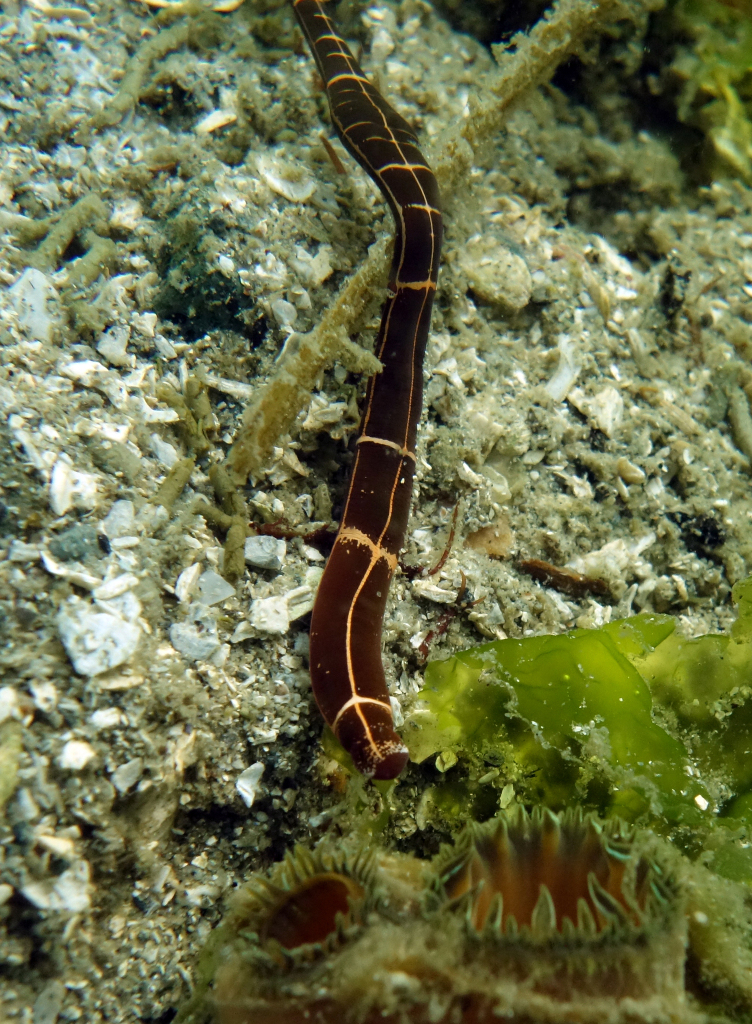
*Tubulanussexlineatus* (six-lined ribbon worm) – Photograph by Karolle Wall.

The key diagnostic features of Nemertea are proboscis characteristics: for example, whether it is armed or unarmed or split into regions. This makes identification of many species difficult as it requires examination of internal anatomy. Some species, such as *Tubulanussexlineatus* (Coe, 1904) (six-lined ribbon worm) (Fig. 7), have distinct colour patterns and can be readily identified in the field. Otherwise, a more complete knowledge of Galiano Island's nemerteans would rely on specialised taxonomic expertise, with a focus on benthic communities. Regional references for Nemertea include: Coe (1901), Coe (1904), Coe (1905), Coe (1944), Kozloff (1996), Lamb and Hanby (2005), Carlton (2007), Baldwin (2010a), Harbo (2011) and Jensen et al. (2018). For annotated records of nemerteans reported for Galiano Island, see supplementary materials (Suppl. material 5).

#### Platyhelminthes

Minot, 1876

B5C82C02-55DB-54C1-8C04-3FFE296ADC7B

##### Notes

[**1 class: 1 order: 2 families: 2 genera: 2 species**]

‘Platyhelminthes’ is rooted in the Greek πλατύ (platy), meaning “flat”, and ἑλμινθ- (helminth-), meaning “worm”.

Platyhelminthes (flatworms) are a phylum of dorsoventrally flattened, bilaterally symmetrical invertebrates, most of which are found in marine and freshwater environments (Collins 2017). The phylum includes nearly 13,000 extant marine species (WoRMS Editorial Board 2021), though this estimate does not account for cryptic parasitic species, such as trematodes or flukes, and many taxa remain undescribed. Over 170 species are reported for the coast of British Columbia (Baldwin 2010b), two of which have been reported for Galiano Island to date.

**Figure 8.**
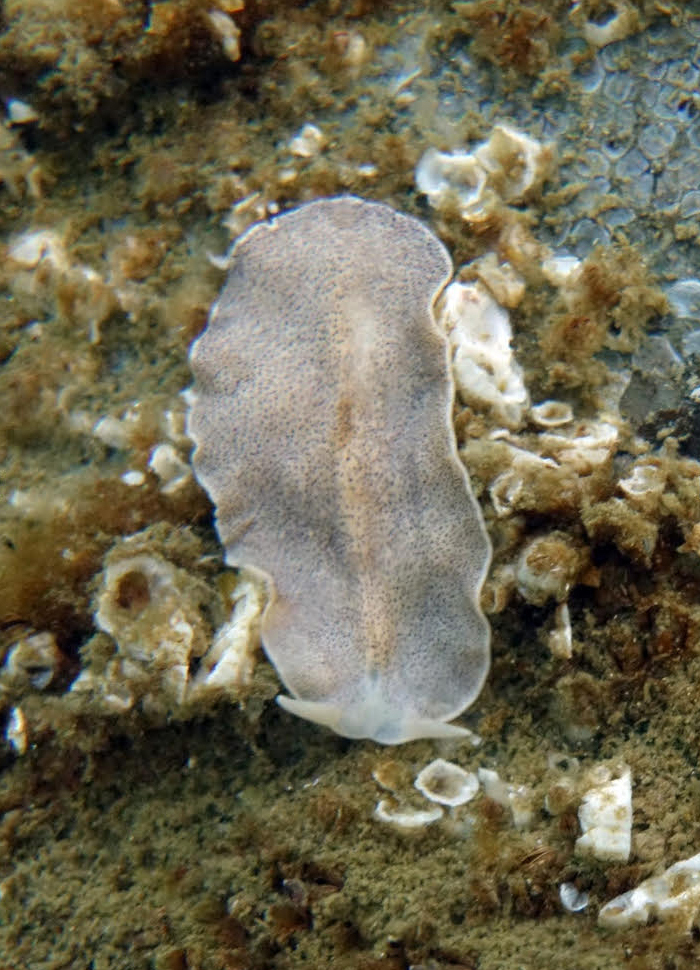
*Euryleptaleoparda* (spotted flatworm) – Photograph by Karolle Wall.

Given the region's high diversity of cryptic free-living and parasitic flatworm species, expert search effort is necessary to gain a comprehensive understanding of this understudied phylum. The larger flatworm species in the order Polycladia are the most likely to be found diving or in intertidal areas. However, even with this more visible group, species identification can be difficult, requiring the fixing, cleaning and sectioning of specimens to examine the microscopic details of copulatory organs and other internal anatomy. Only the largest and most distinctly patterned taxa, such as *Euryleptaleoparda* Freeman, 1933 (spotted flatworm) (Fig. 8), can easily be identified in the field. Of these more conspicuous taxa, around 10 species are likely to be encountered diving around Galiano Island. Many more species could potentially be documented by taxonomic specialists via searches targeting the more obscure free-living species or fluke, tapeworm and monogenean parasites. Regional taxonomic references for platyhelminthes include Kozloff (1996), Lamb and Hanby (2005), Carlton (2007), Baldwin (2010b) and Jensen et al. (2018). For annotated records of platyhelminthes reported for Galiano Island, see supplementary materials (Suppl. material 6).

#### Chaetognatha

Leuckart, 1854

3C3B08E8-A6FA-59B1-A596-3EAA6D0F0F75

##### Notes

[**1 class**]

The name ‘Chaetognatha’ derives from the Greek χαίτη (khaítē), meaning “bristle”, and γνάθος (gnáthos), meaning “jaw”.

**Figure 9.**
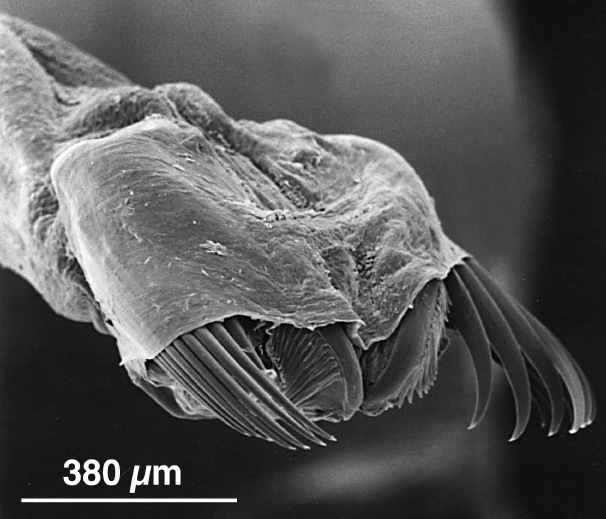
*Parasagittaelegans* (arrow worm) – scanning electron miscroscope micrograph by Elaine Humphrey.

Chaetognatha (arrow worms) are a small phylum of predatory animals, all of which are marine, including 130 extant species in the class Sagittoidea (WoRMS Editorial Board 2021). Though they are not diverse, arrow worms are extremely numerous, representing a major component of marine plankton worldwide. Of the six species of arrow worm known to the Northeast Pacific, four have been sampled in the waters off the coast of British Columbia (Lea 1955, Gardner 1982). *Parasagittaelegans* (Verrill, 1873) (Fig. 9) is the most abundant species reported from the sheltered inland waters of the Salish Sea (Lea 1955). *Eukrohniahamata* (Möbius, 1875) is also known to occur infrequently in inland channels, as an oceanic species that is occasionally borne in by the intrusion of offshore waters (Lea 1955, Gardner 1982). It is not possible to determine chaetognath reports in the Galiano Island record as they are based on dive observations, but studies indicate that, in all likelihood, these observations refer to *P.elegans*. Sampling by use of plankton tows and microscopic analysis would help confirm the local fauna.

Chaetognaths are active predators, grasping their prey with two sets of rigid hooks located at the sides of the head, hence the common name “bristle-mouth.” These predatory animals are chiefly pelagic, occurring in the open ocean. Appearing like a glass arrow in the water, chaetognaths swim by flexing in the middle of the body, which is supported by a hydrostatic skeleton. The lateral fins on either side of the body appear to aid buoyancy, but are not involved in movement. As few species are present locally, students may disregard more cryptic characteristics and distinguish species by focusing on the arrangement of lateral fins and eye colouration (Lea 1955). For a treatment of British Columbia's chaetognaths, including a set of technical keys, see Wailes (1929), Lea (1955), Bieri (1959), Gardner (1982), Kozloff (1996), Lamb and Hanby (2005) and Carlton (2007) provide additional accounts. For annotated records of chaetognaths reported for Galiano Island, see supplementary materials (Suppl. material 7).

#### Mollusca

Linnaeus, 1758

CC76F9FF-7BAF-5982-90E9-F415D4BDE467

##### Notes

[**5 classes: 31 orders: 101 families: 151 genera: 214 species**]

'Mollusca' is rooted in the Modern Latin 'mollusca', the neuter plural form of 'molluscus', from 'mollis', meaning “soft”.

With about 49,000 extant species (WoRMS Editorial Board 2021), Mollusca are the second largest phylum after Arthropoda (Rosenberg 2014). The phylum comprises seven extant classes (Haszprunar 2001), five of which are locally represented, including Bivalvia (bivalves), Cephalopoda (cephalopods), Gastropoda (snails and slugs), Polyplacophora (chitons) and Scaphopoda (tusk shells). Over 780 marine species are been identified in British Columbia (Bernard 1967, Baldwin 2009b, Cosgrove 2009, Millen 2012, Forsyth and Harbo 2013, Clark 2020), 214 of which are reported for Galiano Island.

**Figure 10.**
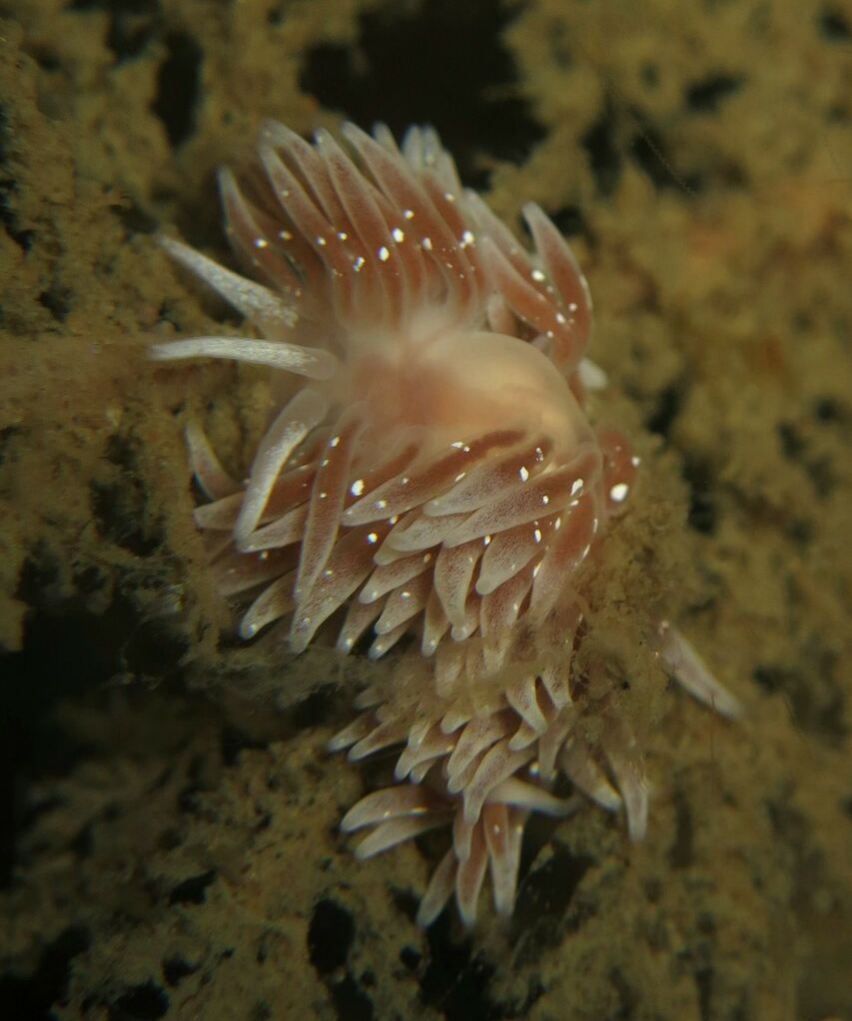
*Zelentianepunicea* (pimpled aeolid) – Photograph by Karin Fletcher.

Mollusc inventories have historically been limited by outdated taxonomy and other challenges, leaving much hidden diversity that remains to be discovered in the region. As with other groups, European species concepts have historically been misapplied to Northeast Pacific taxa among Mollusca. One example is the aeolid nudibranch *Cuthonapustulata* (Alder & Hancock, 1854), originally described from the Northeast Atlantic in 1854. Specimens collected under this name from Porlier Pass off Galiano Island by Sandra Millen in 1982 (RBCM 983-00026-001) have since been described as *Zelentianepunicea* Korshunova, Fletcher, Lundin, Picton & Martynov, 2018 (pimpled aeolid, Fig. 10), based on genetic sequencing results (Korshunova et al. 2018). Other novel nudibranch species have also been circumscribed in part based on specimens collected from local waters. One species complex, which was reported as *Cadlinaluteomarginata* MacFarland, 1966 (yellow-rimmed nudibranch) for over 50 years, has since been split into four different species, including *C.klasmalmbergi* (Klas' yellow-rimmed nudibranch), first collected from Baines Bay on Galiano Island (Korshunova et al. 2020).

While many nudibranch species revealed through genetic methods can be determined based on morphology, other cryptic taxa often cannot be reliably identified by morphology alone, requiring molecular analysis for confirmation. Locally occurring genera currently under taxonomic revision include *Crepidula* Lamarck, 1799 and *Vermetus* Daudin, 1800. Many more taxonomic issues remain to be resolved in future studies.

The cold, rich waters surrounding Galiano Island are home to many remarkable molluscs, including *Enteroctopusdofleini* (Wôlker, 1910) (giant Pacific octopus), the largest octopus in the world. The largest chiton in the world, *Cryptochitonstelleri* (von Middendorff, 1847) (gumboot chiton), is also known to the region and is reported for Galiano Island. Introduced bivalves present around Galiano Island include *Magallanagigas* (Thunberg, 1793) (Pacific oyster), *Myaarenaria* Linnaeus, 1758 (soft-shelled clam), *Nuttaliaobscurata* (Reeve, 1857) (purple mahogany clam) and *Ruditapesphilippinarum* (Adams & Reeve, 1850) (Japanese littleneck), all of which are now prolific in the Northeast Pacific. Introduced gastropods present include *Batillariaattramentaria* (G.B. Sowerby II, 1855) (Japanese false cerith) and *Myosotellamyosotis* (Draparnaud, 1801) (mouse-eared snail).

Species that will likely be uncovered with further search effort include the threatened native *Ostrealurida* (Carpenter, 1864) (Olympia oyster), the endangered *Haliotiskamchatkana* (Jonas, 1845) (northern abalone) and *Penitellapenita* (Conrad, 1837) (flat-tip piddock). Numerous families of minuscule “micro-molluscs” are entirely unrepresented in the Galiano Island record. A diverse chiton fauna may also be under-represented locally, with 39 species in six families known from the intertidal zone to moderate diving depths (< 30 m) in the Salish Sea (Clark 2020), about two thirds of which (27 spp.) have been documented around Galiano Island to date.

Molluscs were traditionally identified based on shell and other morphological characters. However, modern taxonomic treatments generally rely on genetics in addition to morphology, habitat and host species. Many snails also lay diagnostic egg cases. Regional references for Mollusca include: Bernard (1967), Abbott (1974), Kozloff (1996), Harbo (1997), Harbo et al. (1997), Lamb and Hanby (2005), Carlton (2007), Harbo (2011), and Jensen et al. (2018). Bivalves are treated by Foster (1991), Coan et al. (2000), Huber (2010) and Forsyth and Harbo (2013); cephalopods by Cosgrove (2009) and Cosgrove and McDaniel (2009); chitons by Baldwin (2009b) and Clark (2020); gastropods by Behrens (2004), Behrens and Hermosillo (2005), Millen (2012), Korshunova et al. (2018) and Korshunova et al. (2020); and tusk shells by Carlton (2007). For annotated records of molluscs reported for Galiano Island, see supplementary materials (Suppl. material 8).

#### Annelida

Lamarck, 1802

29F4CE4D-9AD9-5540-BA0B-E477A1EFA1E9

##### Notes

[**2 classes: 6 orders: 15 families: 39 genera: 46 species**]

‘Annelida’ was coined in Modern Latin by the French naturalist Jean-Baptiste Lamarck from the French 'annélide', deriving from 'annelés', meaning “ringed ones” (from the Latin 'anulus', for “little ring”).

Annelida are a large phylum of invertebrates, commonly referred to as ringed or segmented worms, comprising over 13,000 extant species (WoRMS Editorial Board 2021). The majority of known species are found in marine environments, though terrestrial and freshwater annelid diversity is likely underestimated—as is the number of marine species. The phylum is traditionally divided into two classes: Clitellata (earth worms, leeches and brachiobdellids) and Polychaeta (polychaetes) (Ruppert et al. 2004). Based on molecular evidence, Sipuncula (peanut worms) and Echiura (spoon worms) are also nested within this clade (Struck et al. 2007). As the taxonomy has not yet been reorganised, however, Sipuncula reported for Galiano Island are here treated as a separate phylum.

Over 450 polychaete species are reported by Baldwin (2009c) for coastal British Columbia. However, many of these species have been identified based on European concepts now known to represent complexes of morphologically similar but genetically different organisms, including many newly-recognised outside of Europe (Carr et al. 2011). In the greater Salish Sea bioregion, no less than 860 annelid species are currently reported from intertidal areas through shelf depths (Leslie Harris, unpublished data). Once deep water and cryptic species are considered, however, the total number of annelids expected to occur in our waters will likely double. Of these, 46 species are reported to date from Galiano Island.

Annelid life history is exceedingly diverse, exhibiting virtually all feeding modes, including suspension feeding, deposit feeding, scavenging, herbivory, and carnivory. This diversity is mirrored in their pervasive distribution throughout most benthic and pelagic marine environments. Gaps in our knowledge of Galiano Island’s annelid diversity partly reflect the challenges that have historically limited our broader understanding of the region’s diversity. Taxonomists working in the region in the past largely relied on European references and only assigned new names for taxa with strikingly different features. However, the characters defining organisms within this challenging group are often very small and difficult to ascertain without resorting to dissection or microscopic examination. Furthermore, the vast majority of polychaetes lie hidden in bottom sediment or among sessile organisms and are rarely noticed by casual observers. Specialised equipment, such as benthic grabs, dredges, sediment air lifts, plankton tows and settlement plates, are needed to capture more than the few conspicuous species seen by beach-goers and divers.

**Figure 11.**
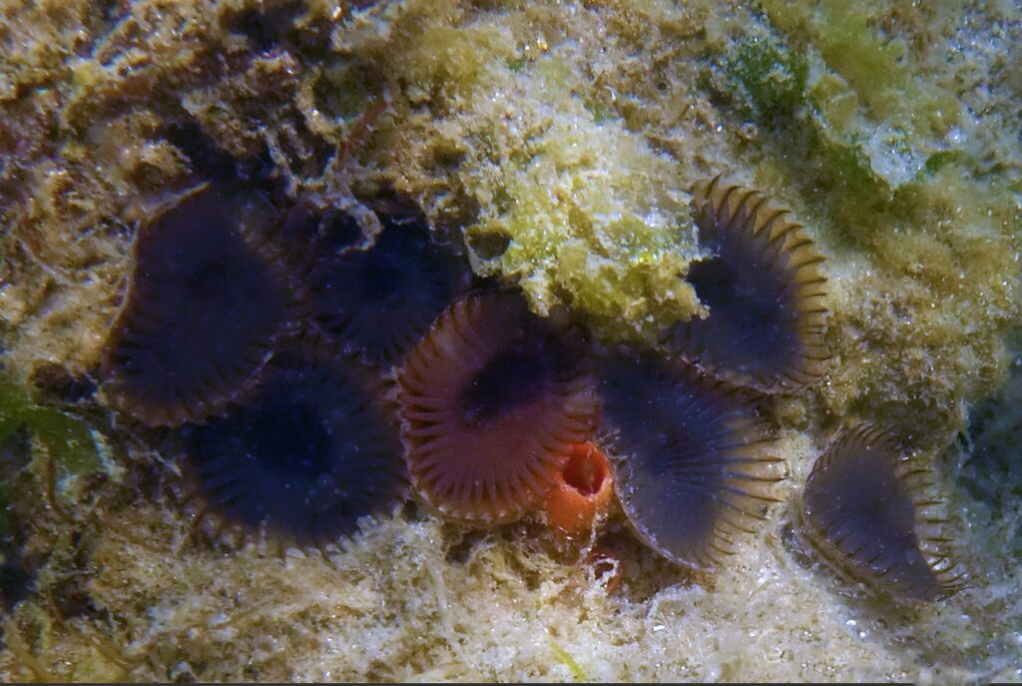
Myxicolaaff.aesthetica (*Myxicola* sp. A, sensu Leslie Harris, provisional name) – Photograph by Karolle Wall.

The majority of taxa reported in this dataset are polychaetes, though a few clitellates are also represented. Many of the names reported reflect the ongoing challenges facing those studying this group. Uncertainty is indicated by older reports, such as *Chaetopterusvariopedatus* (Renier, 1804) and *Nereiszonata* Malmgren, 1867 (here reported as the *Chaetopterusvariopedatus* complex and Nereiscfzonata), which refer to European concepts in which undescribed native species likely remain hidden. Even conspicuous species, such as tubeworms in the genus *Myxicola* (Fig. 11), commonly reported as *M.aesthetica* or *M.infundibulum* (here listed as Myxicolaaff.aesthetica and Myxicolaaff.infundibulum), are currently under revision. Many other specimens catalogued in this dataset are not determined past genus as specimens were either unavailable or require further examination in light of recent taxonomic revisions.

Polychaete morphology is highly variable, yet their body plan generally consists of an elongated and segmented body. It is the differentiation of their body into segments and the appendages (gills, parapodia, chaetae, cirri, palps etc.) attached to these segments that enables the morphological classification of families, genera and species. Regional references treating annelids include Berkeley and Berkeley (1948), Berkeley and Berkeley (1952), Berkeley and Berkeley (1962), Berkeley (1967), Berkeley (1968), Banse (1979), Banse (1981), Hobson and Banse 1981, Saphronova (1991), Blake (1994), Blake (1995), Hilbig (1995a), Hilbig (1995b), Kudenov and Harris (1995), Blake (1996a), Blake (1996b), Blake (1996c), Blake (1996d), Blake (1996e), Kozloff (1996), Blake (1997), Hilbig (1997a), Hilbig (1997b), Hilbig (1997c), Blake (2000a), Blake (2000b), Blake (2000c), Blake (2000d), Blake (2000e), Hilbig (2000a), Hilbig (2000b), Hilbig (2000c), Lamb and Hanby (2005), Carlton (2007), Baldwin (2009c), Harbo (2011) and Jensen et al. (2018). For annotated records of annelids reported for Galiano Island, see supplementary materials (Suppl. material 9).

#### Sipuncula

Stephen, 1964

6D0CB784-6C96-51CE-A239-2C4C3FD7E187

##### Notes

[**1 class: 1 order: 1 family: 1 genus: 1 species**]

‘Sipuncula’ is the Modern Latin plural form of the genus *Sipunculus*, based on the Latin 'siphunculus', meaning “small tube.”

**Figure 12.**
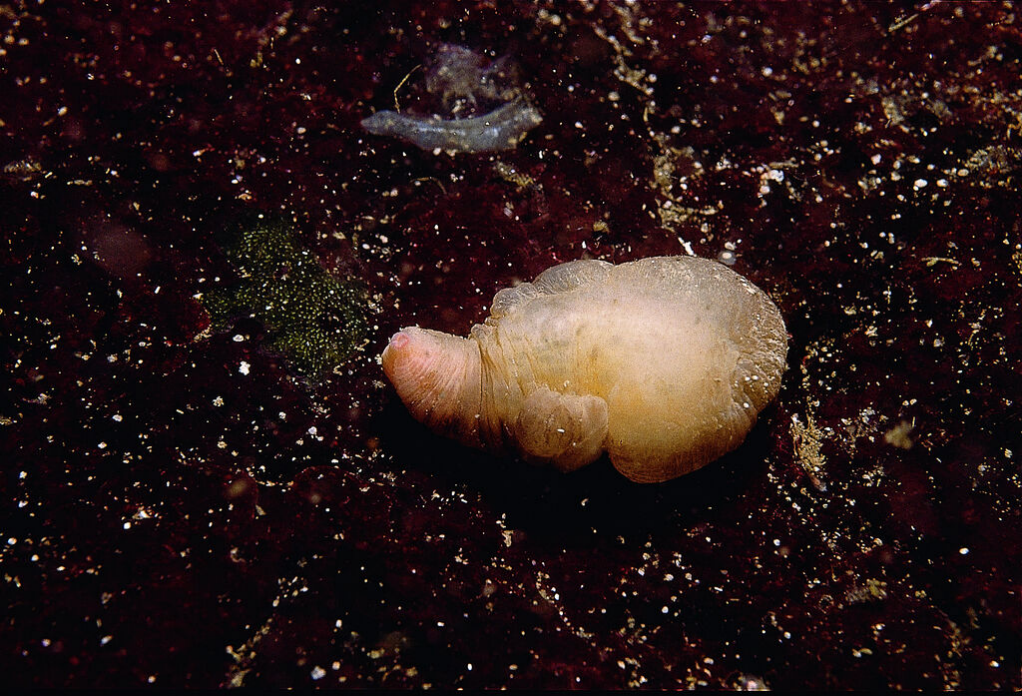
*Golfingiavulgaris* (brown peanut worm) – Photograph by Bernard P. Hanby.

Sipuncula (peanut worms) are a small phylum of bilaterally symmetrical, unsegmented marine worms, represented by 150 species worldwide (WoRMS Editorial Board 2021)—though a certain amount of cryptic diversity is understood to remain hidden in the phylum (Hsu et al. 2013). Based on molecular evidence, Sipuncula are now considered to be nested within the phylum Annelida along with Echiura (spoon worms) (Boyle and Rice 2014), yet are here treated as a separate phylum. Eight species of Sipuncula are known to the Northeast Pacific (Carlton 2007), of which one, Golfingia (Golfingia) vulgaris vulgaris (de Blainville, 1827) (Fig. 12), is reported for Galiano Island.

Due to their burrowing habit, sipunculids are obscure organisms easily overlooked by divers and casual beachcombers. Some Sipuncula inhabit rock crevices or similar protected areas, while others are found in sand or mud. With additional search effort, other taxa likely to be uncovered in the waters around Galiano Island include Phascolosoma (Phascolosoma) agassizii Keferstein, 1866 and Themiste (Themiste) pyroides (Chamberlin, 1919). Identification relies on features of the tentacles which tend to be drawn into the body when contracted, hence the common name “peanut worms.” Regional references to the sipunculids can be found in Kozloff (1996), Lamb and Hanby (2005) and Carlton (2007). For annotated records of sipunculids reported for Galiano Island, see supplementary materials (Suppl. material 10).

#### Crustacea

Brünnich, 1772

35C84A81-37B6-5470-8179-1A00185E2BFE

##### Notes

[**3 classes: 7 orders: 32 families: 52 genera: 86 species**]

The name ‘Crustacea’ is rooted in the Modern Latin 'crusta', meaning “shell.”

Crustacea are a highly diverse subphylum of Arthropoda that contains over 52,000 described terrestrial, marine and freshwater species (Martin and Davis 2001). This subphylum includes six classes (Martin and Davis 2001), five of which are represented locally: Branchiopoda (fairy shrimp and water fleas); Hexanauplia (barnacles, copepods and tantulocarids); Ichthyostraca (fish lice); Malacostraca (amphipods, crabs, isopods, krill and shrimp); and Ostracoda (seed shrimp). Over 900 marine crustacean species are known to occur in British Columbia (Hart 1931, Cornwall 1970, Gardner and Szabo 1982, Daly and Holmquist 1986, Funk and Hobson 1991, Martin et al. 1996, del Carmen Espinosa-Pérez and Hendrickx 2006, Carlton 2007, Baldwin 2009a, Baldwin 2011, Baldwin 2016, Sieg 2017, Green and Gardiner 2019), 86 of which are reported for Galiano Island.

Many crustaceans are difficult to identify without microscopy, contributing to biases in the local record. Locally, ichthyostracan crustaceans are vastly under-represented and cladocerans and ostracods remain unrepresented to date. As of yet, there are no records of hoplocarids (mantis shrimps), ascothoracids or tantulocarids occurring within British Columbia. The latter two groups will likely be discovered with further investigation; hoplocarids, however, have never been observed north of southern California and are unlikely to occur in the region. Leptostracans are not well described, though at least one species from the genus *Nebalia* is known to the Northeast Pacific (Martin et al. 1996). Closer examination of subtidal and intertidal eelgrass, algae wrack and the rocky intertidal zone will uncover many unrecorded taxa.

**Figure 13.**
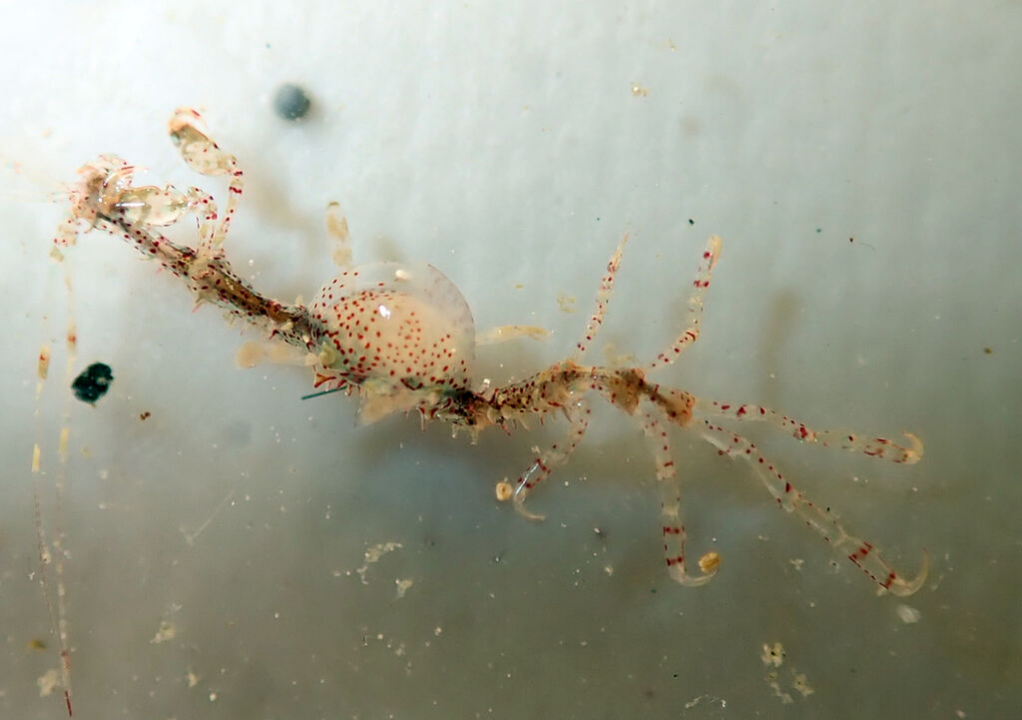
*Caprellamutica* (Japanese skeleton shrimp) – Photograph by Emily Adamczyk.

Numerous non-native crustaceans are known to the region, though only one introduced species is currently represented in the Galiano Island record. The introduced *Caprellamutica* Schurin, 1935 (Japanese skeleton shrimp) (Fig. 13), reported for Galiano Island, is now well established along the coast of British Columbia (Frey et al. 2009). The invasive *Carcinusmaenas* (Linnaeus, 1758) (European green crab) and two tanaids, *Leptocheliadubia* (Krøyer, 1842) and *Zuexonormani* (Richardson, 1905), have been found off Vancouver Island, but are not yet known locally. Additionally, the non-native amphipods *Ampithoevalida* Smith, 1873, *Caprelladrepanochir* Mayer, 1890, *Incisocalliopederzhavini* (Gurjanova, 1938), *Melita nitida* S.I. Smith in Verrill, 1873, *Monocorophiumacherusicum* (Costa, 1853), *Monocorophiuminsidiosum* (Crawford, 1937) and *Jassaslatteryi* Conlan, 1990, along with the bay barnacle *Amphibalanusimprovisus* (Darwin, 1854), have all been reported in the Strait of Georgia (Lu et al. 2007, Gartner et al. 2016), but have not yet been reported for Galiano Island.

References for regionally-occurring marine crustaceans include: amphipods (Baldwin 2009a); barnacles (Cornwall 1970, Baldwin 2016); cladocerans (Green and Gardiner 2019); cumaceans (Hart 1931); decapods (Baldwin 2011, Jensen 2014); copepods (Gardner and Szabo 1982); isopods (del Carmen Espinosa-Pérez and Hendrickx 2006); mysids (Daly and Holmquist 1986); tanaids (Sieg 2017); and krill (Funk and Hobson 1991, Li et al. 2013). General references to marine crustaceans known to the region can also be found in Kozloff (1996), Lamb and Hanby (2005), Carlton (2007), Harbo (2011) and Jensen et al. (2018). For annotated records of crustaceans reported for Galiano Island, see supplementary materials (Suppl. material 11).

#### Entoprocta

Nitsche, 1869

886C1CF8-A729-52E7-A8B1-5DC23FEC676A

##### Notes

[**1 class: 1 order: 1 family: 1 genus: 1 species**]

‘Entoprocta’ derives from the Greek ἐντός (entos), meaning “inside”, and πρωκτός (prōktos), meaning “anus.”

**Figure 14.**
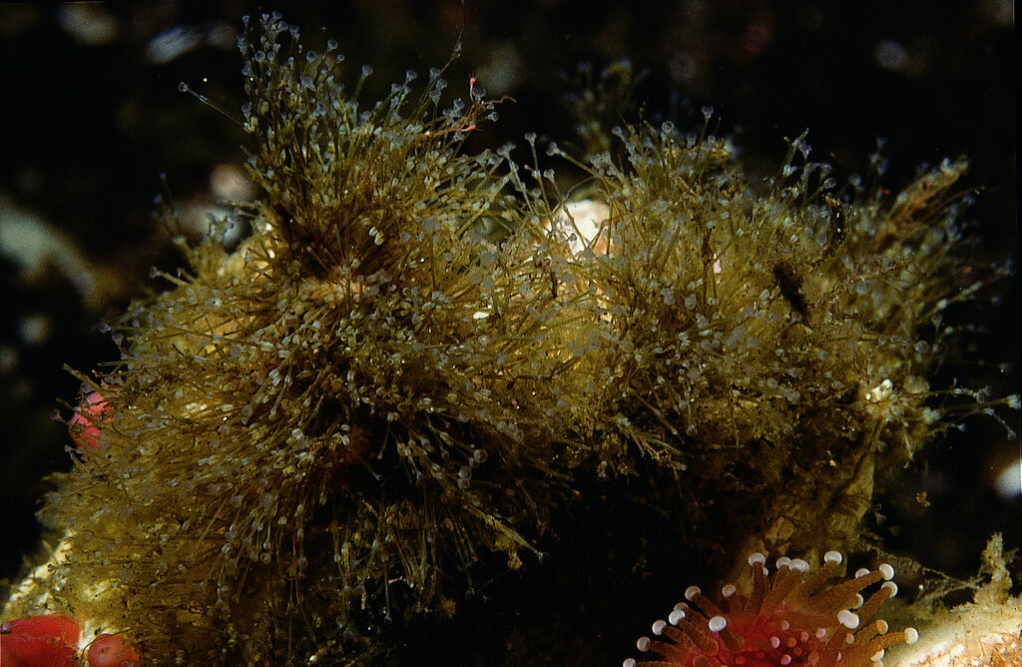
*Barentsia* sp. (nodding heads) – Photograph by Bernard P. Hanby.

Entoprocta (nodding-heads) are a small phylum of mainly sessile marine invertebrates that comprises 253 described species worldwide (WoRMS Editorial Board 2021). The phylum was traditionally divided into two orders: the Solitaria and Coloniales. Solitaria included solitary species that usually attach to larger organisms which produce feeding currents, such as sponges, bryozoans, polychaetes, sipunculans and ascidians, and are typically associated with just one or a few host species. Coloniales, by contrast, included colonial species that form crusts on various surfaces. However, the traditional system is no longer followed; instead four families are now recognised (WoRMS Editorial Board 2021). Of the 11 species known to British Columbia (Baldwin 2009e), one species is reported for Galiano Island: *Barentsia* sp. (Hincks, 1880) (Fig. 14).

Entoprocts are filter feeders, trapping small particles in the mucus secreted by their tentacles. An understudied group, they are easily overlooked because of their diminutive size (< 1 mm) and the superficial similarities they share with the Cnidaria and Bryozoa (Ruppert et al. 2004). With further search effort, others are likely to be detected locally.

The body plan of entoprocts consists of a cup-like calyx that bears a ring of ciliated tentacles called ­the lophophore, which is attached on its dorsal surface to the substrate by a long, thin stalk or pedicel. Entoprocta have their anus inside their ring of tentacles, while bryozoans (formally known as the Ectoprocta), have their anus outside of their ring of tentacles. Regionally occurring Entoprocta are treated by Kozloff (1996), Lamb and Hanby (2005), Carlton (2007) and Baldwin (2009e). For annotated records of entroprocts reported for Galiano Island, see supplementary materials (Suppl. material 12).

#### Brachiopoda

Duméril, 1805

8F9A36E3-DAC6-5609-B045-5ED138645797

##### Notes

[**1 class: 1 order: 1 family: 1 genus: 1 species**]

‘Brachiopoda” is formed of the Ancient Greek βραχίων (brakhíōn), meaning “arm”, and πούς (poús), for “foot”.

**Figure 15.**
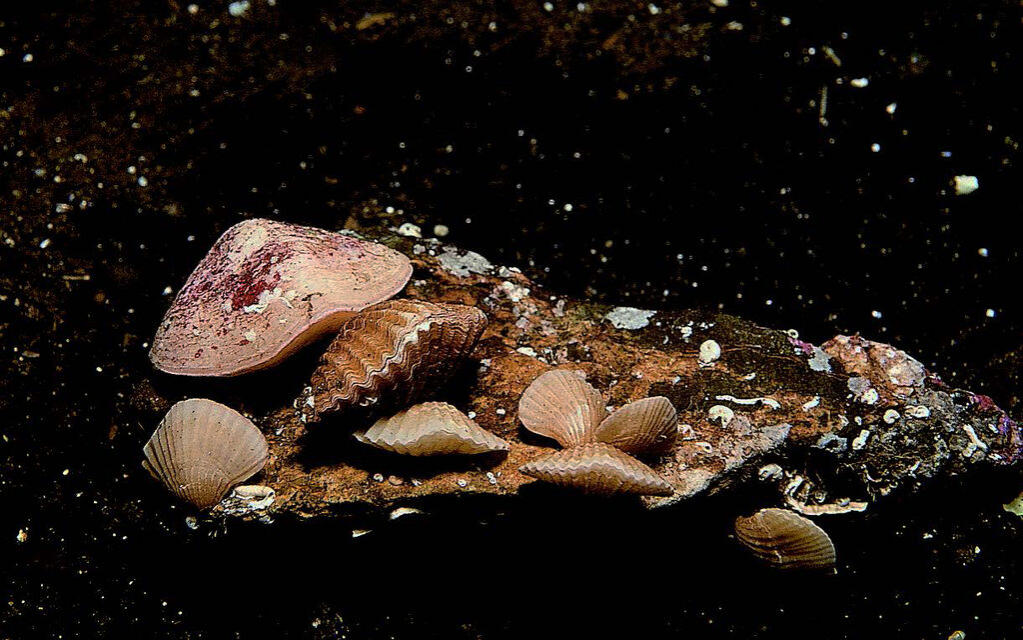
*Terebrataliatransversa* (transverse lamp shell) – Photograph by Bernard P. Hanby

Brachiopods (lampshells) are a phylum of shelled marine animals found in intertidal and subtidal waters, represented by over 400 species worldwide (WoRMS Editorial Board 2021). The phylum may be divided into three extant subphyla, including the Craniiformea, Linguliformea and Rhynchonelliformea (WoRMS Editorial Board 2021). Of the seven lampshell species known to British Columbia, only three are expected to occur within the Salish Sea: *Laqueuscalifornicus* (Koch, 1848), *Terebrataliatransversa* (Sowerby, 1846) and *Terebratulinaunguicula* (Carpenter, 1864). One species, *T.transversa* (Fig. 15), is known to Galiano Island.

Most lampshells live at great depths and are thus seldom encountered. *Laqueuscalifornicus* is known to occur in mass aggregations (945 m^-2^) at depths as deep as 700 m, which is likely why this species has eluded search efforts to date (Tunnicliffe and Wilson 1988). *Terebratulinaunguicula* occurs within the subtidal range sampled; however, this species is notable for inhabiting the extremely low-oxygen waters occurring in some fjords within British Columbia and, thus, may not be expected to occur locally (Tunnicliffe and Wilson 1988).

Traditionally, brachiopods were classified into two major groups, based on whether their valve hinge structures are articulate or inarticulate. Shells are orientated in a dorsal-ventral manner, closely resembling bivalve molluscs, the shells of which are lateral in orientation. Yet, whereas bivalves are symmetrical between valves (valves mirror each other), the plane of symmetry in brachiopods cuts through the middle of their valves (valves do not mirror each other). In contrast to bivalves, brachiopods are also typically attached to the substrate by a stalk. They share their feeding organ, the lophophore, in common with the Bryozoa, Entoprocta and Phoronida. Regional accounts of Brachiopoda include Bernard (1972), Hochberg (1996), Kozloff (1996), Lamb and Hanby (2005), Carlton (2007), Baldwin (2009e), Harbo (2011) and Jensen et al. (2018). For annotated records of lampshells reported for Galiano Island, see supplementary materials (Suppl. material 13).

#### Bryozoa

Ehrenberg, 1831

93658150-4D6C-584E-943D-42642A64447D

##### Notes

[**2 classes: 3 orders: 13 families: 15 genera: 17 species**]

‘Bryozoa’ derives from the Ancient Greek βρύον (brúon), “moss”, and ζῷα (zôia), which means “animals.”

Bryozoans, or ‘moss animals’, are colonial animals that are composed of many connected individuals, globally represented by over 6,000 species (Bock and Gordon 2013, WoRMS Editorial Board 2021). While no recently published lists are available to summarise the regional diversity of this phylum, conservative estimates of British Columbia’s bryozoan diversity falls between 210 and 260 species (Baldwin 2009e, Paige Borrett and Graham Gillespie, pers. comm. 2020). A total of 17 bryozoan species are reported for Galiano Island.

Bryozoans are suspension feeders that grow on substrates such as shells, rocks, algae and seagrasses and are prey to micro-predators, such as worms and small crustaceans and gastropods. As bio-constructors, they are known to increase the available habitat and overall diversity of marine ecosystems, making them an important component of many benthic communities. Nevertheless, bryozoans remain a relatively understudied phylum as they have no direct economic value and are generally small, cryptic and difficult to identify without the use of a microscope. Given their obscurity relative to more prominently studied taxa, the diversity of bryozoans around Galiano Island, as throughout the region, is likely under-reported.

**Figure 16.**
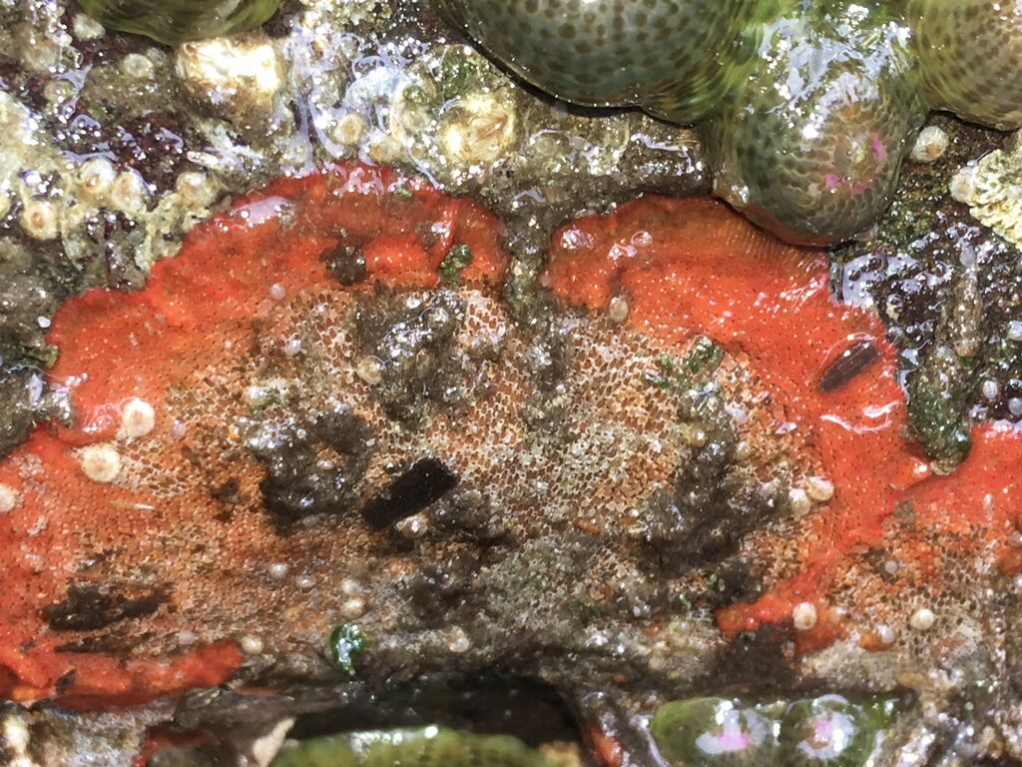
*Schizoporellajaponica* (orange ripple bryozoan) – Photograph by Scott Gilmore.

Of the 17 bryozoan species reported for Galiano Island, one is introduced to British Columbia: *Schizoporellajaponica* Ortmann, 1890 (orange ripple bryozoan) (Fig. 16). Native to Japan, *S.japonica* was described by Powell (1970) as an abundant intertidal organism in the Strait of Georgia, where the species is suspected to have been introduced in the early to mid-1930s along with *Magallanagigas*. The species has since been found in fouling communities from Morro Bay, California to Prince William Sound, Alaska (Ryland et al. 2014, Gartner et al. 2016).

As aquatic suspension feeders, bryozoans pluck food particles from the surrounding water with a specialised structure called a lophophore. Each individual or zooid is typically protected within a covering of calcium carbonate or chitinous material. The protective features and structure of this covering are polymorphic and used to distinguish species. Bryozoan species and their geographic ranges are often listed in invertebrate identification keys (e.g. Kozloff 1996, Lamb and Hanby 2005, Carlton 2007, Harbo 2011, Jensen et al. 2018). Additional resources are available specific to Vancouver Island (O’Donoghue and O’Donoghue 1923, O’Donoghue and O’Donoghue 1926), certain habitats (e.g. Gartner et al. 2016) and for research cruises spanning the Pacific North American coast (Osburn 1950, Osburn 1952, Osburn 1953). For annotated records of bryozoans reported for Galiano Island, see supplementary materials (Suppl. material 14).

#### Phoronida

Hatschek, 1888

E8B6AD31-03F3-51C9-9ECC-735EE6BD9149

##### Notes

[**1 class: 1 order: 1 family: 1 genus: 1 species**]

‘Phoronida’ is the plural form of the phylum’s type genus *Phoronis*, likely derived from the Latin 'Phoronis' (the last name of the mythical Io, priestess of Argos).

**Figure 17.**
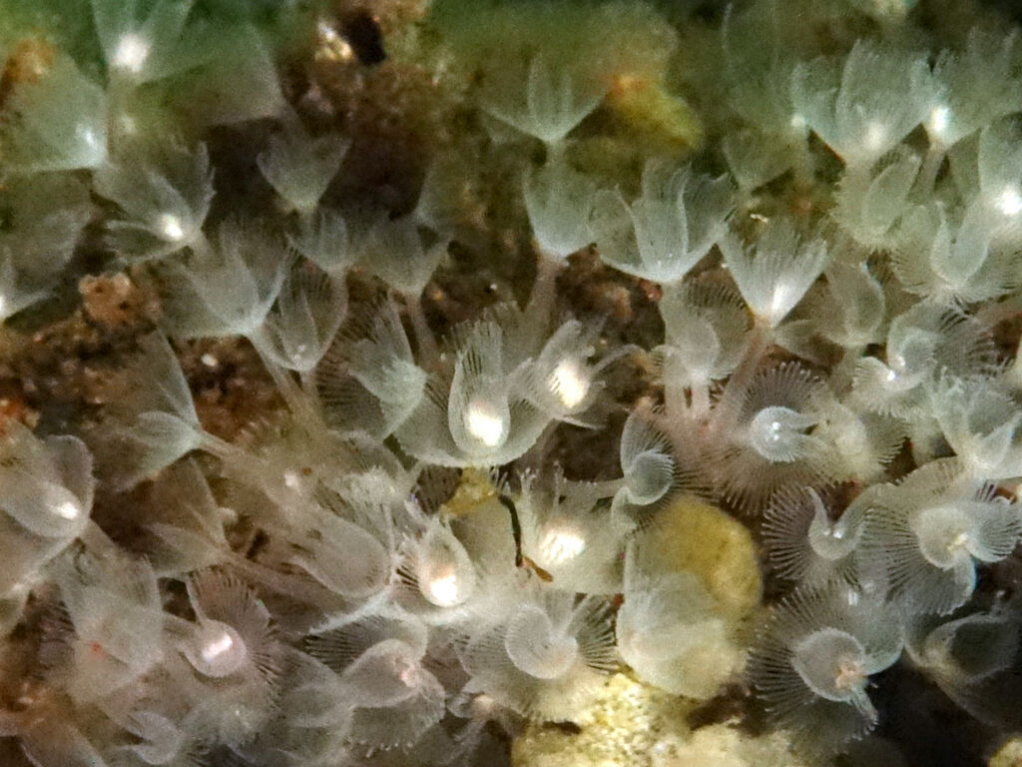
*Phoronisijimai* (white colonial Phoronid) – Photograph by Karolle Wall.

Phoronida (horseshoe worms) are a small phylum of soft-bodied filter-feeding marine animals that form upright tubes of chiton to support their bodies. Worldwide the phylum includes two genera, comprising 13 recognised species (WoRMS Editorial Board 2021). Six species are reported for the Northeast Pacific (Carlton 2007), of which one species is known locally: *Phoronisijimai* (Oka, 1897) (Fig. 17).

Phoronids are benthic organisms that occur at depths ranging from the intertidal zone to about 400 m of depth in all oceans and seas throughout the world, except the Antarctic seas (Temereva et al. 2000, Emig 2003). Their larvae are pelagic and feed on plankton, sometimes comprising a large proportion of zooplanktonic biomass (Temereva 2010). Of the six species reported for the region, it is uncertain which may be expected to occur around Galiano Island, though *Phoronopsisharmeri* (Pixell, 1912) has been reported nearby from the Saanich Inlet (RBCM 976-01136-010). Collections are sparse for this understudied phylum and much doubt surrounds the taxonomy and distributional ecology of the regional fauna. Keys are based on fully developed larval stages, so investigations into this phylum generally entail raising larvae to advanced stages so that they can be reliably compared to illustrations for identification (Johnson and Zimmer 2002). Even then, their determination is usually based on internal anatomy, which often can only be gleaned by examining microscopically thin sections (Carlton 2007). Given the challenges associated with this group, most collections at the Royal British Columbia Museum remain unidentified.

Phoronids are called “horseshoe worms” because the top of their worm-like body contains rows of ciliated tentacles forming a horseshoe shape. This is the lophophore—the feeding organ they share in common with the Brachiopoda, Bryozoa and Entoprocta. References to the phoronids reported for the region may be found in Kozloff (1996), Lamb and Hanby (2005), Carlton (2007), Baldwin (2009e) and Jensen et al. (2018). For annotated records of phoronids reported for Galiano Island, see supplementary materials (Suppl. material 15).

#### Echinodermata

Bruguière, 1791 [ex Klein, 1734]

91E126BC-7FAA-5EC5-9A6B-7F3C5069CAAF

##### Notes

[**5 classes: 14 orders: 20 families: 30 genera: 41 species**]

‘Echinodermata’ is rooted in the Ancient Greek ἐχῖνος (echīnos), meaning “hedgehog”, and δέρμα (derma), “skin”.

Echinoderms are a phylum of marine invertebrates characterised by tough, spiny skin and radial symmetry, containing over 7,000 recognised species worldwide (WoRMS Editorial Board 2021). The phylum comprises five extant classes (WoRMS Editorial Board 2021), all of which are represented locally. These include Asteroidea (sea stars), Crinoidea (crinoids), Echinoidea (sea urchins and sand dollars), Holothuroidea (sea cucumbers) and Ophiuroidea (brittle stars). Of the 111 echinoderm species known to British Columbia (Lambert 1997, Lambert 2000, Lambert and Austin 2007), 41 species are reported for Galiano Island.

**Figure 18.**
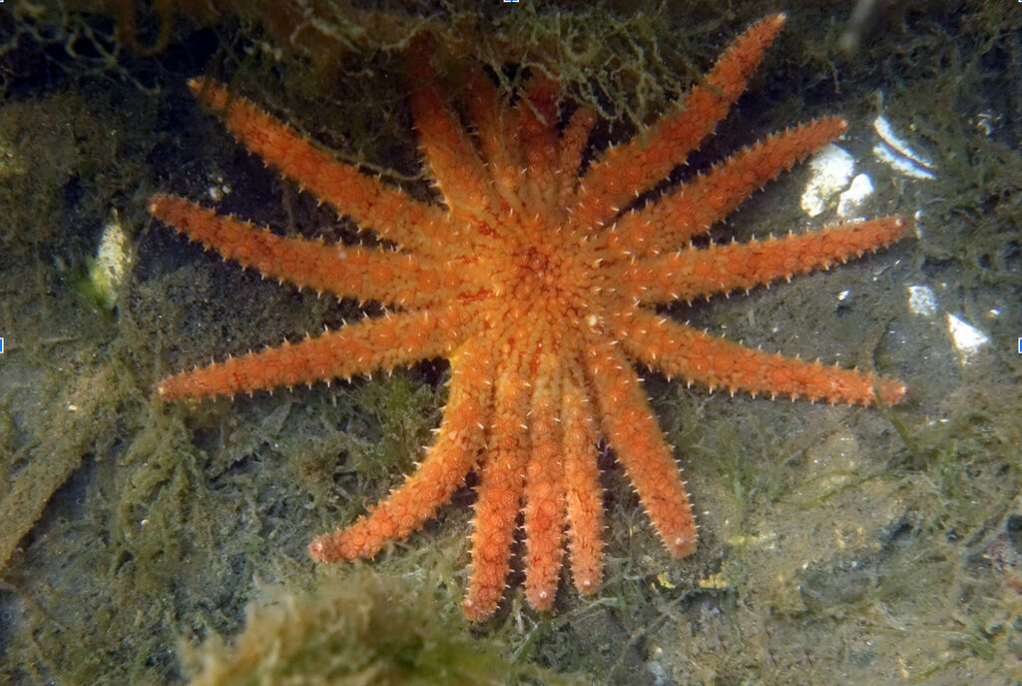
*Pycnopodiahelianthoides* (sunflower star) – Photograph by Karolle Wall.

Echinoderms, especially sea stars, are an ecologically important component of the marine fauna of the Northeast Pacific. The term “keystone species” was first coined by Paine (1966) to describe the dominant influence of the predatory *Pisasterochraceus* (Brandt, 1835) (ochre sea star) over the structure and composition of intertidal communities. Since 2013, sea stars, such as *Pisasterochraceus* and *Pycnopodiahelianthoides* (Brandt, 1835) (sunflower sea star) (Fig. 18), have undergone severe declines in the region due to sea star wasting syndrome and their populations have not yet recovered (Miner et al. 2018). A recent study by Aquino et al. (2021) presents evidence indicating that sea star wasting syndrome may be the consequence of elevated microbial activities induced by high organic matter concentrations, which causes depleted oxygen conditions at the animal-water interface, such that sea stars cannot meet the respiratory oxygen demand of tissues. This phenomenon is likely exacerbated under warmer ocean conditions and anthropogenic nutrient pollution (Aquino et al. 2021).

Under-represented echinoderms in the Galiano Island record include the holothuroids and ophiuroids. This gap is likely due to sampling bias, as many of these taxa are found at deeper depths than have been sampled locally or otherwise tend to be buried beneath rock and sediment. Gaps otherwise relate to limits in taxonomic knowledge. For example, the genus *Henricia* Gray, 1840 is highly variable and in need of clarification. Genetic studies are underway to sort out many suspected new species of *Henricia* between Alaska and California, led by the efforts of Roger Clark, Doug Eernisse, ‪Megumi Strathmann and Christopher Mah. *Leptasteriashexactis* (Stimpson, 1862) also belongs to a species complex (*Leptasteriasaequalis* species complex) requiring further study. Regional accounts of echinoderms include Kyte (1969), Kozloff (1996), Lambert (1997), Lambert (2000), Lamb and Hanby (2005), Carlton (2007), Lambert and Austin (2007), Harbo (2011), Lambert and Boutillier (2011) and Jensen et al. (2018). For annotated records of echinoderms reported for Galiano Island, see supplementary materials (Suppl. material 16).

#### Tunicata

Lamarck, 1816

BE50B721-CF24-532B-9950-A8D7A7E75883

##### Notes

[**1 class: 3 orders: 12 families: 24 genera: 33 species**]

The name ‘Tunicata’ comes from the Latin *tunicatus*, past particle of the verb *tunicare*, “to clothe with a tunic.”

Tunicates are filter-feeding marine animals that derive their name from their protective exoskeleton, called the tunic, which is formed of proteins and carbohydrates. A subphylum of Chordata, Tunicata are traditionally divided into three classes—Ascidiacea, Thaliacea and Appendicularia—though recent evidence suggests this group is paraphyletic (Delsuc et al. 2018). Ascidiacea, or ascidians, are the most diverse and common class of tunicates and are, indeed, the most well represented class in this dataset. There are around 2,815 described ascidian species worldwide (Shenkar and Swalla 2011). Four of the 71 taxa reported from the coast of British Columbia (Royal British Columbia Museum 2021) are extremely deep-water species, while five are restricted to northern BC and are, thus, not expected for Galiano Island. Of the 62 species that might be expected to occur locally, 33 species have been documented to date.

**Figure 19.**
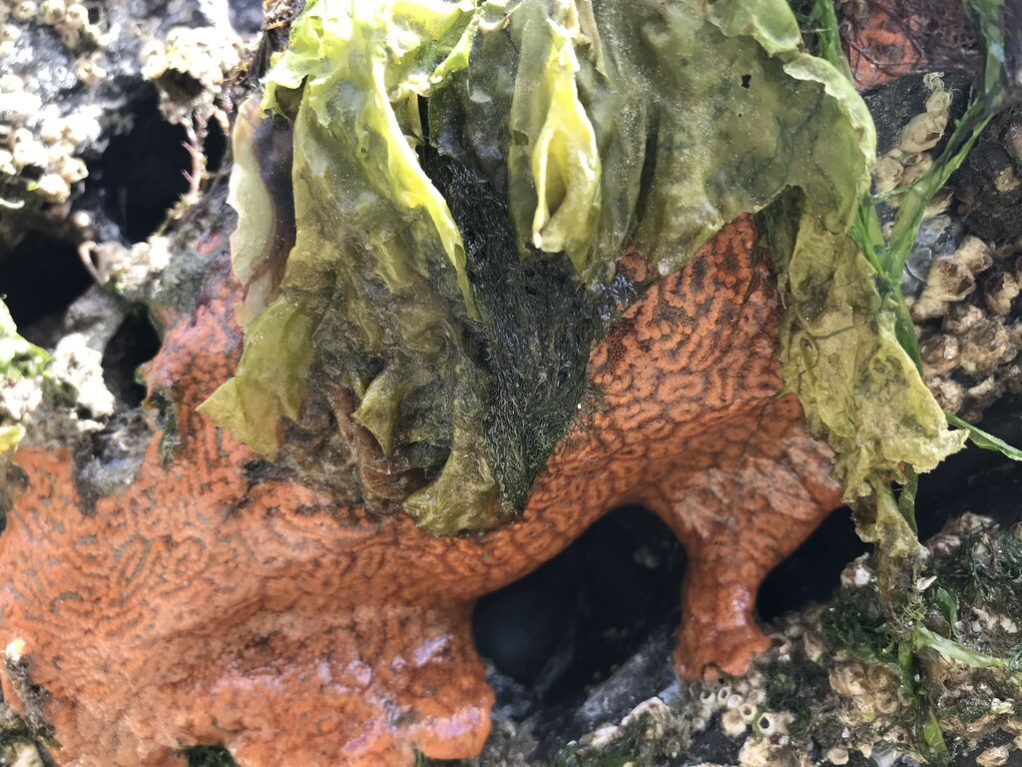
*Botrylloidesviolaceus* (lined compound ascidian) – Photograph by Jennifer Grant.

Ascidians are sessile organisms found at all ocean depths worldwide, attaching to natural surfaces such as rocky outcrops and invertebrate shells, as well as artificial structures such as pilings, docks and boat hulls. Notable reports for Galiano Island include several non-native species that have a long history of invasion, often resulting in measurable impacts in other parts of Canada and around the world (e.g. *Lambert 2007*, Arsenault et al. 2009, Arens et al. 2011, Zhan et al. 2015). Native to Japan, *Botrylloidesviolaceus* Oka, 1927 (lined compound ascidian) (Fig. 19) was first introduced to the Pacific coast in California in 1973 and spread northwards along the coast to Canada (Lambert 2003). *Didemnumvexillum* Kott, 2002 (carpet sea squirt) is also thought to be native to Japan and was first reported for British Columbian waters in 2003 (Lambert 2009). Other reported taxa, such as *Cionasavignyi* Herdman, 1882 (sea vase) and *Diplosomalisterianum* (Milne Edwards, 1841) (grey encrusting compound tunicate), have unknown provenance and may, thus, be described as cryptogenic.

Tunicates are generally under-represented in this study as they are easily overlooked; new species are still being discovered in British Columbia (Lambert 2019). The Thaliacea and Appendicularia, in particular, are poorly represented—the former limited to a singular dive observation that has not been identified beyond the level of class and the latter not represented at all. Unlike ascidians, these groups include exclusively pelagic species that are more abundant in warmer waters, with low probability of being detected in surveys around Galiano Island.

Identification of tunicates is often based on gross morphological features, though dissection is occasionally required. Ascidians may be categorised as solitary, social or colonial according to variations on a basic body plan. The individuals, called zooids, are very small (measuring a few mm), but the colonies can be up to several metres large. Though tunicates are generally easily overlooked, often confused with other groups and challenging to identify to species, there are excellent resources available to support their identification, including: popular field guides (Gotshall and Laurent 1980, Lamb and Hanby 2005, Harbo 2011, Jensen et al. 2018); taxonomic keys (Kozloff 1996, Carlton 2007); published journal articles (e.g. Name and Willard 1945, Lambert and Sanamyan 2001, Lambert 2003, Lambert 2019); and online summaries (e.g. Baldwin 2009f, Gartner 2014). For annotated records of tunicates reported for Galiano Island, see supplementary materials (Suppl. material 17).

#### Actinopterygii

Klein, 1885

7A8185FA-5E62-5F7C-969A-B6059AC761E9

##### Notes

[**11 orders: 29 families: 58 genera: 78 species**]

Both Actinopterygii (ray-finned fish) and Chondrichthyes (cartilaginous fish) are treated below.

‘Actinopterygii’ is formed from the Latin 'actino-', “having rays”, and the Ancient Greek πτέρυξ (ptérux), meaning “wings” or “fins.” ‘Chondrichthyes’ comes from the Greek χόνδρος (khóndros), meaning "cartilage", and ἰχθύς (ikhthū́s), meaning "fish".

Actinopterygii contain over 33,000 described taxa and Chondrichthyes (cartilaginous fish) over 1,100 taxa, including both freshwater and marine species (Guinot and Cavin 2016, Weigmann 2016). Over 1,500 marine species are estimated to occur between the Beaufort Sea, Alaska, to the tip of Baja California, Mexico (Milton Love, pers. comm. 2020) and over 400 species reported for Canadian waters in the Northeast Pacific (Peden 2002). To date, 78 species of ray-finned fish and four species of cartilaginous fish have been reported for Galiano Island.

Marine fishes dwell in various habitats, from deep sea (pelagic) to near-shore and intertidal environments. Taxa under-represented in this dataset include chondrichthyes (cartilaginous fish), myxinids (hagfish) and osmerids (smelts). These gaps reflect search efforts mostly biased toward rocky reefs and exposed beach locales, to waters < 30 m in depth. Agonids (poachers), pleuronectids (right-eyed flounders) and zoarcids (eelpouts) are also under-represented. These are primarily subtidal, soft substrate inhabitants, indicating that sampling has been limited in these habitats.

**Figure 20.**
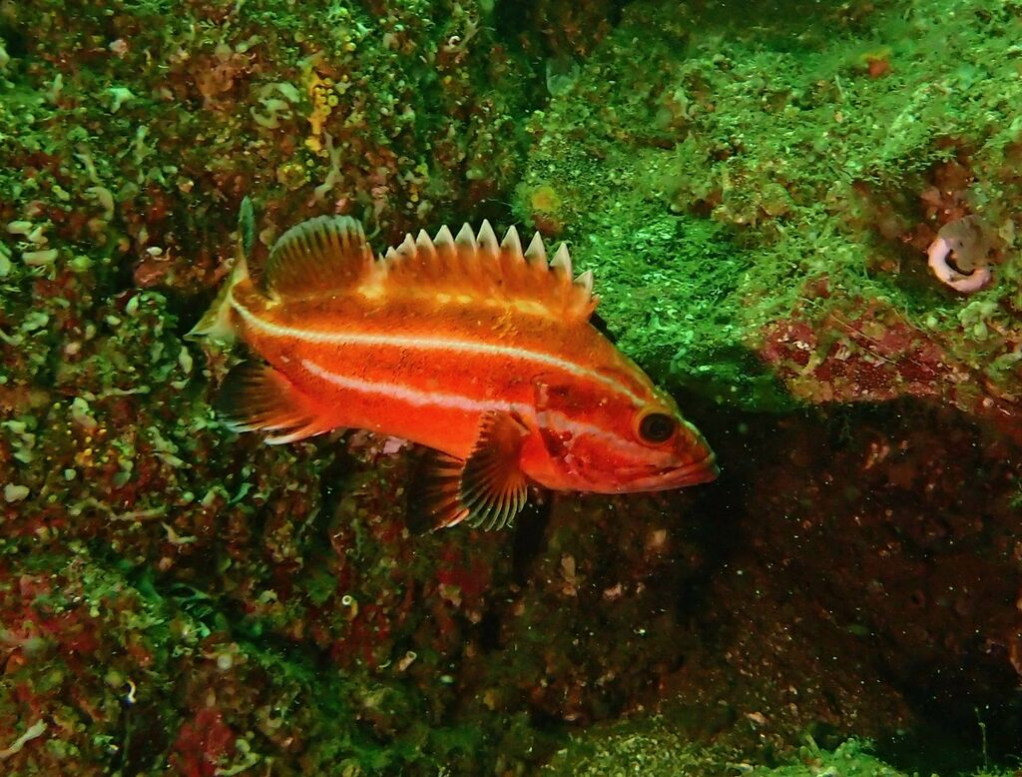
*Sebastesruberrimus* (yelloweye rockfish) – Photograph by Don Gordon.

Rockfish, *Sebastes* G. Cuvier, 1829, are a long-lived and diverse genus of fish in the Northeast Pacific, 11 of which are represented in the Galiano Island record. Species present include the threatened *Sebastesmaliger* (Jordan & Gilbert, 1880) (quillback rockfish) and *S.ruberrimus* (Cramer, 1895) (yellow-eye rockfish) (Fig. 20), the latter of which is ranked as a species of special concern in Canada (Fisheries and Oceans Canada 2018b). This species may attain large sizes (~ 1 m, 12.6 kg). However, as with other species in the genus, it is slow to mature and reproduce and is, therefore, vulnerable to overfishing and fatality due to barotrauma from catch and release (Rankin et al. 2017). As a consequence, many species of *Sebastes* are at low levels of abundance in the region (Fisheries and Oceans Canada 2021). Spatially-managed rockfish conservation areas that remain permanently closed to all fishing and extraction activities are vital to ensure their recovery (Rankin et al. 2017).

*Squalussuckleyi* Girard, 1854 (North Pacific spiny dogfish), reported for Galiano Island, is a species of special concern in Canada (COSEWIC 2011). The near-threatened *Hexanchusgriseus* (Bonnaterre, 1788) (bluntnose sixgill shark), is also known from adjacent waters around Mayne Island, BC. This species likely occurs around Galiano Island, though it is not reported in this dataset. One unusual report for Galiano Island is *Trachipterusaltivelis* Kner, 1859 (king-of-the-salmon), an outer coastal species likely represented by a specimen carried in from offshore waters. Introduced fishes, such as *Alosasapidissima* (A. Wilson, 1811) (American shad), *Salmosalar* Linnaeus, 1758 (Atlantic salmon) and *Salmotrutta* Linnaeus, 1758 (brown trout), are documented from waters not far from Galiano Island, though they have not yet been reported locally. References for the fishes of the Pacific coast of North America include Clemens and Wilby (1961), Hart (1973), Peden (2002), Lamb and Hanby (2005), Love et al. (2005), De Maddalena et al. (2007), Lamb and Edgell (2010), Harbo (2011), Love (2011) and Butler et al. (2012). For annotated records of ray-finned fishes reported for Galiano Island, see supplementary materials (Suppl. material 18).

#### Chondrichthyes

Huxley, 1880

CA4A9B06-66B9-53FF-A4B6-307213C41008

##### Notes

[**3 orders: 3 families: 3 genera: 4 species**]

Chondrichthyes are discussed above, alongside Actinopterygii. For annotated records of cartilaginous fishes reported for Galiano Island, see supplementary materials (Suppl. material 19).

#### Mammalia

Linnaeus, 1758

9A4F437C-3374-5FDD-8609-687311C56C14

##### Notes

[**2 orders: 7 families: 12 genera: 13 species**]

The name ‘Mammalia’ was coined in 1758 by Linnaeus, from Late Latin (neuter plural) 'mammalis', meaning “of the breast.”

Mammalia are a class of vertebrates containing 6,400 species worldwide (Burgin et al. 2018). Within British Columbia, 31 marine mammal species are represented among two orders, including Carnivora (carnivores) and Cetacea (cetaceans) (Ford 2014). Twenty-two marine mammals have been documented within the Salish Sea, including fifteen cetaceans, five species of pinniped and two mustelids (Ford 2014). Of these taxa, only 12 are known to commonly occur in the region. Thirteen species of marine mammal have been reported in waters around Galiano Island to date.

Among locally occurring cetaceans are: baleen whales, distinguished by their enlarged head containing baleen plates suspended from the upper jaw, their double blowhole and their thick blubber; and toothed whales, distinguished by their teeth and single external blowhole. Certain cetaceans, such as *Balaenopteraphysalus* (Linnaeus, 1758) (fin whale) and *Balaenopteraacutorostrata* Lacépède, 1804 (common minke whale) have been recorded infrequently in local waters, whereas others, such as *Megapteranovaeangliae* Borowski, 1781 (humpback whale) and *Phocoenaphocoeana* (Linnaeus, 1758) (harbour porpoise), are commonly seen. Among carnivores, both aquatic pinnipeds, such as *Phocavitulinarichardii* Gray, 1864 (harbour seal) and semi-aquatic fissipeds, such as *Lontracanadensis* (Schreber, 1777) (river otter), are represented. Presently, there are no known haulouts of *Miroungaangustirostris* (Gill, 1866) (northern elephant seal) around Galiano Island, though they are known not far away on Salt Spring Island.

**Figure 21.**
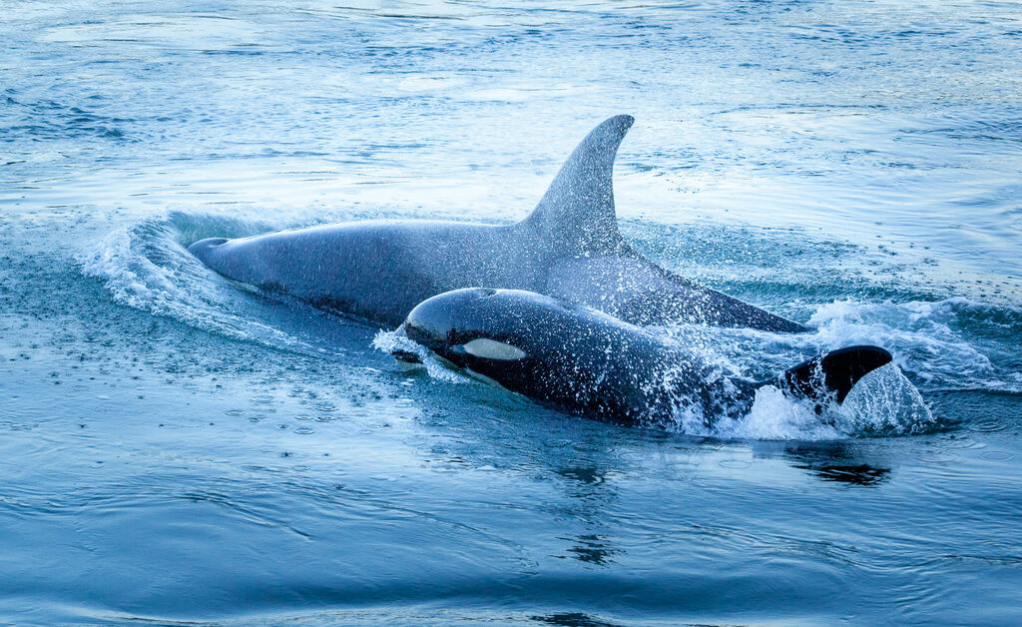
Mother (J41) with baby (J51) of the Southern Resident Killer Whale J Pod – Photograph by Karoline Cullen.

Populations of *Eschrichtiusrobustus* (Lilljeborg, 1861) (grey whale), *M.novaeangliae*, *P.phocoena* and the southern resident population of *Orcinusorca* (Linnaeus, 1758) (orca) (Fig. 21) are currently ranked as species of conservation concern in the region (Fisheries and Oceans Canada 2009a, Fisheries and Oceans Canada 2010, Fisheries and Oceans Canada 2013, Fisheries and Oceans Canada 2018a). Northern resident, transient and offshore orcas are also known to traverse local waters and are listed as threatened within Canada (Fisheries and Oceans Canada 2007, Fisheries and Oceans Canada 2009b, Fisheries and Oceans Canada 2018a). Regional accounts of marine mammals include Williams and Thomas (2007), Ford (2014), Nagorsen (2014) and Harvey et al. (2017). For annotated records of mammals reported for Galiano Island, see supplementary materials (Suppl. material 20).

## Discussion

Extensive efforts are being made to promote the availability of biodiversity data, which have increased considerably over the course of the last decade (Ball-Damerow et al. 2019). However, research has shown that the full potential for community science to contribute to these efforts has yet to be realised (Theobald et al. 2015). In this study, we underscore this potential, as exemplified by the Pacific Marine Life Surveys (PMLS), which account for 60% of novel species reported for Galiano Island over the last 160 years, representing the largest proportion of our knowledge of the local marine fauna.

While there is growing recognition of the value of involving citizen scientists in biodiversity research (Chandler et al. 2017, Peter et al. 2021), this dataset highlights the potential for them to play a more integrated role in the global research community. The contributions of citizen scientists to biodiversity research have been valued at $2.5 billion in-kind annually and cover many essential biodiversity variables with broad taxonomic and geographic reach (Theobald et al. 2015, Chandler et al. 2017). Still, there remain many geographic and taxonomic gaps (Chandler et al. 2017) and data often do not reach the peer-reviewed scientific literature (Theobald et al. 2015). The PMLS set an example for how communities can help fill these gaps in collaboration with research scientists, advancing more inclusive and systematic approaches to biodiversity research.

Within the Salish Sea, PMLS records currently account for ~ 248,000 observations from > 4,800 dives, documenting > 1,100 species. These records have supported numerous studies, providing information for a broad range of taxonomic and ecological research (e.g. Jensen 2006, Marliave et al. 2011, Calder et al. 2015, Millen 2016, Schultz et al. 2016, Marliave et al. 2018a, Marliave et al. 2018b, Marliave et al. 2018c, Gravem et al. 2020, Marliave and Borden 2020). The breadth and depth of these surveys is remarkable when considered alongside other sources of data available for Galiano Island. Scaled up, PMLS records, indeed, hold great potential to shore up biodiversity baselines for localities throughout the Salish Sea. Our analysis thus reveals how community-integrated biodiversity research promises to resolve a better picture of biodiversity at multiple scales in the region, which may be facilitated through the design of more inclusive biodiversity informatics frameworks.

Our synthesis sheds light on the complementarity of various types of sampling effort, pointing to ways in which improved data sharing and collaboration might enable more systematic biodiversity research practices. These lessons derive from a basic realisation: different types of sampling effort have distinct constraints in terms of taxonomic, spatial and temporal resolution, but when combined, can contribute to more robust biodiversity baselines. Below, we discuss the limitations and complementarity of the various data sources synthesised in this study.

**Collections**

Collections are of tremendous value for taxonomic research, not only providing the oldest records in this study, but also yielding the highest ratio of novel species reports to occurrence records (1:4) as compared to crowd-sourced observations (1:38), ecological surveys (1:66) and PMLS records (1:67). Voucher specimens are relatively few in this study, with low spatial coverage, yet offer improved taxonomic resolution of difficult groups such as molluscs, crustaceans and annelids. Specimens are necessary to identify many taxa, which often entails microscopy, dissection and genetic study. Yet, processing specimens and making such data available is notoriously slow. Worldwide, it is estimated that only 10% of biospecimen data are available in digital form (Ball-Damerow et al. 2019). Thus, where the availability of specimen data poses a limiting factor, biodiversity baselines may be supplemented by other data sources. On the other hand, established baselines can reveal insights and data gaps, providing information for more targeted collection activities. In this study, historical collections had limited spatial resolution, with greater coordinate uncertainty as compared with other sources of data. We also found identification errors in our review of collections. Data validation thus remains a perennial concern, not only for crowd-sourced data, but also formal research collections.

**Crowd-sourced data**

Observations on iNaturalist and the BC Cetacean Sightings Network (BCCSN) have greater spatial coverage than other sources included in this dataset, yet are more diffuse than systematic efforts such as the PMLS. In this study, data from the PMLS and research collections largely predate observations derived from these platforms. That crowd-sourced observations nevertheless contribute 18% of novel species reports in this study demonstrates the value of these data, which may supplement baseline datasets, even in areas historically subject to intensive search efforts. In contrast to PMLS observations, which are limited from the subtidal to a depth of 30 m, these records largely represent casual intertidal beach-combing and nearshore cetacean observations. Exceptions include iNaturalist observations yielded by SCUBA and snorkelling and those posted from research programmes, such as those of Chu and Leys (2010) and Chu and Leys (2012) (Note: in analysing this dataset, Chu’s iNaturalist observations, which were collected by ROV, were categorised as ecological study data). In this respect, crowd-sourced data can again be compared with specimen records, insofar as these data sources may represent heterogeneous types of sampling effort. Expert review is important to validate more challenging taxa, which is facilitated by the availability of good quality images (e.g. iNaturalist), though certain taxa cannot be validated without recourse to specimens. Validity is otherwise recorded in terms of levels of confidence (e.g. BCCSN).

**Ecological studies**

Ecological studies are more narrowly focused than other sampling approaches represented in this dataset. These studies have limited temporal and spatial resolution and focus on: a) anadromous salmonids (Erickson 2000); and b) biota associated with deep-sea glass sponge reefs from 69–119 m in depth (Chu and Leys 2010, Chu and Leys 2012). Given their focus on areas either inaccessible to or overlooked by other types of sampling effort, ecological studies fill certain gaps in the historical record. They also encode correlations between species occurrences and relevant environmental and trait data, thereby supporting ecological analyses. Ecological surveys may be fairly resource intensive as compared with other collection efforts, yet may add substantial value to ongoing biodiversity inventories. In turn, ecological research can benefit from the baselines established by historical biodiversity datasets (Marliave et al. 2011, Marliave et al. 2018a, Marliave et al. 2018b, Simon et al. 2021).

**Pacific Marine Life Surveys (PMLS)**

Ongoing since 1967, the PMLS are systematic marine biodiversity surveys, based on the roving diver methodology (Martell 1999). This intuitive search method supports the documentation of a wide breadth of biodiversity, including rare, endangered and invasive species, as a greater number of taxa can be detected than in transect-based surveys. Data from the PMLS reflect certain limitations, including low resolution of certain taxonomically challenging groups (e.g. annelids). However, in some cases (e.g. sponges), these limitations have been overcome by collecting specimens and working in concert with taxonomic specialists. In addition to the input of numerous expert authorities, the PMLS have benefited from cumulative natural history knowledge that can only be gained through long-sustained fieldwork. While PMLS dive records cannot be independently verified, they arguably retain a high standard of validity, having been recorded by highly skilled naturalists, based on systematic protocols, including the collection of detailed metadata and specimens to support ongoing data curation.

### Conclusion: Strengthening connections in the biodiversity research community

Our study shows the potential for communities to synthesise comprehensive baseline datasets, which may form the basis for more coordinated and systematic biodiversity monitoring efforts. To realise this potential, however, the global community has been challenged to rethink the dynamics of biodiversity research practice (Theobald et al. 2015). Best practices for involving community members in biodiversity observation tend to construe citizen scientists as “contributors" and the scientific research community as the “users” (Chandler et al. 2017). Too often, citizen scientists are instrumentalised, viewed as resources useful for experts abstracted from local communities and their motivations interpreted strictly in this context (Lawrence 2006, Chandler et al. 2017, Peter et al. 2021). Evidence suggests that more coordinated approaches to biodiversity research, which strengthen connections between professionals and non-professionals, can help to mobilise significant amounts of biodiversity data at a time of critical need (Theobald et al. 2015). The need to foster more meaningful expressions of community-based biodiversity research is especially critical in regions such as the Salish Sea, where high biodiversity meets the threat of growing human populations and development (Kraus and Hebb 2020, Sobocinski 2021).

This study sets an example for a community science initiative that both contributes to the global biodiversity research community and actively uses its own data to further its research objectives in collaboration with research scientists. We join Theobald et al. (2015) in advancing a paradigm shift towards more reciprocal practices that enable diverse communities to participate more fully in biodiversity research, fostering mutual interest in the data generated through these efforts. One way of achieving this ideal is through the development of biodiversity informatics frameworks that engage local communities, not only in the collection, but also the curation and analysis of biodiversity data. This proposed framework honours the role of local and regional place-based experts, takes advantage of the relative trade-offs between different sampling methods and leverages the complementary resources that exist in diverse communities of practice.

Depending on the data available from place to place, systematic community-based sampling efforts, as exemplified by the PMLS in this study, may prove critical in the establishment of robust biodiversity baselines. When combined with citizen science observations, ecological data and research collections, these surveys can also bolster the data available through major aggregators, such as the Global Biodiversity Information Facility (GBIF). The open-source biodiversity informatics framework that we are developing harnesses readily accessible tools such as Google Sheets, GitHub and JavaScript code to facilitate curation processes that integrate data available through iNaturalist and GBIF with other sources of local biodiversity knowledge. This inclusive data management system will enable communities to synthesise data from a diversity of sources, establishing baselines for localised biodiversity monitoring efforts. Scaled up, this system can support distributed biodiversity monitoring networks that synthesise data generated by communities throughout regions such as the Salish Sea. Our study represents a gesture toward the development of such a framework at a time of critical need.

## Supplementary Material

XML Treatment for Porifera

XML Treatment for Cnidaria

XML Treatment for Ctenophora

XML Treatment for Nemertea

XML Treatment for Platyhelminthes

XML Treatment for Chaetognatha

XML Treatment for Mollusca

XML Treatment for Annelida

XML Treatment for Sipuncula

XML Treatment for Crustacea

XML Treatment for Entoprocta

XML Treatment for Brachiopoda

XML Treatment for Bryozoa

XML Treatment for Phoronida

XML Treatment for Echinodermata

XML Treatment for Tunicata

XML Treatment for Actinopterygii

XML Treatment for Chondrichthyes

XML Treatment for Mammalia

3B5DBDFA-97F7-5FA4-8EF4-083B2A8B3F77 10.3897/BDJ.10.e76050.suppl1 Supplementary material 1 Marine animals reported for Galiano Island, BC, Canada (1859–2021) Data type formatted checklist Brief description Formatted checklist summarising marine animal diversity reported for Galiano Island, British Columbia, Canada (1859–2021). File: oo_640501.pdf https://binary.pensoft.net/file/640501 Emily Adamczyk, Antranig Basman, Jackson Chu, Karin Fletcher, Heidi Gartner, Charlie Gibbs, Donna Gibbs, Scott Gilmore, Rick Harbo, Leslie Harris, Elaine Humphrey, Andy Lamb, Philip Lambert, Neil McDaniel, Jessica Scott, Andrew Simon

064E1A0A-9731-5855-8161-51A69B62677A 10.3897/BDJ.10.e76050.suppl2 Supplementary material 2 Porifera of Galiano Island, British Columbia, Canada Data type taxonomic summary and catalogue of occurrence records Brief description Curated taxonomic summary and catalogue of occurrence records documenting Porifera reported for Galiano Island, British Columbia, Canada, in Darwin Core Standard format. File: oo_640496.xlsx https://binary.pensoft.net/file/640496 Antranig Basman, Jackson Chu, Charlie Gibbs, Donna Gibbs, Andy Lamb, Neil McDaniel, Andrew Simon

9BA106DA-3A01-5C53-B141-0D3683AC3E80 10.3897/BDJ.10.e76050.suppl3 Supplementary material 3 Cnidaria of Galiano Island, British Columbia, Canada Data type taxonomic summary and catalogue of occurrence records Brief description Curated taxonomic summary and catalogue of occurence records documenting Cnidaria reported for Galiano Island, British Columbia, Canada, in Darwin Core Standard format. File: oo_640499.xlsx https://binary.pensoft.net/file/640499 Antranig Basman, Charlie Gibbs, Donna Gibbs, Andy Lamb, Andrew Simon

FC8FC17C-0159-57DE-9E47-244BC007C872 10.3897/BDJ.10.e76050.suppl4 Supplementary material 4 Ctenophora of Galiano Island, British Columbia, Canada Data type taxonomic summary and catalogue of occurrence records Brief description Curated taxonomic summary and catalogue of occurrence records documenting Ctenophora reported for Galiano Island, British Columbia, Canada, in Darwin Core Standard format. File: oo_640886.xlsx https://binary.pensoft.net/file/640886 Antranig Basman, Charlie Gibbs, Donna Gibbs, Andy Lamb, Andrew Simon

17AB9FA9-CAE1-5C67-990D-DF60E23536BC 10.3897/BDJ.10.e76050.suppl5 Supplementary material 5 Nemertea of Galiano Island, British Columbia, Canada Data type taxonomic summary and catalogue of occurrence records Brief description Curated taxonomic summary and catalogue of occurrence records documenting Nemertea reported for Galiano Island, British Columbia, Canada, in Darwin Core Standard format. File: oo_640890.xlsx https://binary.pensoft.net/file/640890 Antranig Basman, Charlie Gibbs, Donna Gibbs, Scott Gilmore, Andy Lamb, Andrew Simon

90DBE250-CE4A-598A-9AD1-E200183E75FD 10.3897/BDJ.10.e76050.suppl6 Supplementary material 6 Platyhelminthes of Galiano Island, British Columbia, Canada Data type taxonomic summary and catalogue of occurence records Brief description Curated taxonomic summary and catalogue of occurrence records documenting Platyhelminthes reported for Galiano Island, British Columbia, Canada, in Darwin Core Standard format. File: oo_640892.xlsx https://binary.pensoft.net/file/640892 Antranig Basman, Charlie Gibbs, Donna Gibbs, Scott Gilmore, Andy Lamb, Andrew Simon

F8689085-280B-59E5-8352-5CB71A27A118 10.3897/BDJ.10.e76050.suppl7 Supplementary material 7 Chaetognatha of Galiano Island, British Columbia, Canada Data type taxonomic summary and catalogue of occurrence records Brief description Curated taxonomic summary and catalogue of occurrence data documenting Chaetognatha reported for Galiano Island, British Columbia, Canada, in Darwin Core Standard format. File: oo_640884.xlsx https://binary.pensoft.net/file/640884 Antranig Basman, Charlie Gibbs, Donna Gibbs, Elaine Humphrey, Andy Lamb, Andrew Simon

30C2243F-36DA-5316-B627-5DA7236E1F3C 10.3897/BDJ.10.e76050.suppl8 Supplementary material 8 Mollusca of Galiano Island, British Columbia, Canada Data type taxonomic summary and catalogue of occurrence records Brief description Curated taxonomic summary and catalogue of occurrence records documenting Mollusca reported for Galiano Island, British Columbia, Canada, in Darwin Core Standard format. File: oo_640505.xlsx https://binary.pensoft.net/file/640505 Antranig Basman, Karin Fletcher, Charlie Gibbs, Donna Gibbs, Rick Harbo, Andy Lamb, Andrew Simon

58FED924-4613-5BA1-AFBA-04131BF22CB4 10.3897/BDJ.10.e76050.suppl9 Supplementary material 9 Annelida of Galiano Island, British Columbia, Canada Data type taxonomic summary and catalogue of occurrence records Brief description Curated summary and catalogue of occurrence records documenting Annelida reported for Galiano Island, British Columbia, Canada, in Darwin Core Standard format. File: oo_640880.xlsx https://binary.pensoft.net/file/640880 Antranig Basman, Charlie Gibbs, Donna Gibbs, Scott Gilmore, Leslie Harris, Andy Lamb, Andrew Simon

548BA60A-1D26-5984-9BF1-A2059E54A47B 10.3897/BDJ.10.e76050.suppl10 Supplementary material 10 Sipuncula of Galiano Island, British Columbia, Canada Data type taxonomic summary and catalogue of occurrence records Brief description Curated summary and catalogue of occurrence records documenting Sipuncula reported for Galiano Island, British Columbia, Canada, in Darwin Core Standard format. File: oo_640893.xlsx https://binary.pensoft.net/file/640893 Antranig Basman, Charlie Gibbs, Donna Gibbs, Scott Gilmore, Andy Lamb, Andrew Simon

FB6F1CD6-6AE8-5EEC-B367-E69CE930CDF3 10.3897/BDJ.10.e76050.suppl11 Supplementary material 11 Crustacea of Galiano Island, British Columbia, Canada Data type taxonomic summary and catalogue of occurrence records Brief description Curated taxonomic summary and catalogue of occurrence records documenting Crustacea reported for Galiano Island, British Columbia, Canada, in Darwin Core Standard format. File: oo_640882.xlsx https://binary.pensoft.net/file/640882 Antranig Basman, Charlie Gibbs, Donna Gibbs, Emily Adamczyk, Andy Lamb, Andrew Simon

9E94EA81-3D8F-54F2-9F2B-6D99D04F9650 10.3897/BDJ.10.e76050.suppl12 Supplementary material 12 Entoprocta of Galiano Island, British Columbia, Canada Data type taxonomic summary and catalogue of occurrence records Brief description Curated taxonomic summary and catalogue of occurrence records documenting Entoprocta reported for Galiano Island, British Columbia, Canada, in Darwin Core Standard format. File: oo_640888.xlsx https://binary.pensoft.net/file/640888 Antranig Basman, Charlie Gibbs, Donna Gibbs, Elaine Humphrey, Andy Lamb, Andrew Simon

E164482E-6CC3-5933-943E-A839A05C1DFE 10.3897/BDJ.10.e76050.suppl13 Supplementary material 13 Brachiopoda of Galiano Island, British Columbia, Canada Data type taxonomic summary and catalogue of occurrence records Brief description Curated taxonomic summary and catalogue of occurrence records documenting Brachiopoda reported for Galiano Island, British Columbia, Canada, in Darwin Core Standard format. File: oo_640883.xlsx https://binary.pensoft.net/file/640883 Antranig Basman, Jackson Chu, Charlie Gibbs, Donna Gibbs, Andy Lamb, Andrew Simon

2FFB63AB-9FB4-5A4C-949E-FE1433593139 10.3897/BDJ.10.e76050.suppl14 Supplementary material 14 Bryozoa of Galiano Island, British Columbia, Canada Data type taxonomic summary and catalogue of occurrence records Brief description Curated taxonomic summary and catalogue of occurrence records documenting Bryozoa reported for Galiano Island, British Columbia, Canada, in Darwin Core Standard format. File: oo_640508.xlsx https://binary.pensoft.net/file/640508 Antranig Basman, Heidi Gartner, Charlie Gibbs, Donna Gibbs, Andy Lamb, Andrew Simon

9EB39B55-CA0B-524D-8B52-0E91444D521F 10.3897/BDJ.10.e76050.suppl15 Supplementary material 15 Phoronida of Galiano Island, British Columbia, Canada Data type taxonomic summary and catalogue of occurrence records Brief description Curated taxonomic summary and catalogue of occurrence records documenting Phoronida reported for Galiano Island, British Columbia, Canada, in Darwin Core Standard format. File: oo_640891.xlsx https://binary.pensoft.net/file/640891 Antranig Basman, Charlie Gibbs, Donna Gibbs, Elaine Humphrey, Andy Lamb, Andrew Simon

7B52B283-15F6-53F9-BE16-5FD7C0036579 10.3897/BDJ.10.e76050.suppl16 Supplementary material 16 Echinodermata of Galiano Island, British Columbia, Canada Data type taxonomic summary and catalogue of occurrence records Brief description Curated taxonomic summary and catalogue of occurrence records documenting Echinodermata reported for Galiano Island, British Columbia, Canada, in Darwin Core Standard format. File: oo_640887.xlsx https://binary.pensoft.net/file/640887 Antranig Basman, Charlie Gibbs, Donna Gibbs, Andy Lamb, Philip Lambert, Andrew Simon

C0B0E314-2368-53AB-8D93-3E1DE8802976 10.3897/BDJ.10.e76050.suppl17 Supplementary material 17 Tunicata of Galiano Island, British Columbia, Canada Data type taxonomic summary and catalogue of occurrence records Brief description Curated taxonomic summary and catalogue of occurrence records documenting Tunicata reported for Galiano Island, British Columbia, Canada, in Darwin Core Standard format. File: oo_640894.xlsx https://binary.pensoft.net/file/640894 Antranig Basman, Heidi Gartner, Charlie Gibbs, Donna Gibbs, Andy Lamb, Andrew Simon

9428DB10-893D-5D69-AAE0-B5171784EDD2 10.3897/BDJ.10.e76050.suppl18 Supplementary material 18 Actinopterygii of Galiano Island, British Columbia, Canada Data type taxonomic summary and catalogue of occurrence records Brief description Curated taxonomic summary and catalogue of occurrence records documenting Actinopterygii reported for Galiano Island, British Columbia, Canada, in Darwin Core Standard format. File: oo_640879.xlsx https://binary.pensoft.net/file/640879 Antranig Basman, Charlie Gibbs, Donna Gibbs, Andy Lamb, Andrew Simon

8F3CCF1B-829D-54BA-B4A7-21994767AC05 10.3897/BDJ.10.e76050.suppl19 Supplementary material 19 Chondrichthyes of Galiano Island, British Columbia, Canada Data type taxonomic summary and catalogue of occurrence records Brief description Curated taxonomic summary and catalogue of occurrence records documenting Chondrichthyes reported for Galiano Island, British Columbia, Canada, in Darwin Core Standard format. File: oo_640885.xlsx https://binary.pensoft.net/file/640885 Antranig Basman, Charlie Gibbs, Donna Gibbs, Andy Lamb, Andrew Simon

8DCDC540-C15A-57A8-858C-A356D62E9DF3 10.3897/BDJ.10.e76050.suppl20 Supplementary material 20 Mammalia of Galiano Island, British Columbia, Canada Data type taxonomic summary and catalogue of occurrence records Brief description Curated taxonomic summary and catalogue of occurrence records documenting Mammalia reported for Galiano Island, British Columbia, Canada, in Darwin Core Standard format. File: oo_640889.xlsx https://binary.pensoft.net/file/640889 Antranig Basman, Charlie Gibbs, Donna Gibbs, Andy Lamb, Jessica Scott, Andrew Simon

## Figures and Tables

**Figure 2a.**
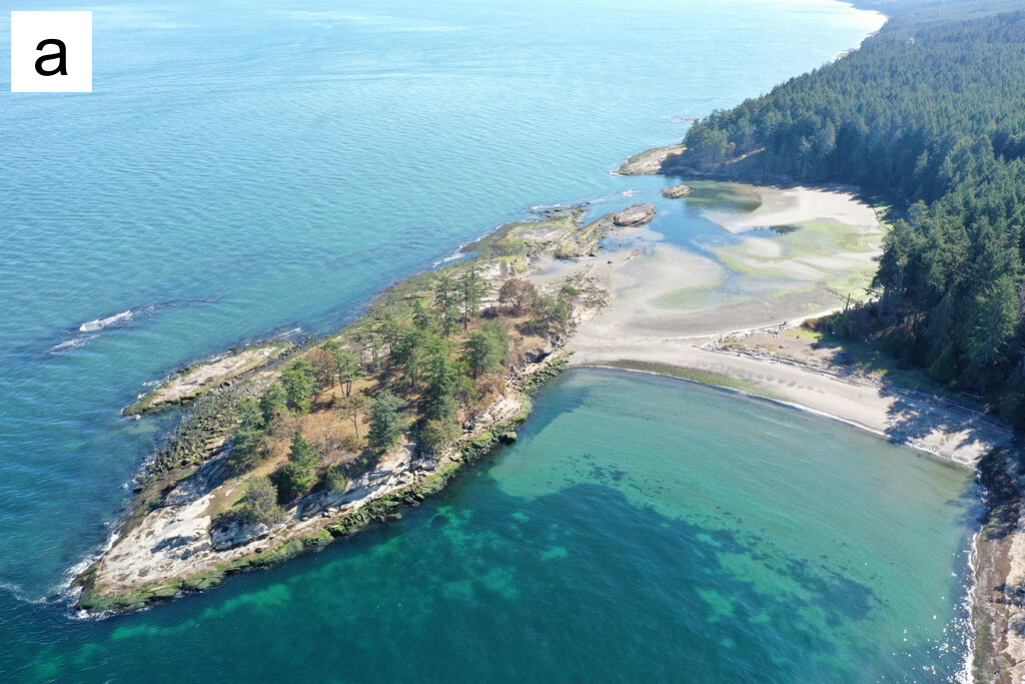
*Quelus* (Dionisio Point) exhibits a diversity of marine habitats, including subtidal eelgrass and kelp beds, sandy beach, tidal mudflats and rocky shoreline

**Figure 2b.**
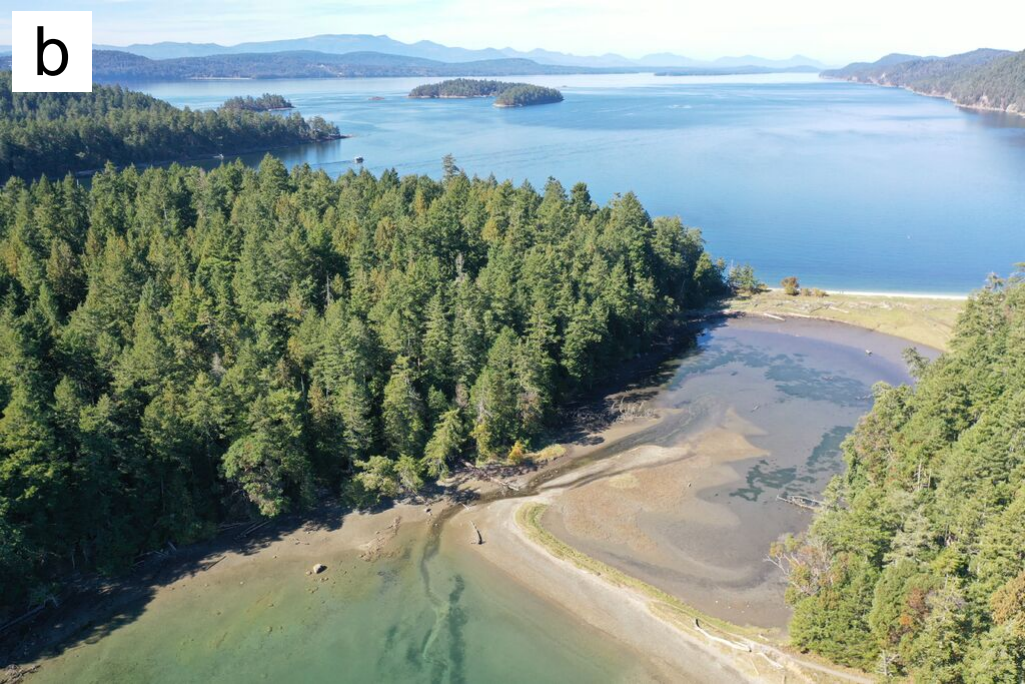
Marine habitat diversity at Sumnuw (Montague Harbour) includes shell beaches, tidal mudflats and salt marsh, the product of thousands of years of Indigenous landscape engineering

**Figure 2c.**
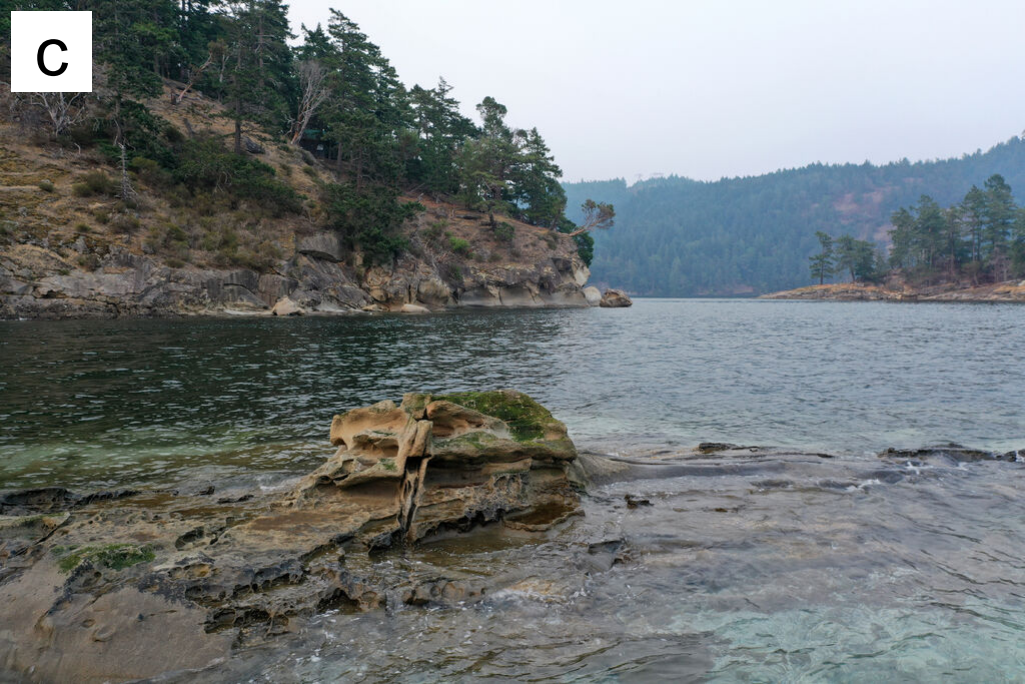
Dragon rock: a sandstone reef off the southwest coast of Parker Island, near Galiano Island

**Figure 2d.**
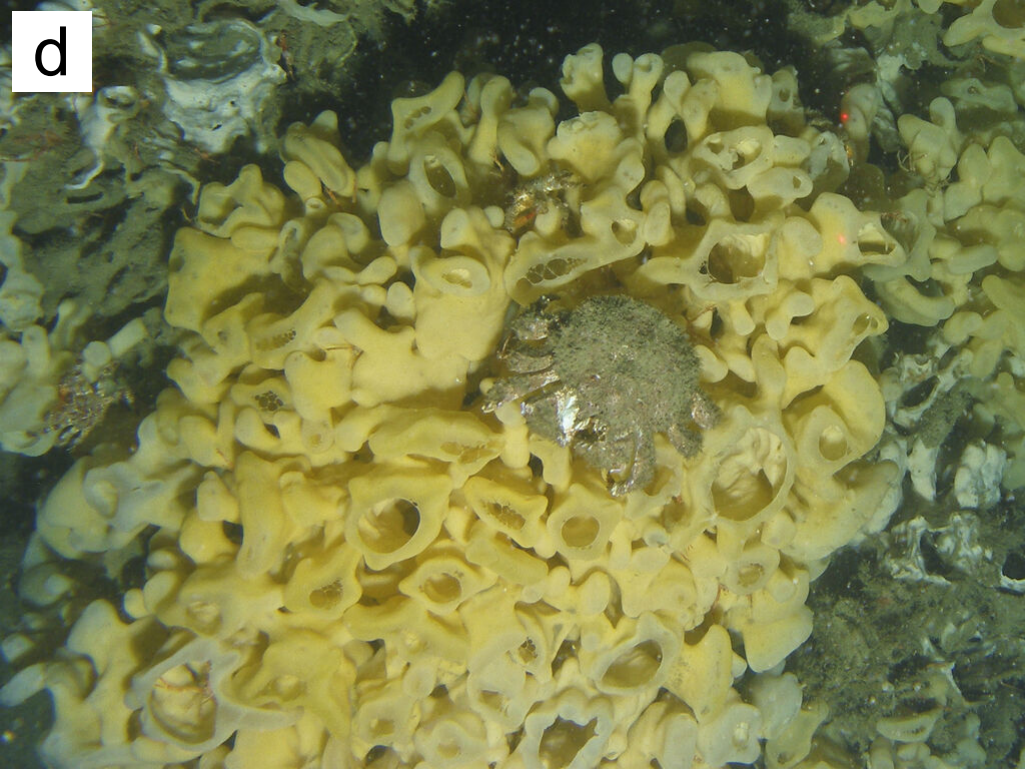
The cloud sponge (*Aphrocallistesvastus*) forms an extensive deep-sea reef off the southeast coast of Galiano Island, providing habitat for animals, such as the brown box crab (*Lopholithodesforaminatus*)

**Figure 2e.**
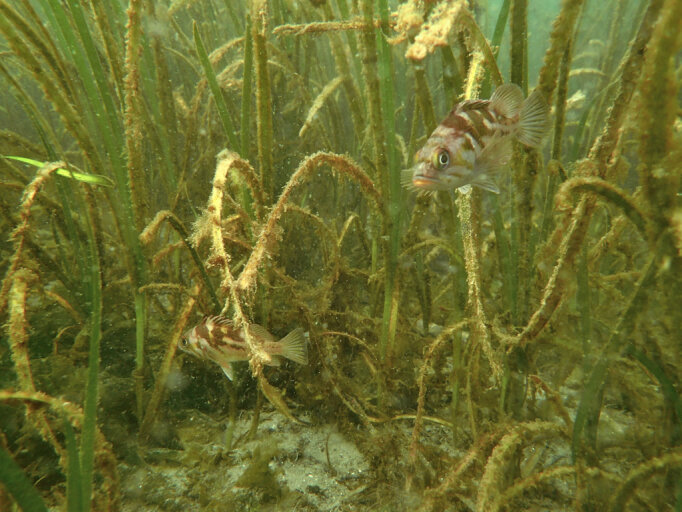
Juvenile copper rockfish (*Sebastescaurinus*) take refuge in an eelgrass meadow laden with diatoms, bacteria and other micro-organisms

**Figure 2f.**
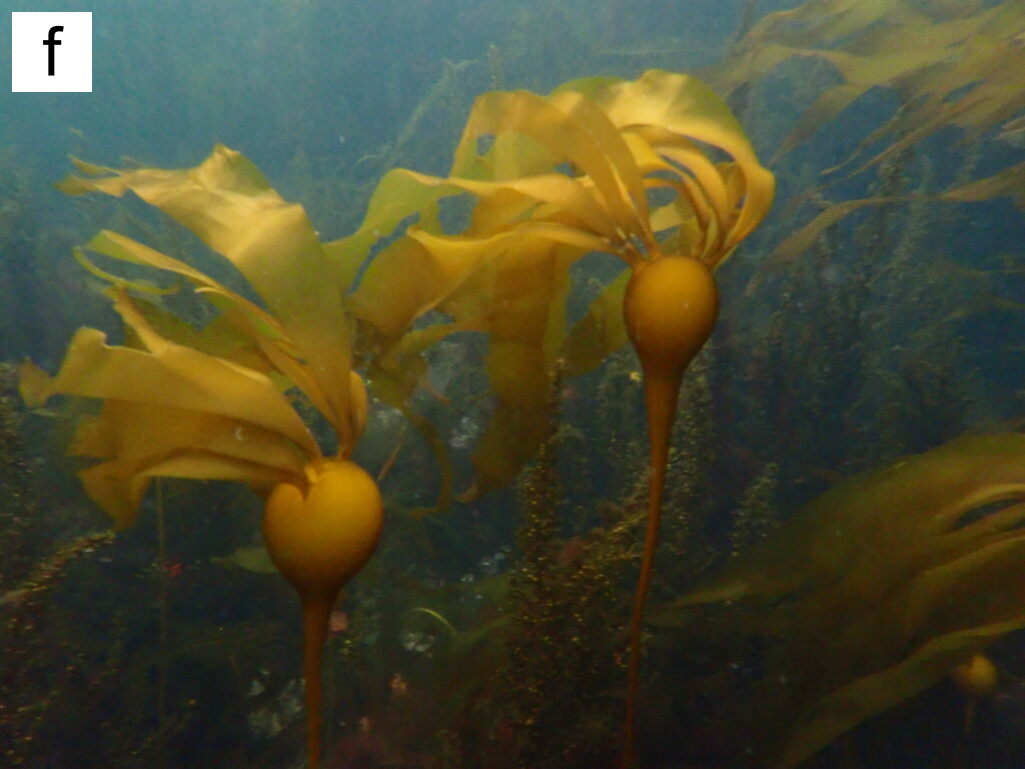
A bed of young bull kelp (*Nereocystisluetkeana*) interspersed with the introduced Japanese wireweed (*Sargassummuticum*) on a rocky shoreline nearby Collinson Point, Galiano Island.

**Table 1.** Summary of marine animal diversity reported for Galiano Island vis-à-vis global and regional biodiversity estimates. Regional diversity is estimated for the Pacific coast of North America (Pacific NA), the Northeast Pacific (NEP), coastal British Columbia (BC) and the Salish Sea, depending on the available data. See the taxonomic summaries below for sources of diversity estimates.

**Group**	**Global diversity**	**Regional diversity**	**Local diversity (Galiano Island)**	**Note**
Sponges (Porifera)	9,452 species	300–400 species (BC); 70 species (Salish Sea)	40 species	
Cnidarians (Cnidaria)	12,000 species	600 species (Pacific NA); 200 species (BC)	77 species	
Ctenophores (Ctenophora)	150–200 species	32 species (Pacific NA); 13 species (Salish Sea)*	4 species	*only 5 or 6 taxa commonly occur in the Salish Sea
Ribbon worms (Nemertea)	> 1,300 species	41 species (BC); 30 species (Salish Sea)	6 species	
Flatworms (Platyhelminthes)	13,000 marine species*	> 170 species (BC)	2 species	*this estimate does not account for cryptic parasitic species (e.g. Trematodes, (> 18,000 taxa)
Arrow worms (Chaetognatha)	130 species	6 species (NEP); 4 species (BC) 1–2 species (Salish Sea)	?*	*chaetognaths in the Galiano Island record remain undetermined to species
Molluscs (Mollusca)	49,000 species*	780 species (BC)	214 species	*global estimate incl. both terrestrial and marine spp; regional and local richness incl. only marine spp.
Ringed worms (Annelida)	> 13,000 species	> 450 species (BC); 860 species (Salish Sea)*	46 species	*known regional diversity vastly underestimates true richness
Peanut worms (Sipuncula)	150 species	8 species (NEP)	1 species	
Crustaceans (Crustacea)	52,000 species*	900 species (BC)	86 species	*global estimate incl. both terrestrial and marine spp; regional and local richness incl. only marine spp.
Nodding-heads (Entoprocta)	253 species	11 species (BC)	1 species	
Lampshells (Brachiopoda)	400 species	7 species (BC); 3 species (Salish Sea)	1 species	
Bryozoans (Bryozoa)	> 6,000 species	210–260 species (BC)	17 species	
Horseshoe worms (Phoronida)	13 species	6 species (NEP)	1 species	
Echinoderms (Echinodermata)	> 7,000 species	111 species (BC)	41 species	
Tunicates (Tunicata)	2,815 species	71 species (BC)	33 species	
Ray-finned and cartilaginous fish (Actinopterygii & Chondrichthyes)*	> 33,000 species > 1,100 species	> 1,500 species (Pacific NA); 400 species (BC)	82 species	*diversity estimates for Actinopterygii and Chondrichthyes are combined in regional and local summaries
Mammals (Mammalia)	6,400 species	22 species (Salish Sea)	13 species	
