# Supplementary material for: Toward an atlas of Salish Sea biodiversity: the flora and fauna of Galiano Island, British Columbia, Canada. Part I. Marine zoology
Source: Biodivers Data J. 2022 Mar 10;10:e76050. doi: 10.3897/BDJ.10.e76050 (PMC8930920; doi:10.3897/BDJ.10.e76050)
Supplement: Supplementary material 1 — Marine animals reported for Galiano Island, BC, Canada (1859–2021) [file bdj-10-e76050-s001.pdf]

Marine animals reported for Galiano Island, BC, Canada (1859–2021)

Curated by:

Emily Adamczyk, Antranig Basman, Jackson Chu, Karin Fletcher, Heidi Gartner, Charlie Gibbs, Donna Gibbs, Scott Gilmore, Rick Harbo, Leslie Harris, Elaine Humphrey, Andy Lamb, Philip Lambert, Neil McDaniel, Jessica Scott, and Andrew Simon

Based on records from:

Agassiz (1862), BC Cetacean Sightings Network, Biodiversity Galiano Project (iNaturalist), Canadian Museum of Nature, Chu & Leys (2010, 2012), Erickson (2000), McMurrich (1921), Pacific Marine Life Surveys, and the Royal British Columbia Museum. Occurrence records available at https://doi.org/10.15468/gv9cy5

Acknowledgments:

Sheila Byers, Jim Carlton, Henry Choong, Roger Clark, Keith Erickson, Doug Ernisse, Bernie Hanby, Gregory Jensen, Gretchen Lambert, Sandra Lindstrom, Milton Love, Jeff Marliave, Claudia Mills, Bruce Ott, Wyatt Patry, Amy Rowley, Linda Schroeder, Mary Wicksten, and Gary Williams

Contact:

Andrew Simon – adfsimon@imerss.org
Antranig Basman – amb26@ponder.org.uk

Animalia

Porifera – sponges

Calcarea

Clathrinida

Clathrinidae

***Clathrina* sp.** - lattice-skin sponge

Leucosolenida (order)

Amphoriscidae

Leucilla nuttingi (Urban, 1902) - stalked vase sponge

Grantiidae

Leucandra heathi Urban, 1906 - bristly vase sponge

Leucosoleniidae

Leucosolenia eleanor Urban, 1906 - spaghetti sponge

Leucosolenia nautilia de Laubenfels, 1930 - lacy ball sponge

SYCETTIDAE

***Sycon* spp.** - tiny vase sponge

Demospongiae

Clionaida

CLIONAIDAE

Cliona californiana de Laubenfels, 1932 - yellow boring sponge

Dendroceratida

DARWINELLIDAE

Aplysilla polyraphis de Laubenfels, 1930 - slippery purple sponge

***Aplysilla* sp.** - slippery rose sponge

Desmacellida

DESMACELLIDAE

Desmacella austini Lehnert, Conway, Barrie & Krautter, 2005 - thin encrusting Desmacella

Dictyoceratida

DYSIDEIDAE

***Pleraplysilla* sp.** - slippery white sponge

Haplosclerida

CHALINIDAE

***Haliclona (Haliclona)* sp.1** - gnarled finger sponge

***Haliclona (Haliclona)* sp.2** - pale orange carpet sponge

NIPHATIDAE

***Pachychalina* sp.** - thick-strap sponges

PETROSIIDAE

Xestospongia hispida (Ridley & Dendy, 1886) - hard gnarled clump sponge

Poecilosclerida

ACARNIDAE

Acarnus erithacus de Laubenfels, 1927 - thick encrusting scarlet sponge

Iophon lamella Wilson, 1904 - white reticulated sponge

ESPERIOPSIDAE

Amphilectus digitatus (Miklucho-Maclay, 1870) - glove sponge

Semisuberites cribrosa (Miklucho-Maclay, 1870) - funnel sponge

ISODICTYIDAE

Isodictya rigida (Lambe, 1893) - orange finger sponge

HYMEDESMIIDAE

***Hamigera* sp.** - orange cratered encrusting sponge

MICROCIONIDAE

Antho (Plocamia) karykina (de Laubenfels, 1927) - bright red sponge

Clathria (Microciona) pennata (Lambe, 1895) - red velvety sponge

MYCALIDAE

Mycale (Aegogropila) adhaerens (Lambe, 1893) - smooth scallop sponge

MYXILLIDAE

Myxilla (Myxilla) incrustans (Johnston, 1842) - rough scallop sponge

Myxilla (Myxilla) lacunosa Lambe, 1893 - sulphur sponge

Polymastiida

POLYMASTIIDAE

Polymastia pachymastia de Laubenfels, 1932 - aggregated nipple sponge

Weberella perlucida Austin, Ott, Reiswig, Romagosa & McDaniel, 2014 - retractable nipple

Suberitida

HALICHONDRIIDAE

Halichondria (Eumastia) sitiens (Schmidt, 1870) - breast sponge

Halichondria (Halichondria) bowerbanki Burton, 1930 - Bowerbank's crumb of bread sponge

Halichondria (Halichondria) panicea (Pallas, 1766) - yellow-green encrusting sponge

SUBERITIDAE

Homaxinella amphispicula (de Laubenfels, 1961) - tough yellow branching sponge

Suberites concinnus Lambe, 1895 - peach ball sponge

Suberites latus Lambe, 1893 - hermit crab sponge

Tethyida

TETHYIDAE

Tethya californiana de Laubenfels, 1932 - orange rough ball sponge

Tetractinellida

TETILLIDAE

Craniella villosa Lambe, 1893 - tennis ball sponge

Tetilla sp.

Hexactinellida

Lyssacinosida

ROSSELLIDAE

Rhabdocalyptus dawsoni (Lambe, 1893) - boot sponge

Sceptrulophora

APHROCALLISTIDAE

Aphrocallistes vastus Schulze, 1886 - cloud sponge

Heterochone calyx (Schulze, 1886) - fingered goblet sponge

CNIDARIA – CNIDARIANS

Anthozoa

Actiniaria

ACTINIIDAE

Anthopleura artemisia (Pickering in Dana, 1846) - burrowing anemone

Anthopleura elegantissima (Brandt, 1835) - pink-tipped anemone

Cribrinopsis fernaldi Siebert & Spaulding, 1976 - snakelock anemone

Cribrinopsis rubens Sanamyan, Sanamyan, McDaniel, Martynov, Korshunova & Bocharova, 2019

Epiactis lisbethae Fautin & Chia, 1986 - brooding anemone

Epiactis prolifera Verrill, 1869 - proliferating anemone

Urticina clandestina Sanamyan N., Sanamyan K. & McDaniel, 2013 - stubby rose anemone

Urticina grebelnyi Sanamyan & Sanamyan, 2006 - painted anemone

Actinostolidae

Stomphia coccinea (Müller, 1776) - spotted swimming anemone

Stomphia didemon Siebert, 1973 - swimming anemone

Diadumenidae

Diadumene lineata (Verrill, 1869) - striped green sea anemone

Halcampidae

Halcampa decemtentaculata Hand, 1955 - ten-tentacled burrowing anemone

Haloclavidae

Peachia quinquecapitata McMurrich, 1913 - jelly-dwelling anemone

Metridiidae

Metridium farcimen (Brandt, 1835) - giant plumose anemone

Metridium senile (Linnaeus, 1761) - short plumose anemone

Alcyonacea

Alcyoniidae

***Alcyonium* sp.** - orange soft coral

Discophyton rudyi (Verseveldt & van Ofwegen, 1992) - white soft coral

Thrombophyton trachydermum McFadden & Hochberg, 2003 - pale soft coral

Pennatulacea

Balticinidae

Balticina willemoesi (Kölliker, 1880) - sea whip

Pennatulidae

Ptilosarcus gurneyi (Gray, 1860) - orange sea pen

Virgulariidae

Stylatula elongata (Gabb, 1862) - spiny white sea pen

***Virgularia* sp.** - white sea pen

Scleractinia

Caryophylliidae

Caryophyllia (Caryophyllia) alaskensis Vaughan, 1941 - round tan cup coral

Paracyathus stearnsii Verrill, 1869 - brown oval cup coral

Dendrophylliidae

Balanophyllia (Balanophyllia) elegans Verrill, 1864 - orange cup coral

Spirularia

Cerianthidae

Pachycerianthus fimbriatus McMurrich, 1910 - tube-dwelling anemone

Zoantharia (order)

Epizoanthidae

Epizoanthus scotinus Wood, 1957 - orange zoanthid

Hydrozoa

Anthoathecata

Bougainvilliidae

***Bougainvillia* sp.** - cluster-tentacled jelly

Garveia annulata Nutting, 1901 - orange hydroid

Corymorphidae

Euphysa spp. - tiny red sausage jellies

Corynidae

Polyorchis penicillatus (Eschscholtz, 1829) - red-eye medusa

Sarsia spp. - thimble jellyfishes

Sarsia tubulosa (M. Sars, 1835) - clapper hydroid

Hydractiniidae

Hydractinia sp. - barnacle fur hydroid

Schuchertinia allmanii (Bonnevie, 1898) - snail fur hydroid

Schuchertinia milleri (Torrey, 1902) - hedge-hog hydroid

Pandeidae

Catablema vesicarium nodulosum (A. Agassiz, 1862)

Leuckartiara longicalcar Schuchert, 2018

Neoturris breviconis (Murbach & Shearer, 1902) - blob-top jelly

Stomotoca atra L. Agassiz, 1862 - hanging stomach jelly

Similiclavidae

Similiclava nivea Calder, Choong & McDaniel, 2015 - white hydroid

Stylasteridae

Stylantheca papillosa (Dall, 1884) - encrusting hydrocoral

Stylaster verrillii (Dall, 1884)

Tubulariidae

Ectopleura crocea (Agassiz, 1862) - bushy pink-mouth hydroid

Ectopleura marina (Torrey, 1902) - solitary pink-mouth hydroid

Tubularia indivisa Linnaeus, 1758 - giant pink-mouth hydroid

Leptothecata

Aequoreidae

Aequorea victoria s.lat. - water jelly

Aglaopheniidae

Aglaophenia spp. - ostrich-plume hydroid

Campanulariidae

Clytia gregaria (Agassiz, 1862) - gregarious jellyfish

Obelia spp. - wine-glass hydroid

Orthopyxis sp. - spider web hydroid

Rhizocaulus verticillatus (Linnaeus, 1758) - horse-tail hydroid

Eirenidae

Eutonina indicans (Romanes, 1876) - aggregating jelly

Haleciidae

Halecium beanii (Johnston, 1838) - candalabrum hydroid

Halecium densum Calkins, 1899 - dense bushy hydroid

Lafoeidae

Grammaria spp. - spindly embedded hydroid

Lafoea dumosa (Fleming, 1820) - muff hydroid

Melicertidae

Melicertum octocostatum (M. Sars, 1835) - eight-strand jelly

MITROCOMIDAE

Mitrocoma cellularia (Agassiz, 1862) - cross jellyfish

PLUMULARIIDAE

Plumularia setacea (Linnaeus, 1758) - glassy plume hydroid

***Plumularia* sp.** - delicate plume hydroid

SERTULARELLIDAE

***Sertularella* sp.** - garland hydroid

SERTULARIIDAE

Amphisbetia greenei (Murray, 1860) - fibre-optic hydroid

***Abietinaria* spp.** - coarse sea fir hydroids

Hydrallmania franciscana (Trask, 1857) - loose spiral hydroid

***Thuiaria* sp.1** - fish-bone hydroid

***Thuiaria* spp.** - embedded sea fir hydroids

Thuiaria thuja (Linnaeus, 1758) - bottlebrush hydroid

Limnomedusae

OLINDIIDAE

Gonionemus vertens A. Agassiz, 1862 - clinging jellyfish

Narcomedusae

CUNINIDAE

***Solmissus* spp.** - scalloped jelly

SOLMUNDAEGINIDAE

Solmundaegina nematophora Lindsay, 2017 - four tentacled jelly

Siphonophorae

AGALMATIDAE

Nanomia bijuga (Delle Chiaje, 1844) - tailed jelly

DIPHYIDAE

***Muggiaea* sp.** - dwarf tailed jelly

Trachymedusae

RHOPALONEMATIDAE

Aglantha digitale (O. F. Müller, 1776) - pink helmit

Scyphozoa

Semaeostomeae

CYANEIDAE

Cyanea ferruginea Eschscholtz, 1829 - lion's mane

PHACELLOPHORIDAE

Phacellophora camtschatica Brandt, 1835 - fried egg jellyfish

ULMARIDAE

Aurelia labiata Chamisso & Eysenhardt, 1821 - moon jelly

Ctenophora – ctenophores

Nuda

Beroida

Beroidae

Beroe abyssicola Mortensen, 1927 - transluscent comb jelly

Tentaculata

Cydippida

Euplokamididae

Euplokamis dunlapae Mills, 1987 - oval sea gooseberry

Pleurobrachiidae

Pleurobrachia bachei A. Agassiz, 1860 - sea gooseberry

Lobata

Bolinopsidae

Bolinopsis infundibulum (O.F. Müller, 1776) - lobed sea gooseberry

Nemertea – ribbon worms

Hoplonemertea

Monostilifera

Emplectonematidae

Emplectonema gracile (Johnston, 1837) - green ribbon worm

Neesiidae

Paranemertes peregrina Coe, 1901 - purple ribbon worm

Palaeonemertea

Tubulaniformes

Tubulanidae

Tubulanus albocinctus (Coe, 1904) - white-ringed ribbon worm
Tubulanus polymorphus Renier, 1804 - orange ribbon worm
Tubulanus sexlineatus (Griffin, 1898) - six-lined ribbon worm

Pilidiophora

Heteronemertea

Lineidae

Micrura verrilli Coe, 1901 - purple ribbon worm

Platyhelminthes – flatworms

Rhabditophora

Polycladida

Callioplanidae

Kaburakia excelsa Bock, 1925 - giant flatworm

Euryleptidae

Eurylepta leoparda Freeman, 1933 - spotted flatworm

Chaetognatha – arrow worms

Sagittoidea

Mollusca – molluscs

Bivalvia

Adapedonta

Hiatellidae

Panomya ampla Dall, 1898 - ample roughmya
Panopea generosa Gould, 1850 - Pacific geoduck

Solenidae

Solen sicarius Gould, 1850 - sickle jackknife-clam

Arcida

Glycymerididae

Glycymeris septentrionalis (Middendorff, 1849) - western bittersweet

Cardiida

Cardiidae

Ciliatocardium ciliatum (Fabricius, 1780) - hairy cockle
Clinocardium nuttallii (Conrad, 1837) - Nuttall's cockle
Keenaea centifilosa (Carpenter, 1864) - cockle
Keenocardium blandum (Gould, 1850)

Psammobiidae

Gari californica (Conrad, 1849) - California sunsetclam
Nuttallia obscurata (Reeve, 1857) - purple mahogany clam

Semelidae

Semele rubropicta Dall, 1871 - rose-painted clam

Tellinidae

Macoma brota Dall, 1916 - heavy macoma
Macoma calcarea (Gmelin, 1791) - chalky macoma
Macoma carlottensis Whiteaves, 1880 - Charlotte macoma
Macoma nasuta (Conrad, 1837) - bent-nose macoma
Macoma obliqua (J. Sowerby, 1817)

Carditida

Carditidae

Coanicardita ventricosa (Gould, 1850)

Galeommatida

Lasaeidae

Kellia suborbicularis (Montagu, 1803) - suborbicular kellyclam

Lucinida

Lucinidae

Parvilucina tenuisculpta (Carpenter, 1864)

THYASIRIDAE

Axinopsida serricata (Carpenter, 1864) - lenticular axinopsid

Myida

MYIDAE

Mya arenaria Linnaeus, 1758 - soft-shelled clam

Mya truncata Linnaeus, 1758 - truncated softshell-clam

PHOLADIDAE

Zirfaea pilsbryi H. N. Lowe, 1931 - rough piddock

TEREDINIDAE

Bankia setacea (Tryon, 1863) - feathery shipworm

Mytilida

MYTILIDAE

Crenella decussata (Montagu, 1808)

Modiolus modiolus (Linnaeus, 1758) - northern horsemussel

Modiolus rectus (Conrad, 1837)

Musculus discors (Linnaeus, 1767) - discord mussel

Musculus niger (J.E. Gray, 1824) - black mussel

***Mytilus edulis* complex** - blue mussel

Nuculanida

NUCULANIDAE

Nuculana leonina (Dall, 1896) - nuculanid clam

YOLDIIDAE

Megayoldia thraciaeformis (Storer, 1838) - broad yoldia

Yoldia aeolica (Valenciennes, 1846) - yoldia clam

Yoldia hyperborea (Gould, 1841) - yoldia clam

Nuculida

NUCULIDAE

Acila castrensis (Hinds, 1843) - nutclam or nutshell

Ennucula tenuis (Montagu, 1808) - smooth nutclam

Ostreida

OSTREIDAE

Magallana gigas (Thunberg, 1793) - Pacific oyster

PANDOROIDEA

Lyonsiidae

Entodesma navicula (Adams & Reeve, 1850) - rock entodesma

Lyonsia bracteata (Gould, 1850) - scaly lyonsia

Mytilimeria nuttalli Conrad, 1837 - bladderclam

PANDORIDAE

Pandora filosa (Carpenter, 1864) - threaded pandora

Pandora wardiana A. Adams, 1860 - giant pandora

Pectinida

ANOMIIDAE

Pododesmus macrochisma (Deshayes, 1839) - green false-jingle

Pectinidae

Chlamys hastata (G. B. Sowerby II, 1842) - spiny pink scallop
Chlamys rubida (Hinds, 1845) - smooth pink scallop
Crassadoma gigantea (J.E. Gray, 1825) - giant rock scallop
Patinopecten caurinus (Gould, 1850) - giant Pacific sea scallop

Thracioidea

Thraciidae

Thracia trapezoides Conrad, 1849 - trapezoid thracia

Venerida

Mactridae

Tresus capax (Gould, 1850) - fat gaper
Tresus nuttallii (Conrad, 1837) - Pacific gaper

Ungulinidae

Zemysina impolita (S. S. Berry, 1953) - rough diplodon

Veneridae

Compsomyax subdiaphana (Carpenter, 1864) - milky venus
Humilaria kennerleyi (Reeve, 1863) - Kennerley's venus
Leukoma staminea (Conrad, 1837) - Pacific littleneck clam
Ruditapes philippinarum (A. Adams & Reeve, 1850) - Japanese littleneck
Saxidomus gigantea (Deshayes, 1839) - Washington butter clam

Cephalopoda

Myopsida

Loliginidae

Doryteuthis opalescens (Berry, 1911) - California market squid

Octopoda

Enteroctopodidae

Enteroctopus dofleini (Wülker, 1910) - giant pacific octopus

Octopodidae

Octopus rubescens Berry, 1953 - Pacific red octopus

Oegopsida

Gonatidae

Berryteuthis magister (Berry, 1913) - schoolmaster armhook squid

Sepiida

Sepiolidae

Rossia pacifica Berry, 1911 - stubby squid

Gastropoda

Acteonoidea

Acteonidae

Rictaxis punctocaelatus (Carpenter, 1864) - bubble snail

Aplysiida

Aplysiidae

Phyllaplysia taylori Dall, 1900 - zebra leafslug

Cephalaspidea

Aglajidae

Aglaja ocelligera (Bergh, 1894) - spotted aglaja

Melanochlamys diomedea (Bergh, 1894) - Diomedes aglajid

Cylichnidae

Cylichna attonsa Carpenter, 1865 - deep trouble chalice-bubble

Gastropteridae

Gastropteron pacificum Bergh, 1894 - winged sea slug

Haminoeidae

Haminoea vesicula (Gould, 1855) - white bubble shell

Tornatinidae

Acteocina culcitella (Gould, 1853) - pillow barrel-bubble

Cerithioidea

Batillariidae

Batillaria attramentaria (G. B. Sowerby II, 1855) - Japanese false cerith

Cerithiidae

Neostylidium eschrichtii (Middendorff, 1849) - threaded bittium

Ellobiida

Ellobiidae

Myosotella myosotis (Draparnaud, 1801) - mouse-eared snail

Epitonioidea

Epitoniidae

Epitonium hindsii (Carpenter, 1856)

Epitonium indianorum (Carpenter, 1865) - money wentletrap

Lepetellida

Fissurellidae

Diodora aspera (Rathke, 1833) - rough keyhole limpet

Puncturella cucullata (Gould, 1846) - hooded puncturella

Haliotidae

Haliotis kamtschatkana Jonas, 1845 - northern abalone

Littorinimorpha

Calyptraeidae

Crepidula* cf *nummaria - northern white slippersnail

Crepidula norrisiarum Williamson, 1905 - hooked slippersnail

Capulidae

Trichotropis cancellata Hinds, 1843 - checkered hairysnail

Cymatiidae

Fusitriton oregonensis (Redfield, 1846) - Oregon triton

Eulimidae

Melanella micans (Carpenter, 1865) - shining balcis

Littorinidae

Lacuna variegata Carpenter, 1864 - variable lacuna

Littorina scutulata Gould, 1849 - checkered periwinkle
Littorina sitkana Philippi, 1846 - Sitka periwinkle

LOTTIOIDEA

ACMAEIDAE

Acmaea mitra Rathke, 1833 - whitecap limpet

LEPETIDAE

Lepeta concentrica (Middendorff, 1848) - ringed blind limpet

LOTTIIDAE

Lottia digitalis (Rathke, 1833) - fingered limpet
Lottia pelta (Rathke, 1833) - shield limpet
Lottia persona (Rathke, 1833) - mask limpet
Lottia scutum (Rathke, 1833) - plate limpet

NATICIDAE

Cryptonatica aleutica (Dall, 1919) - Aleutian moonsnail
Euspira pallida (Broderip & G. B. Sowerby I, 1829) - pale northern moonsnail
Neverita lewisii (Gould, 1847) - Lewis' moon snail

VELUTINIDAE

Marsenina rhombica (Dall, 1871) - marbled lamellarid
Marsenina stearnsii (Dall, 1871) - stearn's ear shell
Velutina plicatilis (O. F. Müller, 1776) - oblique lamellaria
Velutina velutina (O. F. Müller, 1776) - spiral velvet snail

VERMETIDAE

Petaloconchus* cf *compactus - northern compact wormsnail

Neogastropoda

BORSONIIDAE

Ophiodermella fancherae (Dall, 1903) - sea snail

COLUMBELLIDAE

Alia carinata (Hinds, 1844) - carinate dovesnail
Amphissa columbiana Dall, 1916 - wrinkled amphissa
Amphissa versicolor Dall, 1871 - variegated amphissa
Mitrella gausapata (Gould, 1850) - sea snail
***Nitidella* sp.**

FASCIOLARIIDAE

***Fusinus* sp.**

MANGELIIDAE

***Oenopota* sp.**

MURICIDAE

Ceratostoma foliatum (Gmelin, 1791) - leafy hornmouth
Nucella canaliculata (Duclos, 1832) - channelled dogwinkle
Nucella lamellosa (Gmelin, 1791) - wrinkled dogwinkle
Nucella ostrina (Gould, 1852) - northern striped dogwinkle
Paciocinebrina interfossa (Carpenter, 1864) - sculptured rocksnail
Paciocinebrina lurida (Middendorff, 1848) - lurid rocksnail
Scabrotrophon maltzani (Kobelt, 1878) - sandpaper trophon

Trophonopsis orpheus (Gould, 1849) - orpheus trophon

NASSARIIDAE

Nassarius mendicus (Gould, 1850) - western lean nassa

TUDICLIDAE

Lirabuccinum dirum (Reeve, 1846) - dire whelk

Nudibranchia

ACTINOCYCLIDAE

Hallaxa chani Gosliner & G. C. Williams, 1975 - Chan's dorid

AEGIRIDAE

Aegires albopunctatus MacFarland, 1905 - salt-and-pepper nudibranch

AEOLIDIIDAE

Aeolidia loui Kienberger, Carmona, Pola, Padula, Gosliner & Cervera, 2016

Aeolidia papillosa (Linnaeus, 1761) - papillate sea slug

Cerberilla mosslandica McDonald & Nybakken, 1975 - brown burrowing aeolid

APATAIDAE

Apata pricei (MacFarland, 1966) - Price's aeolid

ARMINIDAE

Armina californica (J. G. Cooper, 1863) - striped nudibranch

CADLINIDAE

Aldisa albomarginata Millen in Millen & Gosliner, 1985 - white-rimmed nudibranch

Aldisa cooperi Robilliard & Baba, 1972 - Cooper's dorid

Aldisa sanguinea (J. G. Cooper, 1863) - blood-spot dorid

Aldisa tara Millen in Millen & Gosliner, 1985 - Tara's dorid

Cadlina klasmalmbergi Korshunova, Fletcher et al., 2020 - Klas' yellow-rimmed nudibranch

Cadlina luteomarginata MacFarland, 1966 - yellow-rimmed nudibranch

Cadlina modesta MacFarland, 1966 - modest cadlina

CALYCIDORIDIDAE

Diaphorodoris lirulatocauda Millen, 1985 - porcupine dorid

CORYPHELLIDAE

Coryphella verrucosa (M. Sars, 1829) - red-gilled nudibranch

Himatina trophina (Bergh, 1890) - red flabellina

Orienthella trilineata (O'Donoghue, 1921) - three-lined nudibranch

CUMANOTIDAE

Cumanotus fernaldi T. E. Thompson & G. H. Brown, 1984 - Fernald's aeolid

CUTHONELLIDAE

Cuthonella concinna (Alder & Hancock, 1843) - neat aeolid

CUTHONIDAE

Cuthona divae (Er. Marcus, 1961) - rose-pink cuthona

DENDRONOTIDAE

Dendronotus albopunctatus Robilliard, 1972 - white-spotted dendronotid

Dendronotus albus MacFarland, 1966 - white dendronotid

Dendronotus iris J. G. Cooper, 1863 - giant nudibranch

Dendronotus kamchaticus Ekimova et al., 2015 - white-tipped dendronotus

Dendronotus robilliardi Korshunova et al., 2016 - Robilliard's dendronotus
Dendronotus rufus O'Donoghue, 1921 - red dendronotid
Dendronotus subramosus MacFarland, 1966 - stubby-frond dendronotus
Dendronotus venustus MacFarland, 1966 - branched dendronotus

DIRONIDAE

Dirona albolineata MacFarland, 1905 - frosted nudibranch
Dirona pellucida Volodchenko, 1941 - golden dirona

DISCODORIDIDAE

Diaulula odonoghuei (Steinberg, 1963) - spotted leopard dorid
Geitodoris heathi (MacFarland, 1905) - Heath's dorid
Peltodoris lentiginosa (Millen, 1982) - freckled pale sea lemon
Peltodoris nobilis (MacFarland, 1905) - noble sea lemon
Rostanga pulchra MacFarland, 1905 - red sponge nudibranch

DORIDIDAE

Doris montereyensis J. G. Cooper, 1863 - Monterey sea lemon
Doris odhneri MacFarland, 1966 - white nudibranch

DOTIDAE

Doto amyra Er. Marcus, 1961 - hammerhead doto
Doto columbiana O'Donoghue, 1921 - British Columbia doto

EUBRANCHIDAE

Eubranchus rupium (Møller, 1842) - olive aeolid
Eubranchus rustyus (Er. Marcus, 1961) - homely aeolid
Eubranchus sanjuanensis Roller, 1972 - San Juan aeolid

JANOLIDAE

Antiopella fusca (O'Donoghue, 1924) - white-and-orange-tipped nudibranch

MYRRHINIDAE

Hermissenda crassicornis (Eschscholtz, 1831) - thick-horned nudibranch

ONCHIDORIDIDAE

Acanthodoris atrogriseata O'Donoghue, 1927 - gray pilose dorid
Acanthodoris hudsoni MacFarland, 1905 - Hudson's dorid
Acanthodoris lutea MacFarland, 1925 - sandalwood dorid
Acanthodoris nanaimoensis O'Donoghue, 1921 - Nanaimo nudibranch
Adalaria proxima (Alder & Hancock, 1854) - white adalaria
Knoutsodonta jannae (Millen, 1987) - Janna's adalaria
Onchidoris bilamellata (Linnaeus, 1767) - barnacle-eating nudibranch
Onchidoris evincta (Millen, 2006) - crowned dorid
Onchidoris muricata (O. F. Müller, 1776) - fuzzy onchidoris

PARACORYPHELLIDAE

Ziminella japonica (Volodchenko, 1941) - pearly nudibranch

POLYCERIDAE

Limacia cockerelli (MacFarland, 1905) - Cockerell's nudibranch
Palio zosterae (O'Donoghue, 1924) - eelgrass nudibranch
Triopha modesta Bergh, 1880 - modest clown nudibranch

TETHYDIDAE

Melibe leonina (Gould, 1852) - hooded nudibranch

TRINCHESIIDAE

Catriona columbiana (O'Donoghue, 1922) - British Columbia aeolid

Diaphoreolis viridis (Forbes, 1840) - green aeolid

Zelentia nepunicea Korshunova, Fletcher, Lundin, Picton & Martynov, 2018 - pimpled aeolid

TRITONIIDAE

Tochuina gigantea (Bergh, 1904) - orange-peel nudibranch

Tritonia exsulans Bergh, 1894 - pink tritonia

Tritonia festiva (Stearns, 1873) - diamondback tritonia

Pleurobranchida

PLEUROBRANCHIDAE

Berthella chacei (J. Q. Burch, 1944) - white berthella

Pteropoda

CLIONIDAE

Clione limacina (Phipps, 1774) - common clione

LIMACINIDAE

Limacina helicina (Phipps, 1774) - helicid pteropod

PYRAMIDELLOIDEA

PYRAMIDELLIDAE

***Odostomia* sp.** - sea snail

***Turbonilla* sp.** - sea snail

Trochida

CALLIOSTOMATIDAE

Calliostoma annulatum (Lightfoot, 1786) - purple-ringed topsnail

Calliostoma ligatum (Gould, 1849) - blue topsnail

MARGARITIDAE

Margarites pupillus (Gould, 1849) - puppet margarite

TEGULIDAE

Tegula funebralis (A. Adams, 1855) - black tegula

Polyplacophora

Chitonida

ACANTHOCHITONIDAE

Cryptochiton stelleri (Middendorff, 1847) - giant Pacific chiton

ISCHNOCHITONIDAE

Lepidozona interstincta (Gould, 1852) - smooth lepidozona

Lepidozona mertensii (Middendorff, 1847) - Merten's chiton

Tripoplax trifida (Carpenter, 1864) - three-rib chiton

MOPALIIDAE

Katharina tunicata (Wood, 1815) - black leather chiton

Mopalia ciliata (G. B. Sowerby II, 1840) - northern hairy chiton

Mopalia hindsii (Reeve, 1847) - Hind's mopalia

Mopalia lignosa (Gould, 1846) - woody chiton

Mopalia muscosa (Gould, 1846) - mossy chiton

Mopalia phorminx Berry, 1919 - feathery mopalia
Mopalia sinuata Carpenter, 1864 - dwarf hairy mopalia
Mopalia spectabilis I. M. Cowan & G. M. Cowan, 1977 - red-flecked mopalia
Mopalia swanii Carpenter, 1864 - Swan's mopalia
Mopalia vespertina (Gould, 1852) - smooth mopalia
Placiphorella rufa Berry, 1917 - red veiled-chiton
Placiphorella velata (Carpenter MS, Dall, 1879) - veiled-chiton

TONICELLIDAE

Cyanoplax dentiens (Gould, 1846) - Gould's baby chiton
***Lepidochitona* sp.**
Tonicella insignis (Reeve, 1847) - white-line chiton
Tonicella lineata (Wood, 1815) - lined chiton
Tonicella* cf *undocaerulea - blue lined chiton

Scaphopoda

Dentaliida

RHABDIDAE

Rhabdus rectius (Carpenter, 1864) - straight tuskshell

Gadilida

GADILIDAE

Gadila aberrans (Whiteaves, 1887) - aberrant toothshell

ANNELIDA – ANNELIDS

Clitellata

Tubificida

NAIDIDAE

Bathydrilus litoreus Baker, 1983 - naidid worm
Limnodriloides victoriensis Brinkhurst & Baker, 1979 - naidid worm

Polychaeta

Eunicida

ONUPHIDAE

Diopatra ornata Moore, 1911 - ornate tubeworm

Phyllodocida

APHRODITIDAE

Aphrodita negligens Moore, 1905 - sea mouse

HESIONIDAE

Oxydromus pugettensis (Johnson, 1901) - bat star commensal worm

NEREIDIDAE

Alitta williami Villalobos-Guerrero & Bakken, 2018 - giant piling sea-nymph worm
Nereis* cf *zonata - ragworm
***Nereis* sp.** - sea-nymph worm
Nereis vexillosa Grube, 1851 - banner sea-nymph
Platynereis bicanaliculata (Baird, 1863) - algae-dwelling sea-nymph

Phyllodocidae

***Eulalia* sp.**

Polynoidae

Arctonoe fragilis (Baird, 1863) - fragile ruffled scaleworm
Arctonoe pulchra (Johnson, 1897) - red commensal scaleworm
Arctonoe vittata (Grube, 1855) - red-banded commensal scaleworm
Halosydna brevisetosa Kinberg, 1856 - eighteen-scaled worm
Harmothoe fragilis Moore, 1910 - chocolate & white scaleworm

Syllidae

Epigamia* cf *alexandri - noble necklace worm
Trypanedenta gemmipara (Johnson, 1901) - brown jewel necklace worm
***Trypanosyllis* sp.** - necklace-worm

Tomopteridae

***Tomopteris* sp.**

Sabellida

Sabellariidae

Neosabellaria cementarium (Moore, 1906) - cemented sandmason tubeworm

Sabellidae

Bispira pacifica (Berkeley & Berkeley, 1954) - twin-eyed feather duster
Chone aurantiaca (Johnson, 1901) - orange feather-duster
Eudistylia vancouveri (Kinberg, 1866) - Vancouver feather-duster
Myxicola* aff. *aesthetica - petite slime-tube feather-duster
Myxicola* aff. *infundibulum - slime-tube feather-duster
Parasabella media Bush, 1905 - parasol feather-duster
Pseudopotamilla occelata Moore, 1905 - feather-duster
Schizobranchia insignis Bush, 1905 - split-branch feather-duster

Serpulidae

Apomatus geniculatus (Moore & Bush, 1904) - pearl-topped calcareous worm
Crucigera zygophora (Johnson, 1901) - pale calcareous tubeworm
***Pileolaria* spp.** - dwarf calcareous tubeworm
Protula pacifica Pixell, 1912 - white-crown calcareous tubeworm
Pseudochitinopoma occidentalis (Bush, 1905) - western calcareous tubeworm
Salmacina tribranchiata (Moore, 1923) - three-branch calcareous tubeworm
Serpula columbiana Johnson, 1901 - red-trumpet calcareous tubeworm

Spionida

Chaetopteridae

***Chaetopterus variopedatus* complex** - U-shaped parchment tubeworm
Phyllochaetopterus prolifica Potts, 1914 - prolific three-section tubeworm
***Phyllochaetopterus* sp.** - three-section tubeworm
Spiochaetopterus costarum (Claparède, 1869) - jointed three-section tubeworm

Terebellida

Cirratulidae

Cirratulus spectabilis (Kinberg, 1866) - red threads worm
Dodecaceria concharum Örsted, 1843 - coralline-encased filament-worm

Dodecaceria fewkesi Berkeley & Berkeley, 1954 - fringed filament-worm

TEREBELLIDAE

Neoleprea spiralis (Johnson, 1901) - spaghetti worm

Pista elongata Moore, 1909 - basket-top spaghetti worm

***Thelepus* spp.** - curly-head spaghetti worm

SIPUNCULA – PEANUT WORMS

Sipunculidea

Golfingiida

GOLFINGIIDAE

Golfingia vulgaris (de Blainville, 1827) - brown peanut worm

ARTHROPODA – ARTHROPODS

Crustacea – crustaceans

Hexanauplia

Balanomorpha

BALANIDAE

Balanus crenatus Bruguière, 1789 - common acorn barnacle

Balanus glandula Darwin, 1854 - common acorn barnacle

Balanus nubilus Darwin, 1854 - giant acorn barnacle

Balanus rostratus Hoek, 1883 - large acorn barnacle

Semibalanus cariosus (Pallas, 1788) - thatched acorn barnacle

CHTHAMALIDAE

Chthamalus dalli Pilsbry, 1916 - small acorn barnacle

Siphonostomatoida

CALIGIDAE

***Lepeophtheirus* sp.** - parasitic copepod

Malacostraca

Amphipoda

AMPELISCIDAE

Ampelisca hancocki J.L. Barnard, 1954 - amphipod

Ampelisca lobata Holmes, 1908 - amphipod

Ampelisca unsocalae J.L. Barnard, 1960 - amphipod

AMPITHOIDAE

Ampithoe lacertosa Spence Bate, 1858 - sea lettuce sea flea

CAPRELLIDAE

Caprella alaskana Mayer, 1903 - Alaskan skeleton shrimp

Caprella mutica Schurin, 1935 - Japanese skeleton shrimp

MELITIDAE

Desdimelita californica (Alderman, 1936) - amphipod

Desdimelita microdentata Jarrett & Bousfield, 1996 - amphipod

Photidae

Photis brevipes Shoemaker, 1942 - amphipod

Pleustidae

Chromopleustes oculatus (Holmes, 1908) - black-and-white sea flea

Gnathopleustes pachychaetus Bousfield & Hendrycks, 1995 - amphipod

Talitridae

Megalorchestia pugettensis (Dana, 1853) - Puget Sound beach flea

Traskorchestia traskiana (Stimpson, 1857) - Pacific beach hopper

Decapoda

Callianassidae

Neotrypaea californiensis (Dana, 1854) - bay ghost shrimp

Cancridae

Cancer productus Randall, 1840 - red rock crab

Glebocarcinus oregonensis (Dana, 1852) - pygmy rock crab

Metacarcinus gracilis (Dana, 1852) - graceful crab

Metacarcinus magister (Dana, 1852) - dungeness crab

Cheiragonidae

Telmessus cheiragonus (Tilesius, 1815) - helmet crab

Crangonidae

Crangon nigricauda Stimpson, 1856 - blacktail bay shrimp

Metacrangon munita (Dana, 1852) - coastal spinyhead

Epialtidae

Chorilia longipes Dana, 1851 - longhorn decorator crab

Pugettia gracilis Dana, 1851 - graceful kelp crab

Pugettia producta (Randall, 1840) - northern kelp crab

Scyra acutifrons Dana, 1851 - sharpnose crab

Hapalogastridae

Acantholithodes hispidus (Stimpson, 1860) - hairy-spined crab

Hapalogaster mertensii J.F. Brandt, 1850 - hairy crab

Oedignathus inermis (Stimpson, 1860) - granular claw crab

Placetron wosnessenskii Schalfeew, 1892 - scaled crab

Lithodidae

Cryptolithodes sitchensis J.F. Brandt, 1853 - umbrella crab

Cryptolithodes typicus J.F. Brandt, 1848 - butterfly crab

Lopholithodes foraminatus (Stimpson, 1859) - brown box crab

Lopholithodes mandtii J.F. Brandt, 1848 - Puget Sound king crab

Phyllolithodes papillosus J.F. Brandt, 1848 - heart crab

Rhinolithodes wosnessenskii J.F. Brandt, 1848 - rhinoceros crab

Munididae

Munida quadrispina Benedict, 1902 - squat lobster

Oregoniidae

Hyas lyratus Dana, 1851 - Pacific lyre crab

Oregonia gracilis Dana, 1851 - graceful decorator crab

Paguridae

Discorsopagurus schmitti (Stevens, 1925) - tubeworm hermit
Elassochirus gilli (Benedict, 1892) - orange hermit
Elassochirus tenuimanus (Dana, 1851) - widehand hermit
Pagurus armatus (Dana, 1851) - blackeyed hermit
Pagurus beringanus (Benedict, 1892) - Bering hermit
Pagurus caurinus Hart, 1971 - Greenmark hermit
Pagurus granosimanus (Stimpson, 1859) - grainy hermit crab
Pagurus hirsutiusculus (Dana, 1851) - hairy hermit
Pagurus kennerlyi (Stimpson, 1864) - bluespine hermit
Pagurus ochotensis Brandt, 1851 - Alaskan hermit
Pagurus quaylei Hart, 1971 - Quayle's hermit
Pagurus samuelis (Stimpson, 1857) - blueband hermit
Pagurus stevensae Hart, 1971 - Stevens' hermit

PANDALIDAE

Pandalus borealis Krøyer, 1838 - northern shrimp
Pandalus danae Stimpson, 1857 - coonstripe shrimp
Pandalus eous Makarov, 1935 - spiny pink shrimp
Pandalus lucidirimicolus (Jensen, 1998) - sparkling shrimp
Pandalus platyceros J.F. Brandt in von Middendorf, 1851 - Pacific prawn
Pandalus stenolepis Rathbun, 1902 - rough patch shrimp

PANOPEIDAE

Lophopanopeus bellus (Stimpson, 1860) - black-clawed crab

PINNOTHERIDAE

Fabia subquadrata Dana, 1851 - pea crab

PORCELLANIDAE

Petrolisthes eriomerus Stimpson, 1871 - flattop crab

SERGESTIDAE

Eusergestes similis (Hansen, 1903) - Pacific sergestid

THORIDAE

Eualus butleri Jensen, 2004 - sponge eualid
Heptacarpus brevirostris (Dana, 1852) - stout coastal shrimp
Heptacarpus kincaidi (Rathbun, 1902) - Kincaid's shrimp
Heptacarpus sitchensis (J.F. Brandt in von Middendorf, 1851) - red-banded transparent shrimp
Heptacarpus stylus (Stimpson, 1864) - stiletto shrimp
Heptacarpus tridens (Rathbun, 1902) - threespine shrimp
Lebbeus eludus Jensen, 2006 - elusive lebbeid
Lebbeus grandimanus (Bražnikov, 1907) - candy stripe shrimp
Lebbeus mundus Jensen, 2006 - cleaner lebbeid
Spirontocaris prionota (Stimpson, 1864) - deep blade shrimp

VARUNIDAE

Hemigrapsus nudus (Dana, 1851) - purple shore crab
Hemigrapsus oregonensis (Dana, 1851) - yellow shore crab

Euphausiacea

EUPHAUSIIDAE

***Euphausia* spp.** - krill

Isopoda

Bopyridae

Bopyroides hippolytes (Kröyer, 1838) - shrimp parasitic isopod

Idoteidae

Pentidotea resecata (Stimpson, 1857) - eelgrass isopod

Pentidotea wosnesenskii (Brandt, 1851) - rockweed isopod

Sphaeromatidae

Gnorimosphaeroma oregonense (Dana, 1853) - Oregon pillbug

Ichthyostraca

Arguloida

Argulidae

Argulus pugettensis Dana, 1852 - surfperch fish louse

Entoprocta – entoprocts

Barentsiidae

***Barentsia* sp.** - nodding heads

Brachiopoda – brachiopods

Rhynchonelliformea

Rhynchonellata

Terebratulida

Terebrataliidae

Terebratalia transversa (Sowerby, 1846) - transverse lamp shell

Bryozoa – bryozoans

Gymnolaemata

Cheilostomatida

Bugulidae

Bugulina californica (Robertson, 1905) - spiral bryozoan

Caulibugula californica Robertson, 1905 - parasol bryozoan

Dendrobeania lichenoides (Robertson, 1900) - leaf crust bryozoan

Dendrobeania murrayana (Bean in Johnston, 1847) - fan bryozoan

Cellariidae

Cellaria diffusa Robertson, 1905 - spindly rabbit-ear bryozoan

Celleporidae

Celleporina robertsoniae (Canu & Bassler, 1923) - rusty bryozoan

Membraniporidae

Membranipora villosa Hincks, 1880 - kelp-encrusting bryozoan

Pacificincolidae

Primavelans insculpta (Hincks, 1883) - fluted bryozoan

PHIDOLOPORIDAE

Phidolopora pacifica (Robertson, 1908) - lattice-work bryozoan

SCHIZOPORELLIDAE

Schizoporella japonica Ortmann, 1890 - orange ripple bryozoan

Ctenostomatida

ALCYONIDIIDAE

Alcyonidium pedunculatum Robertson, 1902 - smooth leather bryozoan

VESICULARIIDAE

***Amathia* sp.** - tubeworm fuzz

Stenolaemata

Cyclostomatida

CRISIIDAE

***Crisia* spp.** - white tuft bryozoans

Diaperoforma californica (d'Orbigny, 1853) - southern staghorn bryozoan

HETEROPORIDAE

Heteropora alaskensis Borg, 1933 - delicate staghorn bryozoan

Heteropora pacifica Borg, 1933 - northern staghorn bryozoan

LICHENOPORIDAE

Disporella separata Osburn, 1953 - purple encrusting bryozoan

PHORONIDA – HORSESHOE WORMS

PHORONIDAE

Phoronis ijimai Oka, 1897 - white colonial Phoronid

ECHINODERMATA – ECHINODERMS

Asterozoa

Asteroidea

Forcipulatida

ASTERIIDAE

Evasterias troschelii (Stimpson, 1862) - mottled star

Leptasterias hexactis (Stimpson, 1862) - colourful six-armed star

Orthasterias koehleri (deLoriol, 1897) - rainbow star

Pisaster brevispinus (Stimpson, 1857) - giant pink star

Pisaster ochraceus (Brandt, 1835) - ochre star

Pycnopodia helianthoides (Brandt, 1835) - sunflower star

Stylasterias forreri (deLoriol, 1887) - velcro star

Paxillosida

LUIDIIDAE

Luidia foliolata Grube, 1866 - sand star

Spinulosida

ECHINASTERIDAE

Henricia aspera aspera Fisher, 1906 - ridged blood star
***Henricia leviuscula* complex** - Pacific blood star
Henricia sanguinolenta (O.F. Müller, 1776) - fat blood star

Velatida

PTERASTERIDAE

Pteraster tesselatus Ives, 1888 - slime star

Valvatida

ASTEROPSEIDAE

Dermasterias imbricata (Grube, 1857) - leather star

GONIASTERIDAE

Ceramaster patagonicus (Sladen, 1889) - orange cookie star
Hippasteria phrygiana (Parelius, 1768) - spiny red star
Mediaster aequalis Stimpson, 1857 - vermilion star

SOLASTERIDAE

Crossaster papposus (Linnaeus, 1767) - rose star
Solaster dawsoni Verrill, 1880 - morning sun star
Solaster endeca (Linnaeus, 1771) - northern sun star
Solaster stimpsoni Verrill, 1880 - striped sun star

Ophiuroidea

Amphilepidida

AMPHIURIDAE

Amphiodia periercta H.L. Clark, 1911 - long-armed burrowing brittle star

OPHIOPHOLIDAE

Ophiopholis kennerlyi Lyman, 1860 - daisy brittle star

Euryalida

GORGONOCEPHALIDAE

Gorgonocephalus eucnemis (Müller & Troschel, 1842) - basket star

Ophiurida

OPHIURIDAE

Ophiura luetkenii (Lyman, 1860) - gray brittle star
Ophiura sarsii Lütken, 1855 - notched brittle star

Crinozoa

Crinoidea

Comatulida

ANTEDONIDAE

Florometra serratissima (AH Clark, 1907) - feather star

Echinozoa

Echinoidea

Camarodonta

STRONGYLOCENTROTIDAE

Mesocentrotus franciscanus (A. Agassiz, 1863) - red sea urchin
Strongylocentrotus droebachiensis (O.F. Müller, 1776) - green sea urchin

Strongylocentrotus pallidus (G.O. Sars, 1871) - white sea urchin

Strongylocentrotus purpuratus (Stimpson, 1857) - purple urchin

Clypeasteroida

Dendrasteridae

Dendraster excentricus (Eschscholtz, 1831) - eccentric sand dollar

Holothuroidea

Apodida

Synaptidae

Leptosynapta transgressor Heding, 1928 - subtidal burrowing sea cucumber

Dendrochirotida

Cucumariidae

Cucumaria miniata (Brandt, 1835) - red sea cucumber

Cucumaria pallida Kirkendale & Lambert, 1995 - pale sea cucumber

Cucumaria piperata (Stimpson, 1864) - salt & pepper sea cucumber

Phyllophoridae

***Pentamera* sp.** - sea cucumber

Psolidae

Psolidium bidiscum Lambert, 1996 - pale creeping pedal sea cucumber

Psolus chitonoides Clark, 1901 - creeping pedal sea cucumber

Psolus squamatus (O.F. Müller, 1776) - white creeping pedal sea cucumber

Sclerodactylidae

Eupentacta quinquesemita (Selenka, 1867) - stiff-footed sea cucumber

Synallactida

Stichopodidae

Apostichopus californicus (Stimpson, 1857) - giant sea cucumber

Chordata – chordates

Tunicata – tunicates

Ascidiacea

Aplousobranchia

Clavelinidae

Clavelina huntsmani Van Name, 1931 - light-bulb tunicate

Pycnoclavella stanleyi Berrill & Abbott, 1949 - yellow social ascidian

Didemnidae

Didemnum carnulentum Ritter & Forsyth, 1917 - Pacific white crust

***Didemnum* spp.** - white glove leather

Didemnum vexillum Kott, 2002 - carpet sea squirt

Diplosoma listerianum (Milne Edwards, 1841) - gray encrusting compound tunicate

Trididemnum alexi Lambert, 2003 - speckled compound tunicate

Holozoidae

Distaplia occidentalis Bancroft, 1899 - mushroom compound tunicate

Polycitoridae

Cystodytes lobatus (Ritter, 1900) - lobed compound tunicate
Eudistoma molle (Ritter, 1900) - red-dotted compound tunicate
Eudistoma purpuropunctatum Lambert, 1989 - mauve lobed compound tunicate

POLYCLINIDAE

Aplidium californicum (Ritter & Forsyth, 1917) - California sea pork
Aplidium kottae Brunetti, 2007 - red ascidian
Synoicum parfustis (Ritter & Forsyth, 1917) - peach-coloured compound tunicate

Phlebobranchia

ASCIDIIDAE

Ascidia columbiana (Huntsman, 1912) - sea blisters
Ascidia paratropa (Huntsman, 1912) - glassy tunicate

CIONIDAE

Ciona savignyi Herdman, 1882 - sea vase

CORELLIDAE

Chelyosoma productum Stimpson, 1864 - disc-top tunicate
Corella inflata Huntsman, 1912 - brooding transparent tunicate
Corella willmeriana Herdman, 1898 - transparent tunicate

PEROPHORIDAE

Perophora annectens Ritter, 1893 - yellow social tunicate

Stolidobranchia

MOLGULIDAE

Molgula pacifica (Huntsman, 1912) - globular ascidian

PYURIDAE

Bathypera feminalba Young & Vazquez, 1995 - tiny white tunicate
Boltenia villosa (Stimpson, 1864) - hairy tunicate
Halocynthia aurantium (Pallas, 1787) - Pacific sea peach
Halocynthia igaboja Oka, 1906 - bristly tunicate
Pyura haustor (Stimpson, 1864) - warty tunicate
Pyura mirabilis (Drasche, 1884) - Aladdin's lamp tunicate

STYELIDAE

Botrylloides violaceus Oka, 1927 - lined compound ascidian
Cnemidocarpa finmarkiensis (Kiaer, 1893) - broadbase tunicate
Metandrocarpa taylori Huntsman, 1912 - orange social tunicate
Styela gibbsii Stimpson, 1864 - brown tunicate
Styela montereyensis (Dall, 1872) - stalked tunicate

Vertebrata – vertebrates

Actinopterygii – ray-finned fishes

Batrachoidiformes

BATRACHOIDIDAE

Porichthys notatus Girard, 1854 - plainfin midshipman

Clupeiformes

CLUPEIDAE

Clupea pallasii Valenciennes, 1847 - Pacific herring

ENGRAULIDAE

Engraulis mordax Girard, 1854 - northern anchovy

Gadiformes

GADIDAE

Gadus chalcogrammus Pallas, 1814 - walleye pollock

Gadus macrocephalus Tilesius, 1810 - Pacific cod

Gobiesociformes

GOBIESOCIDAE

Gobiesox maeandricus (Girard, 1858) - northern clingfish

Gobiiformes

GOBIIDAE

Clevelandia ios (Jordan & Gilbert, 1882) - arrow goby

Rhinogobiops nicholsii (Bean, 1882) - blackeye goby

Lampriformes

TRACHIPTERIDAE

Trachipterus altivelis Kner, 1859 - king-of-the-salmon

Ophidiiformes

BYTHITIDAE

Brosmophycis marginata (Ayres, 1854) - red brotula

Perciformes

AGONIDAE

Agonopsis vulsa (Jordan & Gilbert, 1880) - northern spearnose poacher

Odontopyxis trispinosa Lockington, 1880 - pygmy poacher

Podothecus accipenserinus (Tilesius, 1813) - sturgeon poacher

AMMODYTIDAE

Ammodytes personatus Girard, 1856 - Pacific sand lance

ANARHICHADIDAE

Anarrhichthys ocellatus Ayres, 1855 - wolf-eel

AULORHYNCHIDAE

Aulorhynchus flavidus Gill, 1861 - tubesnout

BATHYMASTERIDAE

Ronquilus jordani (Gilbert, 1889) - northern ronquil

COTTIDAE

Artedius fenestralis Jordan & Gilbert, 1883 - padded sculpin

Artedius harringtoni (Starks, 1896) - scalyhead sculpin

Artedius lateralis (Girard, 1854) - smoothhead sculpin

Asemichthys taylori Gilbert, 1912 - spinynose sculpin

Chitonotus pugetensis (Steindachner, 1876) - roughback sculpin

Enophrys bison (Girard, 1854) - buffalo sculpin

Hemilepidotus hemilepidotus (Tilesius, 1811) - red Irish lord

Icelinus tenuis Gilbert, 1890 - spotfin sculpin

Jordania zonope Starks, 1895 - longfin sculpin

Leptocottus armatus Girard, 1854 - Pacific staghorn sculpin

Myoxocephalus polyacanthocephalus (Pallas, 1814) - great sculpin
Oligocottus maculosus Girard, 1856 - tidepool sculpin
Scorpaenichthys marmoratus (Ayres, 1854) - cabezon
Synchirus gilli Bean, 1890 - manacled sculpin
Triglops macellus (Bean, 1884) - roughspine sculpin

EMBIOTOCIDAE

Brachyistius frenatus Gill, 1862 - kelp perch
Cymatogaster aggregata Gibbons, 1854 - shiner perch
Embiotoca lateralis Agassiz, 1854 - striped seaperch
Rhacochilus vacca (Girard, 1855) - pile perch

GASTEROSTEIDAE

Gasterosteus aculeatus Linnaeus, 1758 - three-spined stickleback

HEXAGRAMMIDAE

Hexagrammos decagrammus (Pallas, 1810) - kelp greenling
Hexagrammos stelleri Tilesius, 1810 - whitespotted greenling
Ophiodon elongatus Girard, 1854 - lingcod
Oxylebius pictus Gill, 1862 - painted greenling

LIPARIDAE

Liparis dennyi Jordan & Starks, 1895 - marbled snailfish
Liparis pulchellus Ayres, 1855 - showy snailfish

PHOLIDAE

Apodichthys flavidus Girard, 1854 - penpoint gunnel
Pholis clemensi Rosenblatt, 1964 - longfin gunnel
Pholis laeta (Cope, 1873) - crescent gunnel

PTILICHTHYIDAE

Ptilichthys goodei Bean, 1881 – quillfish

PSYCHROLUTIDAE

Psychrolutes sigalutes (Jordan & Starks, 1895) - soft sculpin

SEBASTIDAE

Sebastes auriculatus Girard, 1854 - brown rockfish
Sebastes caurinus Richardson, 1844 - copper rockfish
Sebastes diploproa (Gilbert, 1890) - splitnose rockfish
Sebastes elongatus Ayres, 1859 - greenstriped rockfish
Sebastes emphaeus (Starks, 1911) - Puget Sound rockfish
Sebastes flavidus (Ayres, 1862) - yellowtail rockfish
Sebastes maliger (Jordan & Gilbert, 1880) - quillback rockfish
Sebastes melanops Girard, 1856 - black rockfish
Sebastes nigrocinctus Ayres, 1859 - tiger rockfish
Sebastes proriger (Jordan & Gilbert, 1880) - redstripe rockfish
Sebastes ruberrimus (Cramer, 1895) - yellow-eye rockfish

STICHAEIDAE

Anoplarchus purpurescens Gill, 1861 - high cockscomb
Chirolophis decoratus (Jordan & Snyder, 1902) - decorated warbonnet
Chirolophis nugator (Jordan & Williams, 1895) - mosshead warbonnet

Lumpenus sagitta Wilimovsky, 1956 - snake prickleback
Phytichthys chirus (Jordan & Gilbert, 1880) - ribbon prickleback
Xiphister mucosus (Girard, 1858) - rock eel

RHAMPHOCOTTIDAE

Rhamphocottus richardsonii Günther, 1874 - grunt sculpin

Pleuronectiformes

PARALICHTHYIDAE

Citharichthys stigmaeus Jordan & Gilbert, 1882 - speckled sanddab

PLEURONECTIDAE

Lepidopsetta bilineata (Ayres, 1855) - rock sole
Lyopsetta exilis (Jordan & Gilbert, 1880) - slender sole
Parophrys vetulus Girard, 1854 - English sole
Platichthys stellatus (Pallas, 1787) - starry flounder
Pleuronichthys coenosus Girard, 1854 - C-O sole

Salmoniformes

SALMONIDAE

Oncorhynchus clarkii (Richardson, 1836) - cutthroat trout
Oncorhynchus keta (Walbaum, 1792) - chum salmon
Oncorhynchus kisutch (Walbaum, 1792) - coho salmon
Oncorhynchus tshawytscha (Walbaum, 1792) - chinook salmon

Scorpaeniformes

HEMITRIPTERIDAE

Nautichthys oculofasciatus (Girard, 1858) - sailfin sculpin

Syngnathiformes

SYNGNATHIDAE

Syngnathus leptorhynchus Girard, 1854 - bay pipefish

Elasmobranchii - sharks, rays, skates

Rajiformes

RAJIDAE

Beringraja binoculata (Girard, 1855) - big skate
Beringraja rhina (Jordan & Gilbert, 1880) - longnose skate

Squaliformes

SQUALIDAE

Squalus suckleyi (Girard, 1855) - Pacific spiny dogfish shark

Holocephali - chimera

Chimaeriformes

CHIMAERIDAE

Hydrolagus colliei (Lay & Bennett, 1839) - spotted ratfish

Mammalia - mammals

Artiodactyla

BALAENOPTERIDAE

Balaenoptera acutorostrata Lacépède, 1804 - northern minke whale
Balaenoptera physalus (Linnaeus, 1758) - fin whale

Megaptera novaeangliae (Borowski, 1781) - humpback whale

DELPHINIDAE

Lagenorhynchus obliquidens Gill, 1865 - Pacific white-sided dolphin

Orcinus orca (Linnaeus, 1758) - orca

ESCHRICHTIIDAE

Eschrichtius robustus (Lilljeborg, 1861) - grey whale

PHOCOENIDAE

Phocoena phocoena (Linnaeus, 1758) - harbour porpoise

Phocoenoides dalli (True, 1885) - Dall's porpoise

Carnivora

MUSTELIDAE

Lontra canadensis Schreber, 1777 - North American river otter

Neogale vison (Schreber, 1777) - American mink

OTARIIDAE

Eumetopias jubatus (Schreber, 1776) - Steller sea lion

Zalophus californianus (Lesson, 1828) - California sea lion

PHOCIDAE

Phoca vitulina richardii Linnaeus, 1758 - Pacific harbor seal
